# Unveiling the Activation Pathway of the CO_2_ Reduction Catalyst *trans*(Cl)-[Ru(X,X′-dimethyl-2,2′-bipyridine)(CO)_2_Cl_2_] by Direct Spectroscopic Observation

**DOI:** 10.1021/acscatal.4c06974

**Published:** 2025-02-05

**Authors:** Sergio Aranda-Ruiz, Luka Tatarashvili, Kerstin Oppelt, Peter Hamm

**Affiliations:** Department of Chemistry, University of Zürich, Zürich CH-8057, Switzerland

**Keywords:** CO_2_ reduction, Ru catalyst, hydride, time-resolved IR spectroscopy, lifetime analysis

## Abstract

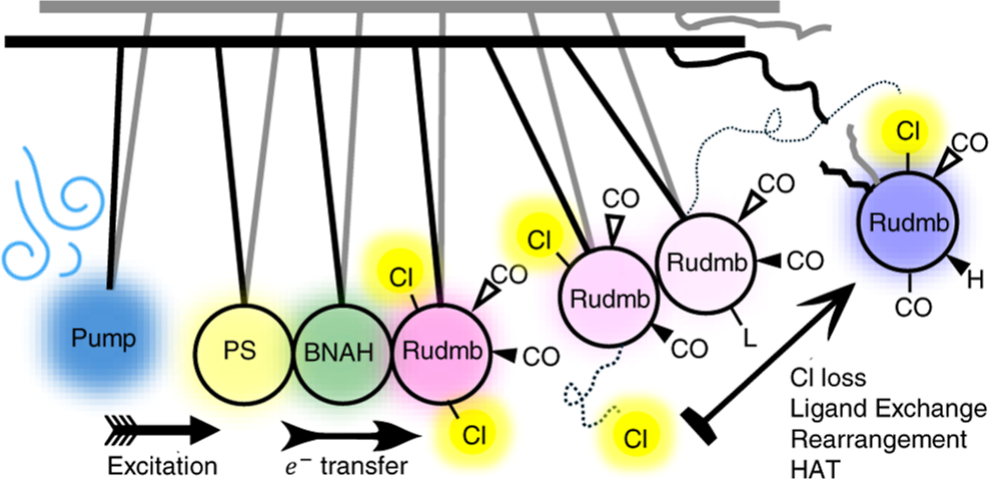

We report on the activation pathway of a series of CO_2_ reduction catalysts, *trans*(Cl)-[Ru(X,X′-dimethyl-2,2′-bipyridine)(CO)_2_Cl_2_], with a focus on *trans*(Cl)-[Ru(6,6′-dimethyl-2,2′-bipyridine)(CO)_2_Cl_2_]), in the presence of the reductive quencher 1-benzyl-1,4-dihydronicotinamide and the photosensitizer Ru(bpy)_3_Cl_2_. Most mechanistic studies of these types of catalytic systems use spectroelectrochemistry in the IR, where the vibrational frequencies of the carbonyl vibrations report on the electron density on the metal center. However, spectroelectrochemistry may miss short-lived intermediates, while at the same time the spectra can be dominated by accumulating side-products, which may play only a minor role in the reaction cycle. Transient IR spectroscopy on all relevant time scales, from picoseconds to hundreds of milliseconds, can bridge this gap, revealing a surprisingly complex reaction pathway (in combination with NMR spectroscopy as well as DFT calculations). That is, electron transfer from the reduced photosensitizer is followed by a loss of a first chloride ligand, a replacement of the second chloride ligand by a solvent molecule, and a ligand rearrangement that releases the strain between the equatorial carbonyl ligands and the methyl group on the bpy ligand in this catalyst. These reaction steps happen on a tens of nanoseconds to tens of microseconds time scale. In the case of *trans*(Cl)-[Ru(6,6′-dimethyl-2,2′-bipyridine)(CO)_2_Cl_2_]), the complex is then reduced a second time from the oxidized 1-benzyl-1,4-dihydronicotinamide on a significantly slower 10–100 ms time scale, protonated and the solvent ligand is exchanged back to a chloride. The final product hence is a hydride, Ru^II^(6,6′-dmbpy)(CO)_2_ClH, which is stable on a minute-to-hour time scale. In case of *trans*(Cl)-[Ru(5,5′-dmbpy)(CO)_2_Cl_2_]), dimerization of the reduced species is possible, which eventually leads to the formation of *cis*(Cl)-[Ru(5,5′-dmbpy)(CO)_2_Cl_2_]. The work illustrates the power of transient IR spectroscopy to elucidate complex reaction pathways of such catalytic systems, and provides solid cornerstones for their kinetic control.

## Introduction

Growing concerns about the accumulation of greenhouse gases, in particular CO_2_, have drawn increasing attention.^1,2^ To address this issue, three ways of reducing atmospheric CO_2_ - its capture, decreasing its production, and utilizing CO_2_ - have been proposed.^3^ The latter approach is appealing since atmospheric CO_2_ is a renewable and abundant C_1_ building block. However, using CO_2_ to produce other chemicals or fuels poses a significant challenge. CO_2_ is a very stable molecule and its conversion to different products requires a high thermodynamic cost.^4^ One-electron reduction of CO_2_ needs 1.9 eV vs SHE of free energy at standard ambient temperature and pressure in aqueous solutions. The cost is significantly reduced by a proton-coupled multielectron transfer
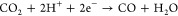
1that requires only 0.53 eV instead.^5^ To facilitate such a multielectron process, a “platform” with multiple available redox states is required. In that regard, transition metal complexes as catalysts have attracted increasing interest since the 1980s.^6^ Simply speaking, these catalysts serve as hosts for CO_2_, which binds through its electrophilic carbon onto the metal center. This requires a structural rearrangement of the molecule, from a linear to a bent geometry, as the C–O bond order decreases. This step - typically referred to as “CO_2_ activation” - is energetically very demanding, particularly in the absence of protons.^7,8^

Several catalytic methods exist for producing different value-added chemicals from CO_2_.^9^ Arguably the most versatile product of CO_2_ reduction is CO, which in turn can be used as feedstock for the Fischer–Tropsch reaction.^10^ Many metal complexes have been investigated over the last few decades for catalyzing CO_2_ reduction, including ones based on cobalt,^11,12^ iron,^13,14^ manganese,^15,16^ rhodium,^17,18^ iridium,^19,20^ rhenium,^21−23^ ruthenium,^24−27^ tungsten and molybdenum.^28^ This list is not comprehensive and a more detailed overview can be found in recent review articles.^29−34^

Ruthenium 2,2′-bipyridine (hereafter bpy) dicarbonyl dichloride and derivatives have been reported as very good catalysts for both electro- and photochemical CO_2_ reduction. For this type of catalysts, spectroelectrochemistry in the IR is a very revealing spectroscopic method, since they have metal carbonyls, which exhibit strong IR absorption cross-section and are sensitive to the changes in electron density on the metal due to its back bonding properties. For example, combining the previous knowledge about the mechanisms of CO and formate formation,^35−37^ Ishida and co-workers have done extensive studies on Ru(bpy)(CO)_2_Cl_2_, reporting a new possible mechanism and elucidating the origin of CO vs formate production based on whether the catalyst undergoes dimerization. This mechanism highlights the fact that a decrease in the concentration of the ground state catalyst, Ru(II), will also reduce the ratio of CO/formate produced. This study has shown that formate production occurs through the dimerization of one reduced catalyst molecule with another one, which is catalytically active. Later, they also explored how the electrochemical properties and catalytic activities are affected by substituting methyl groups on the bpy ligand (dmbpy) in three different configurations (4,4′-, 5,5′-, and 6,6′-dimethyl bipyridine).^38^ From their results, the 6,6′-dimethyl isomer of the catalyst—hereafter referred to as Ru6dmb—was determined to be the most CO-selective due to the deformation of its bipyridine ligand preventing dimerization. Ru6dmb is the focus of the present study.

Similarly, the *trans*(Cl)-[Ru(mesbpy)(CO)_2_Cl_2_] molecule with an even bulkier 6,6′-dimesityl-2,2′-bipyridine ligand has been studied using spectroelectrochemistry^39^ and the two strongest carbonyl bands of the singly reduced species were assigned to a replacement of a Cl^–^ ligand by a solvent molecule. However, a range of other minor bands were also observed, whose assignment was not clear. Studies conducted by the Alberto group^40^ rely on these previous assignments, but also see several extra bands that were not assigned specifically.

Spectroelectrochemistry can miss short-lived intermediates, while at the same time the spectra may be dominated by accumulating side products, which might play only a minor role in the reaction cycle per se. In particular, species with more positive potentials that are generated during the reaction undergo further reduction at the electrode, making their isolation difficult. Providing up to picosecond time resolution, we will see that transient IR (TRIR) spectroscopy can effectively bridge this gap by highlighting early, and hence potentially relevant species as they appear during the course of the reaction cycle. TRIR spectroscopy has been used for mechanistic studies for photocatalytic CO_2_ reduction before, but only to a rather limited extent.^41−46^ Here, we will apply TRIR spectroscopy to investigate Ru6dmb and related catalysts. It is established that these catalysts require a prereduction step to become catalytically active. We have investigated this process in unprecedented detail to obtain a better understanding of catalytic CO_2_ reduction. We will demonstrate that the ability to cover all relevant time scales in our TRIR experiments, from picoseconds to almost seconds, augmented with a “lifetime analysis”, is crucial to elucidate the diffusion controlled electron transfer and ligand exchange processes in a complex photocatalytic system, which consists of several components, i.e., a photosensitizer, an electron donor, a catalyst and a potentially ligating solvent.

## Methods

In general, Material and Methods are described in Supporting Information, but one aspect, “lifetime analysis”, is of particular relevance, and shall be briefly introduced here. As we will see in the course of the paper, none of the observed reaction steps are single-exponential, since they are second-order processes and/or heterogeneous with different, spectroscopically not separable conformers contribute. As a result, global multiexponential fitting turned out to be impossible. We consequently applied a “lifetime analysis”,^47−50^ which makes fewer model assumption, and which is a central aspect of the analysis of the experimental data. An account of the advantages and disadvantages of global multiexponential fitting vs lifetime analysis is given in Buhrke et al.^51^

In the lifetime analysis, the time traces at each probe-wavelength ω_*i*_ are fit by
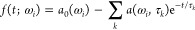
2which contains many exponential terms with time-constants τ_*k*_ that are distributed equally on a logarithmic time axis with 10 terms/decade. In contrast to global multiexponential fitting, which typically contains only very few such terms, the time-constants τ_*k*_ are not free fitting parameters in the lifetime analysis, only their amplitudes are, which give rise to a lifetime density map *a*(ω_*i*_, τ_*k*_). The fit is regularized with a maximum entropy method,^47−49^ otherwise it would be overfitting. To condense the information on the lifetime density map, we finally averaged over all probe-frequency positions in a way that positive and negative amplitudes do not cancel out
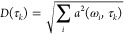
3and call *D*(τ_*k*_) the “dynamical content”.

**Figure 1:**
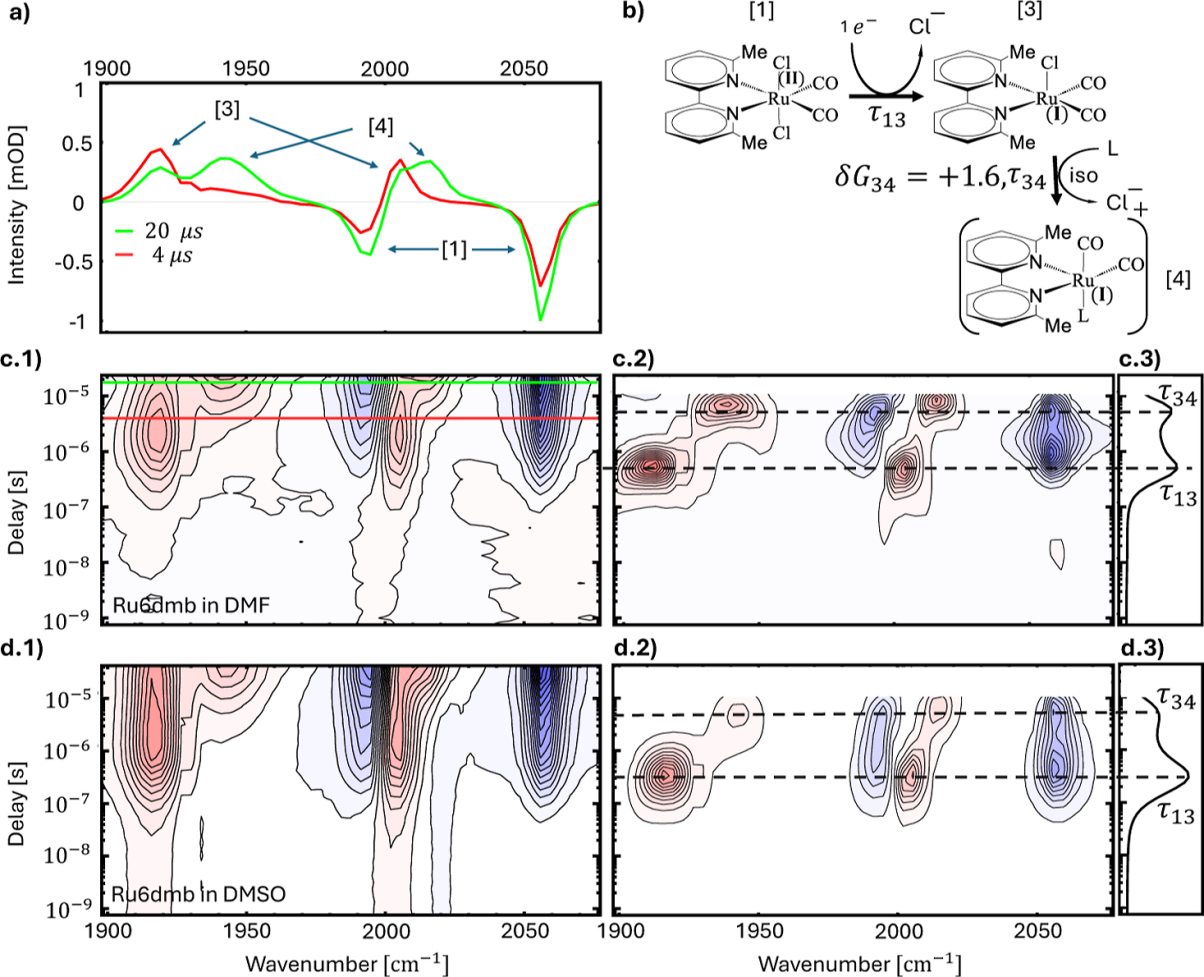
TRIR spectra of Ru6dmb in DMF [c(1)] and DMSO [d(1)] in the 1 ns to 40 μs time window, with the respective lifetime density maps [c(2),d(2)] and dynamical content [c(3),d(3)], as well as spectral cuts at selected delay times in the case of DMF (a). Panel (b) shows the reaction steps observed in these spectra. The time constants in [c(2)] are τ_13_ = 0.5 μs and τ_34_ = 5 μs, and in [d(2)]: τ_13_ = 0.3 μs and τ_34_ = 5 μs. Experimental conditions: 20 mM Ru6dmb, 10 mM Ru(bpy)_3_Cl_2_, 100 mM BNAH in DMF or DMSO, respectively, excitation wavelength 420 nm. The reaction free energy in panel (b) is given in units of kcal/mol, and has been calculated for DMF as solvent.

We illustrate the concept based on some of the data of Figure 1. For example, Figure 1c(2) illustrates how the lifetime analysis introduced above is applied to the data set of Figure 1c(1). Figure 1c(2) represents the 2D lifetime density map *a*(ω_*i*_, τ_*k*_). A positive (red) peak in the lifetime density map means that the absorbance in the corresponding time-trace increases at that time, while a negative (blue) peak means that the absorbance decreases. Figure 1c(3) shows how this information is condensed into the 1D “dynamical content” according to eq 3. A peak in the dynamical content indicates a kinetic process with the corresponding time-constant. The lifetime analysis leading to the dynamical content is an essentially model-free technique, not relying on any assumption whether a kinetic process is first-order (i.e., exponential) or second-order; the peak would just become broader in the second case, reflecting a distribution of lifetimes.

As a word of caution, it needs to be added that the maximum entropy method includes a critical parameter, the relative weight of the RMSD of the fit versus the regularization function stabilizing the fit. Proper tuning of that weighting factor avoids overfitting, albeit at the cost of the resolution to separate close-lying kinetic processes (for a detailed discussion see refs (47–50)). In each of the data sets shown in this paper, conservative weighting factors were chosen to safely avoid overfitting, and were tuned such that the width (in time) of peaks in the lifetime spectra is about the same. Furthermore, the lifetime analysis is not very reliable at the fast-time and slow-time edges of the investigated time window, and the shown lifetime spectra are cut accordingly.

## Results and Discussion

A typical chemical system for a photoinduced homogeneous catalysis involves three components: A photosensitizer (hereafter PS) that converts light energy into an electrochemical potential, an electron donor, that reductively (in most cases) quenches the PS, followed by an electron transfer from PS^•–^ to the catalyst.^52^ The particular chemical system investigated here consists of 20 mM of *trans*(Cl)-[Ru(6,6′-dimethyl-2,2′-bipyridine)(CO)_2_Cl_2_] as catalyst (in most cases, exceptions to this are mentioned explicitly), 10 mM of Ru(bpy)_3_Cl_2_ as PS, 100 mM of BNAH as sacrificial electron donor, as well as the solvent (DMF or DMSO). The sample is purged with Ar to eliminate contributions from CO_2_ and oxygen. Supply and synthesis of all compounds used in this study were adapted from literature reports,^38,53−60^ and are described in detail in Section S1 of the Supporting Information. To investigate the processes leading to the activation of the catalyst, i.e. the series of reaction steps involved in its reduction, we studied the spectral region around 2000 cm^–1^ with the help of TRIR spectroscopy, where the carbonyl (−C≡O) groups of the catalyst serve as IR spectroscopic reporters on the metal center‘s redox state, as well as of other ligands coordinated to it.

The PS strongly absorbs in the region of the 420–447 nm pump pulses, whereas both the reductive quencher and the catalyst have a significantly smaller absorbance (see Figure S5 in Supporting Information). Nonetheless, the absorbance of the catalyst is not completely negligible, in particular at 420 nm used in one of the laser setups as pump pulse. The catalyst releases CO upon electronic excitation (see Section S3 in Supporting Information),^61−63^ hence we needed to suppress the accumulation of photodegraded catalyst. One can avoid a direct excitation of the catalyst by shifting the excitation wavelength to the red as much as possible and by using the PS at comparably high concentration.

### Reduction and Chloride Loss

We used two different laser setups in this study (see Section S2 in Supporting Information for details), whose time-windows differ and match the two major reaction steps observed for the catalyst, reduction, chloride loss with ligand exchange and ligand rearrangement on the one hand and hydrogen atom transfer on the other hand. We start with the results from the first laser setup, covering a time window from 1 ns to ∼40 μs.^64^

**Figure 2:**
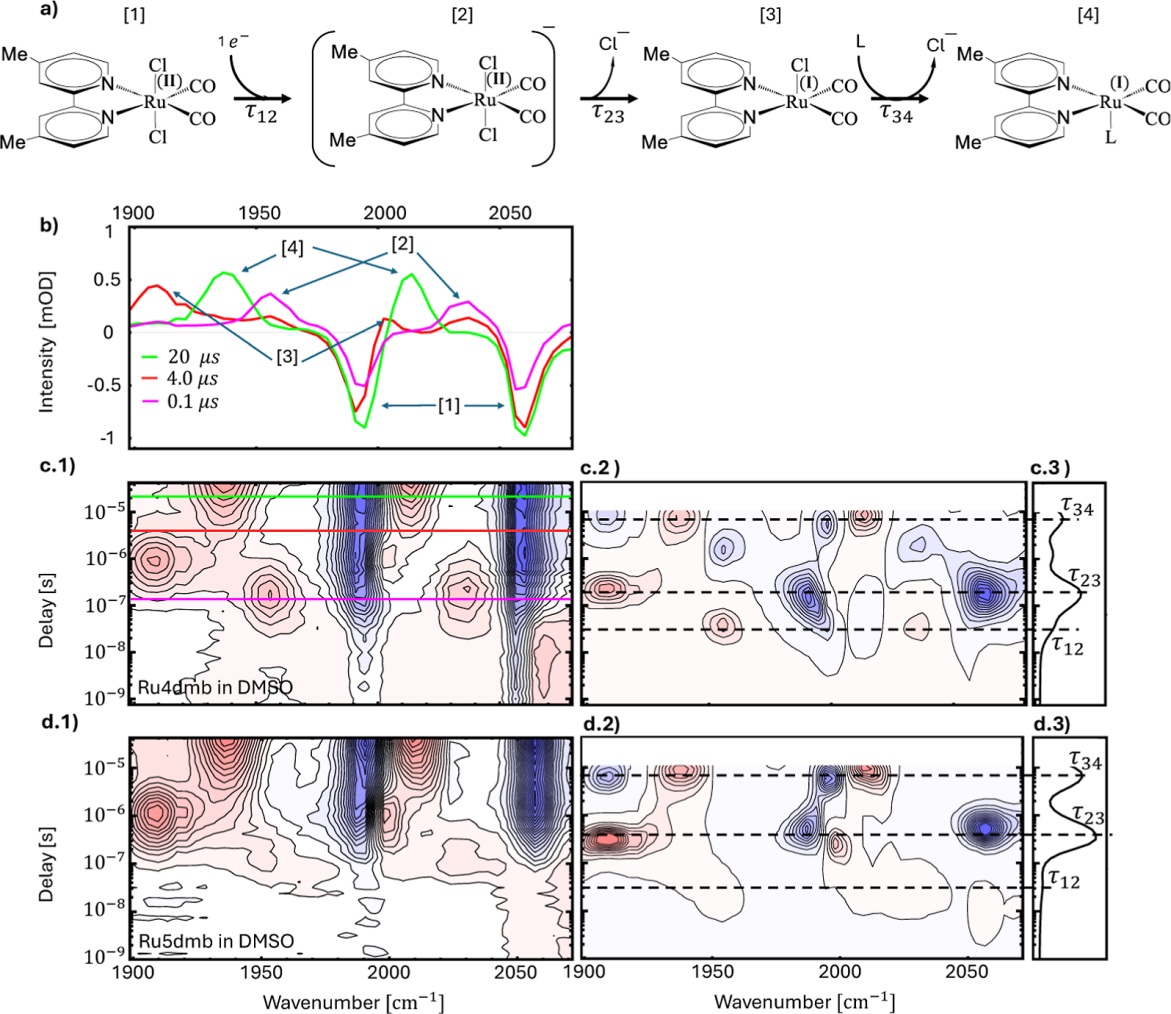
TRIR spectra of Ru4dmb [c(1)] and Ru5dmb [d(1)] in the 1 ns to 40 μs time window, with the respective lifetime density maps [c(2),d(2)] and dynamical content [c(3),d(3)], as well as spectral cuts at selected delay times (b) shown for Ru4dmb, as indicated by the equally colored horizontal lines in panel [c(1)]. Panel (a) shows the reaction steps observed in these spectra. The time constants in [c(2)] are τ_12_ = 30 ns, τ_23_ = 0.2 μs, and τ_34_ = 7 μs, and in [d(2)], τ_12_ = 30 ns, τ_23_ = 0.4 μs, and τ_34_ = 7 μs. Experimental conditions: 20 mM Ru4dmb or Ru5dmb, respectively, 10 mM Ru(bpy)_3_Cl_2_, 100 mM BNAH in DMSO, excitation wavelength 420 nm.

Figure 1c(1),d(1) show the TRIR spectra of Ru6dmb in DMF and DMSO in a 2D representation, as well as lifetime density maps in Figure 1c(2),d(2) and the dynamical content in Figure 1c(3),d(3). While we focus in this paper on DMF as solvent, some observations were possible in DMSO only (in particular the reduction step before the loss of the first chloride shown in Figure 2), which is why we show both here for comparison. Furthermore, Figure 1a shows for the sample in DMF spectral cuts at the delay times, at which the populations of the various intermediates are substantial, as indicated by the green and red horizontal lines in Figure 1c1.

Since we are analyzing difference spectra, positive bands depicted in red in Figure 1c(1),d(1) represent new species that are formed during the reaction, whereas the two negative bands (depicted in blue) originate from the original molecule. Every new species stemming from the catalyst results in depletion of the original, which in turn translates to its negative absorbance in the difference spectra. The two negative bands are attributed to symmetric (∼2056 cm^–1^) and antisymmetric (∼1990 cm^–1^) stretches of the carbonyls of Ru^II^(6,6′-dmbpy)(CO)_2_Cl_2_, the catalyst in its resting state (peaks **[1]** in the Figure 1a). Generally, for metal–carbonyl complexes, reduction of the metal center lowers the vibrational frequencies of the carbonyl stretching modes, owing to the back-bonding effect from the metal center to the −C≡O bond.^65^ The extent to which this is happening also depends on the other ligands.

In the first reaction step with a time constant τ_13_ = 0.5 μs (in DMF), two positive bands appear at ∼1917 and ∼2003 cm^–1^ (bands labeled as **[3]** in the Figure 1a), which are the frequency down-shifted counterparts of the two bands at 1990 and 2056 cm^–1^ of the resting state **[1]**. The interpretation of the 1917 cm^–1^ positive band is clearer, since it is free from any overlapping contribution.

Throughout the paper, we will use DFT calculations to identify certain transient intermediates, based on the match of the CO vibrational frequencies as well as the criterion whether they are energetically feasible. The DFT methods are described in Supporting Information, Section S7, together with Table S4, that summarizes the DFT results (energies and frequencies) of all intermediates that are potentially relevant in the reaction. Table 1 compares the experimental and calculated frequencies of all species that have been identified experimentally in a more compact form.

**Table 1:** Comparison of Experimental and Calculated Antisymmetric (as) and Symmetric (s) CO Stretching Frequencies (cm^–1^) for all Observed Species^a^

chemical species	experimental (as/s)	calculated (as/s)
Ru^II^(6,6′-dmbpy)(CO)_2_Cl_2_	1990/2056	1990/2056
Ru^II^(6,6′-dmbpy)(CO)_2_Cl H^(Equat.)^	1949/2025	1930/2010
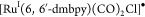	1917/2003	1908/1991
(iso)-	1943/2013	1915/2005
[Ru^II^(4,4′-dmbpy^•–^)(CO)_2_Cl_2_]^−^	1956/2037	1957/2030
[Ru^II^(5,5′-dmbpy^•–^)(CO)_2_Cl_2_]^−^	1951/2028	1955/2028
*trans*(Cl)-[Ru^II^(5,5′-dmbpy)(CO)_2_Cl_2_]	1994/2058	1988/2055
*cis*(Cl)-[Ru^II^(5.5′-dmbpy)(CO)_2_Cl_2_]	1994/2062	1990/2059

a For species with more than one conformer, the average of frequencies are reported, see Table S4 for details.

It should be added that the calculated vibrational frequencies need to be used with caution, as one can easily measure frequencies with a precision of about 2 cm^–1^, i.e., about 0.1% relative to the vibrational frequency, which is an unrealistic accuracy for any DFT method for a molecule of this size. We therefore look more for frequency up- or down-shifts at individual reaction steps, rather than absolute vibrational frequencies, which are relatively directly connected to the decrease or increase of charge density on the metal center due to the back-bonding effect, and for which DFT is in fact quite reliable.^66^

Based on the DFT results, we conclude that the appearance of this first positive band pair reflects the diffusion-controlled electron transfer from the PS^•–^ to the catalyst accompanied by a chloride loss. That is, the DFT-calculated frequencies of Ru^I^(6,6′-dmbpy)(CO)_2_Cl]^•^ are 1908 and 1991 cm^–1^. This is in reasonable agreement with the experimental observation, when keeping in mind that the 2003 cm^–1^ positive band is pushed to higher frequencies in the experimental difference spectrum due to partial overlap with the 1990 cm^–1^ negative band from the resting state. Despite being a bimolecular reaction, reductive quenching of the catalyst by the PS can be fast, since not-yet reduced catalyst is present at relatively high concentration (20 mM), which is significantly higher than photoexcited and hence reduced PS (1 mM, estimated from the excitation density). Thus, the reaction kinetics is pseudo-first order.

One would expect the first intermediate to appear directly after the reduction to be , which presumably is short-lived since it is a 19-electron complex. While we have no spectroscopic evidence for that species in the case of Ru6dmb, we can indeed identify that intermediate for two different versions of the catalyst with methyl substituents in the 4,4′- and 5,5′-positions (hereafter Ru4dmb and Ru5dmb, respectively), see Figure 2. In particular in the case of Ru4dmb, two even earlier peaks labeled as **[2]** appear within τ_12_ = 30 ns at 1956 and 2037 cm^–1^ [Figure 2b,c(1)], close to the frequencies predicted from the DFT results (see Table S4 in Supporting Information). That rate is close to the fastest possible diffusion-limited rate, which we estimated to be ≈1 × 10^8^ s^–1^ for the concentrations of the two reactants in the experiment. In the case of Ru5dmb, the concentration of the intermediate is barely enough to observe a faint absorbance at 1951 and 2028 cm^–1^ [Figure 2d(1)].

**Figure 3:**
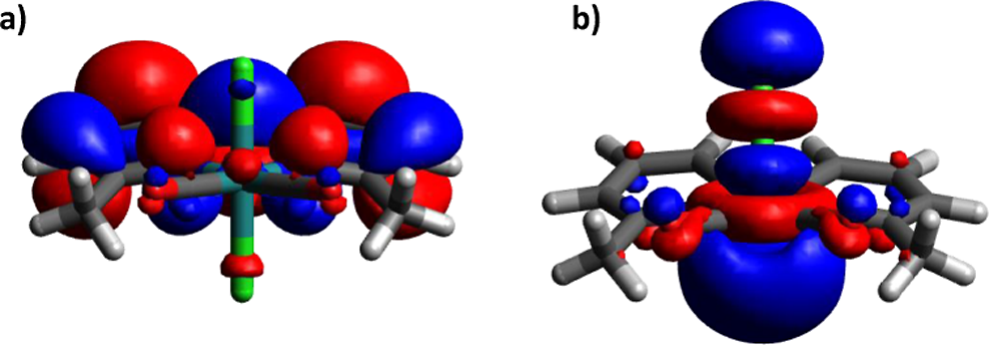
Kohn–Sham HOMOs of (a) Ru^II^(6,6′-dmbpy^•–^)(CO)_2_Cl_2_ and (b) . The catalyst is facing the reader with the two carbonyls.

One may find it counterintuitive that the complex after the loss of a chloride ligand, and with it a negative charge, should have lower CO vibrational frequencies than the 19-electron intermediate. This can be explained by comparing the HOMO orbitals of  and  (see Figure 3). It is clear that the former’s HOMO is ligand-centered which becomes metal-centered after the release of the chloride ligand. Based on that, it would be more appropriate to rewrite the singly reduced species as Ru^II^(6,6′-dmbpy^•–^)(CO)_2_Cl_2_, since the oxidation state of the metal center is hardly affected, as has been reported in the literature.^67^ The stabilization of the radical anion has been investigated before by Kubiak and co-workers in a Rhenium-carbonyl complex,^68,69^ showing that electron-donating substituents on the bpy destabilize it as it is already rich in electron density after the complex’s initial reduction. The same effect has been reported for Mn(I) complexes.^70^

In Ru6dmb, τ_23_ must be faster than τ_12_, which is why no measurable concentration of the corresponding intermediate builds up, and τ_12_ is the rate-limiting step for the overall reaction step τ_13_. In Ru4dmb, τ_12_ speeds up significantly (30 ns), hence τ_12_ now is faster than τ_23_. A possible reason could be the bending induced in the bpy rings due to steric hindrance between the methyl and CO groups, which is substantial only in Ru6dmb. Since the first electron transfer is stored in the bpy ring (as shown in Figure 3), a better π-conjugation could explain why this intermediate is more populated in Ru4dmb and Ru5dmb than in Ru6dmb, and therefore observed in the transient data.

It has been shown by Kuramochi et al.^38^ that the reduction potentials of Ru4dmb, Ru5dmb and Ru6dmb are nearly identical, yet the kinetics of the first reaction step τ_12_ differ by a factor 10, which might seem surprising. However, the results of Kuramochi et al.^38^ are deduced from cyclic voltammetry (CV) experiments, that do not have any time resolution. These experiments measure the reduction potentials for the formation of later reaction products that appear on the intrinsic time scale of CV, i.e., in the range of seconds. Assuming that all involved reaction steps are equilibrium reactions and not irreversible, transient intermediates on the pathway to those later reaction products then need to be considered transition states, which determine the kinetics of the overall reaction but not its thermodynamics. Hence, we do in fact not expect a one-to-one correlation between the overall reaction potential measured in a CV experiment and the time-constant of the first reaction step, whenever the latter is much faster than the speed of the overall reaction.

### Ligand Exchange and Rearrangement

**Figure 4:**
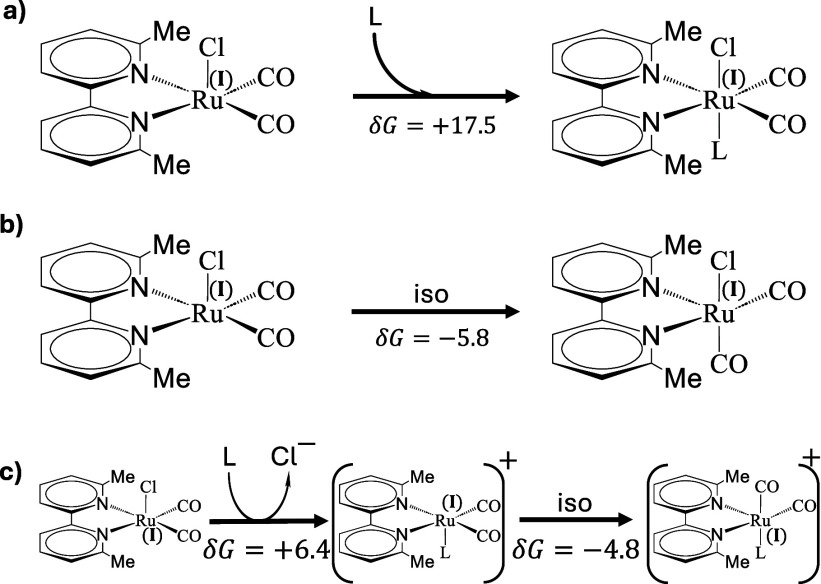
Three considered transformations that could explain the kinetic step occurring with time-constant τ_34_. L = Solvent. The reaction free energies are given in units of kcal/mol.

The last reaction step in the 1 ns to 40 μs time-window with τ_34_ = 5 μs (for Ru6dmb in DMF) blue-shifts the two positive bands back partially to 1943 and 2013 cm^–1^ (**[4]** in Figure 1a). The reaction rate appears too fast for a bimolecular reaction between two species that have already reacted and thus both exist at low concentration (although not completely impossible according to the estimate of the diffusion-limited reaction rate given above, when correcting it for the lower concentrations). Examples of such bimolecular reactions would be further reduction or dimerization of two reduced catalyst molecules. Hence, only three options appear to be conceivable for that reaction step (see Figure 4). These are (Figure 4a) binding of the solvent in the empty coordination site, (Figure 4b) an intramolecular rearrangement where one of the carbonyls would move to the empty axial position, or (Figure 4c) an exchange of the remaining Cl^–^ by a solvent molecule. Even though it would result in a blue-shift of the vibrational frequencies, binding of the solvent to the empty coordination site (Figure 4a) is very unlikely owing to the calculated large energy cost of 17.5 kcal/mol associated with it (see Table S4 in Supporting Information). The 18-electron rule suggests that Ru^I^ remains five-coordinated. Internal rearrangement as the sole process (Figure 4b) is canceled out as well, since the DFT results predict a red-shift for the carbonyl vibrational frequencies (see Table S4 in Supporting Information), as opposed to the observed spectra.

The last choice, ligand exchange (Figure 4c), seems most plausible. It still is energetically uphill according to the DFT results by 6.4 kcal/mol, but much less so than option (a). In addition, the ligand-exchanged species is expected to have blue-shifted carbonyl frequencies, as the loss of the negative charge with Cl^–^ reduces the back-bonding effect. A similar ligand substitution has been previously reported for .^67^ The DFT results suggest that DMF binds via the oxygen atom (Ru–O–C bond).

Once the Ru-complex is 5-coordinated, the ligands are likely to rearrange, moving one of the equatorial carbonyls to the empty axial position, since that reduces the steric strain with the bpy-methyl groups (see second step in Figure 4c). Ligand rearrangement causes a frequency red-shift, which however is smaller than the previous frequency blue-shift due to the chloride-to-solvent exchange. ^1^H NMR spectroscopy discussed later reveals that the final product has broken symmetry, caused by such a ligand rearrangement. We have no TRIR evidence as to when this is happening, but the DFT calculations reveal that the barrier for ligand rearrangement is small (5.8 kcal/mol), in which case one estimates a time-constant of 3 ns according to an Eyring equation, i.e., much faster than ligand exchange. While we cannot rule out that ligand exchange happens after a rearrangement of the chloride species as shown in Figure 4b, the calculated vibrational frequencies agree better with the sequence of events shown in Figure 4c.

In any case, we conclude that the intermediate after the kinetic step occurring with time constant τ_34_ is (iso)- (where “(iso)-” stands for a carbonyl- isomerized species), whose energy is only 1.6 kcal/mol above that of . This energetic cost is paid for by an entropic term accounting for the large concentration of the solvent (12.9 M)
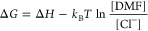
4

Based on the chloride concentration, which originates predominantly from the PS that is supplied as Ru(bpy)_3_Cl_2_, we estimate an entropic stabilization of 3.8 kcal/mol, sufficient to explain that a concerted ligand exchange and rearrangement is indeed likely to happen. We can consider the  intermediate, which is 6.4 kcal/mol higher in energy, to be part of a transition state.

### Hydrogen Atom Transfer

#### TRIR Spectroscopy

**Figure 5:**
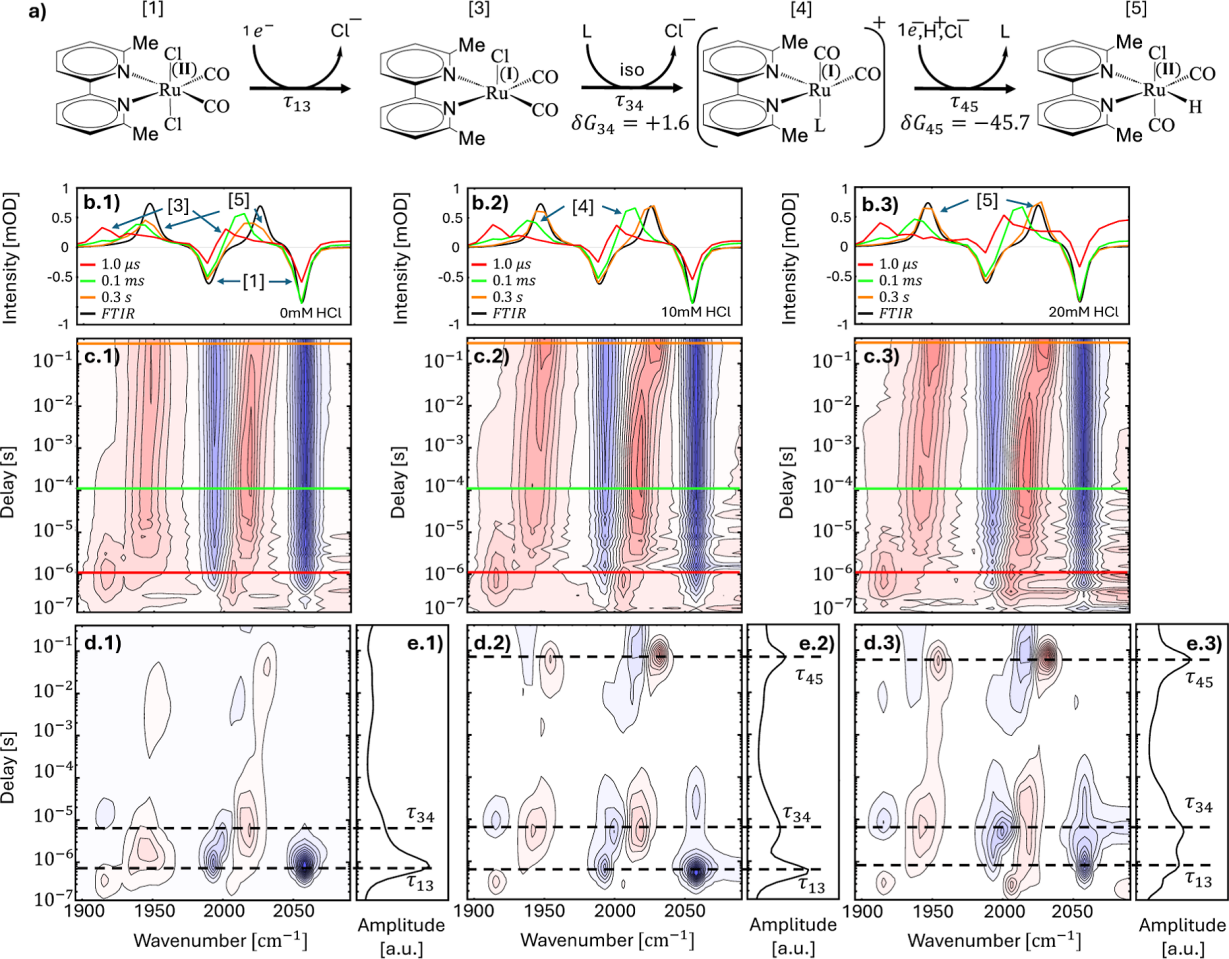
[c(1)] Transient IR spectra of Ru6dmb without addition in the 100 ns to 300 ms time window, as well as after adding [c(2)] 10 mM and [c(3)] 20 mM of HCl. Panels [b(1)–b(3)] show temporal cuts at the selected delay times, together with a steady-state FTIR difference spectrum upon continuous illumination (measured in Bruker Vertex 80v spectrometer). Panels [d(1)–d(3)] show the corresponding lifetime density maps and panels [e(1)–e(3)] the dynamical contents. Panel (a) shows the reaction steps observed in these spectra. The time constants in panel [d(1]) are τ_13_ = 0.7 μs and τ_34_ = 6 μs, while τ_45_ cannot be fit reliably as it is beyond the time-range of these data. The time constants in panel [d(2)] are τ_13_ = 0.6 μs, τ_34_ = 6 μs and τ_45_ = 70 ms, and in panel [d(3)] τ_13_ = 0.9 μs, τ_34_ = 7 μs and τ_45_ = 60 ms. Experimental conditions: 20 mM Ru6dmb, 10 mM Ru(bpy)_3_Cl_2_, 100 mM BNAH in DMF, HCl in increasing amounts, excitation wavelength 447 nm. The reaction free energies are given in units of kcal/mol.

We now turn to the results obtained with the second laser setup, which covers a time-window from 100 ns to 300 ms and hence bridges the gap between time-resolved and steady-state spectroscopies.^71^ The TRIR spectrum of Ru6dmb in DMF in this time window is presented in the Figure 5c(1), accompanied by the spectral cuts (b1), lifetime density map (d1) and dynamical content (e1). The first two reaction steps τ_13_ and τ_34_ are still observed, but the time resolution of the setup (500 ns) is not quite sufficient to reveal reliable values for τ_13_. Here we focus on the long-time, millisecond regime.

Toward the very end of the data in Figure 5c(1), there is an indication of an additional blue-shift of the bands, which is best seen in the temporal cuts of Figure 5b(1) (compare green with orange transient spectrum). The electron density on the metal center is thus further reduced. Figure 5b(1) also shows in black a steady-state FTIR difference spectrum obtained after continuous illumination of the sample at 447 nm, shifted to the blue even further. We conclude that we see the onset of an additional reaction step in the TRIR data of Figure 5c(1), which however is not finished after 300 ms, the longest time we can capture with that laser setup.

We reiterate that the sample does not contain any CO_2_, hence this late reaction step cannot be related to CO_2_ reduction. On the other hand, it has been suggested that protons may play a role in the photoreaction,^7,8^ hence we have added HCl to the original chemical system with concentrations 10 mM and 20 mM (Figure 5c(2),c(3) respectively) to explore the nature of this final reaction step. It is important to emphasize that the addition of HCl deviates the system from conditions in a typical CO_2_ reduction system and is done here only to confirm the proposed reaction mechanism. The last reaction step, which was visible only faintly in the original system, is now fully resolved. In addition, the 300 ms transient difference spectrum perfectly aligns with the steady-state FTIR difference spectrum, indicating that the reaction reached its end with a product that is stable on a minute-to-hour time scale. Furthermore, increasing the concentration of the acid accelerates the last reaction step from τ_45_ = 70 ms at 10 mM to τ_45_ = 60 ms at 20 mM, evidencing that this is a second-order reaction step involving hydrogen-atom transfer and/or chlorination of the catalyst.

#### NMR Spectroscopy

**Figure 6:**
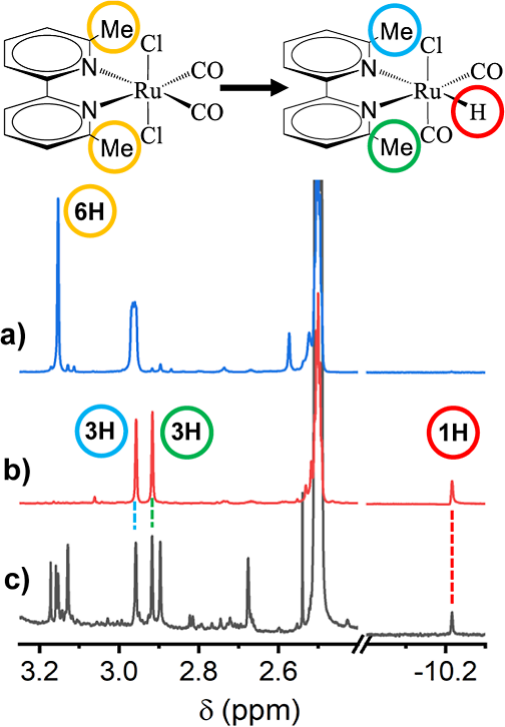
^1^H NMR spectra showing the region of the methyl peaks and the hydride in panel (a) before irradiation, (b) after 10 min of irradiation, and (c) a synthetically produced Ru^II^(6,6′-dmbpy)(CO)_2_ClH. A full version of the spectra can be found in Figure S17 in Supporting Information with further discussion. Experimental conditions: 7 mM Ru6dmb, 3 mM Ru(bpy)_3_Cl_2_, 5 mM BNAH in DMSO-*d*_6_, 400 MHz, excitation wavelength 447 nm.

Given that the final species is stable on a minute-to-hour time scale, ^1^H NMR spectra could be used to identify it (see Figure 6, full versions of the ^1^H NMR spectra, as well as the spectra of individual components, can be found in Section S6 of Supporting Information). ^1^H NMR spectrum were measured before (see Figure 6a) and after continuous illumination of the sample at 447 nm (Figure 6b). In the second case, a singlet is observed at −10.2 ppm, indicating a metal hydride, as such a hydrogen atom would be highly shielded from the applied magnetic field and appear upfield with respect to the Lamor frequency of the TMS reference proton.^72^ In addition, the sharp peaks in the ^1^H NMR spectrum of the photoproduct evidence that there are no paramagnetic species present. Existing literature reports similar hydride peaks at −11.3 ppm for Ru(bpy)(CO)_2_(Cl)H,^54^ and −11.7 ppm for Ru(6,6′-dimesityl-bpy)(CO)_2_(Cl)H,^39^ both in CD_2_Cl_2_.

For a one-to-one comparison with the photoproduct, Ru^II^(6,6′-dmbpy)(CO)_2_ClH was synthesized, see Supporting Information, Section S1. Although the yield was low and we could not separate the compound from the rest of the reaction products, it was still sufficient to measure a ^1^H NMR spectrum (Figure 6c). The chemical shift of the synthesized hydride is precisely the same as that of the photochemically produced one.

The NMR results also indicate that the final complex has broken symmetry without any mirror plane between the two pyridinium parts, as evidenced by the methyls’ singlet before the irradiation that splits into two bands after the light-induced reaction (see Figure 6a,b). Each of the product peaks show an integral of 3 when the hydride peak is normalized to 1. In the NMR spectrum of the nonirradiated sample, a hydrogen on the bpy ring was used for the integral normalization.

From all possible symmetry-broken isomers of Ru^II^(6,6′-dmbpy)(CO)_2_ClH, the one shown as compound **[5]** in Figure 5a has the lowest energy, since the hydride in an equatorial position reduces the steric strain with the bpy-methyl groups. Furthermore, two carbonyls in axial positions would not be compatible with the experimental IR spectrum, since the antisymmetric stretch vibration would then be essentially dark. We hence conclude that the final product is compound **[5]** shown in Figure 5a.

#### Fate of the Oxidized BNAH^•+^

In Figure 5c(2),c(3), we have added protons to the sample, but in Figure 5c(1) we have not. Furthermore, the diamagnetic character of the sample evidence that not only a proton is transferred to (iso)-, but also an electron. In the following, we will argue that the source of both the proton and the electron is the oxidized BNAH^•+^.

Possible decay pathways of the oxidized BNAH^•+^ are well-known.^52^ It is highly acidic with a p*K*_a_ < 1,^73^ hence it is likely to donate a proton in the presence of a base. BNA^•^ can then either dimerize into BNA_2_,^22^ or donate a second electron in a dark process, in which case it produces a stable pyridinium species (BNA^+^). If BNA_2_ formation is favored over the BNA^+^, then BNAH is a one-electron donor. The latter has been observed in a lot of studies where chemical system contains a base (e.g., TEOA) and/or significant concentration of water.^37,74−78^ In our studies, there is no base added to the system and the singly reduced catalyst serves this purpose instead. Indeed, we see no evidence of BNA_2_ formation in the NMR results of the irradiated chemical system (see Supporting Information, Figure S17b), and hence conclude that it is a two-electron source. Since BNAH^•+^ exists in the sample at the same concentration as (iso)-, there are sufficient electrons to reduce the latter twice.

#### Re-Chlorination

Based on the NMR results, we concluded that the final product is Ru^II^(6,6′-dmbpy)(CO)_2_ClH, with chloride as a ligand, while intermediate **[4]** has a solvent molecule as a ligand: (iso)-. Additional evidence for the fact that the final product no longer ligates a solvent molecule is provided from a comparison of stationary FTIR difference spectra in DMF versus the nonligating 1,2-dichloroethane (DCE, see Section S5 and Figure S10 in Supporting Information). The calculated binding energies indicate that the exchange of Cl^–^ by the solvent is thermodynamically unfavorable process, as has been stated earlier. The energy cost of exchange is significantly lower for Ru^I^ (∼6.4 kcal/mol) than it is for Ru^II^ (∼16.1 kcal/mol). In  it is just possible due to subsequent ligand rearrangement and the counter-acting entropic driving force (eq 4). But the final hydride species is formally a Ru^II^ species, which shifts the equilibrium back to the Cl^–^ ligand, just like it is in the resting state of the catalyst. Even when reducing the Cl^–^ concentration as much as possible by using Ru(bpy)_3_(PF_6_)_2_ as PS instead of Ru(bpy)_3_Cl_2_, rechlorination still occurs, see Figure S10c.

Summing up the bits and pieces of what has been discussed so far, we conclude that three processes must happen during the last reaction step occurring with time-constant τ_45_: proton transfer, electron transfer and rechlorination. It is hard to be definite about the order of these processes, however it is likely that the back-exchange of the coordinated solvent to Cl^–^ happens after hydride formation, when the oxidation state of the metal center changes back to Ru^II^. Upon addition of HCl, the last reaction step τ_45_ accelerates. A reasonable explanation for this trend is that hydrogenation and rechlorination become near-simultaneous as the concentration of the ingredients for both of the processes is increased. On the other hand, proton and electron transfer are sequential, since the source of the electron is BNAH^•+^ in any case, whose concentration was not changed in the measurement series of Figure 5.

The DFT results would predict three vibrational modes in the carbonyl region for the final product, the two −C≡O stretches and a hydride stretch, see Table S4. With the used DFT method, the Ru–H vibration becomes accidently degenerate with one of the CO modes and therefore gains oscillator strength. We however see only two of the three predicted modes in the experimental spectra (Figure 5). To address this issue, we compare in Figure S10b FTIR difference spectra of the original system with one where an excess of D_2_O has been added, in which case the final product must be the deuterated hydride. Both difference spectra are in essence the same. We therefore conclude that the intensity of the original Ru–H vibration is negligible, since the predicted degeneracy is an artifact of the particular DFT method. Metal-hydride vibrations are in fact very elusive and it is well-known that DFT has severe problems in correctly predicting their frequencies and intensities.^79^

DMF is a highly hygroscopic solvent, and NMR measurements indicate that some amount of water may be present during the reaction. We concluded above that the last chemical species undergoes proton–electron transfer, followed by the incorporation of a chloride ion. This suggests that water could play a role in this process by providing protons. To test this hypothesis, we varied the water concentration from 0 to 50 mM, see Figure S8 in Supporting Information. From the lifetime analysis [Figure S8c(1)–c(4)], we conclude that there is no notable shift in the time scales of the observable reaction steps nor the appearance of any new set of bands. In particular, the reaction step equivalent to τ_45_ is not observed. Water thus is not acidic enough to support the observed hydrogen atom transfer. Indeed, this fact is supporting the hypothesis that oxidized BNA^•+^ provides a second electron coupled with a proton transfer.

**Figure 7:**
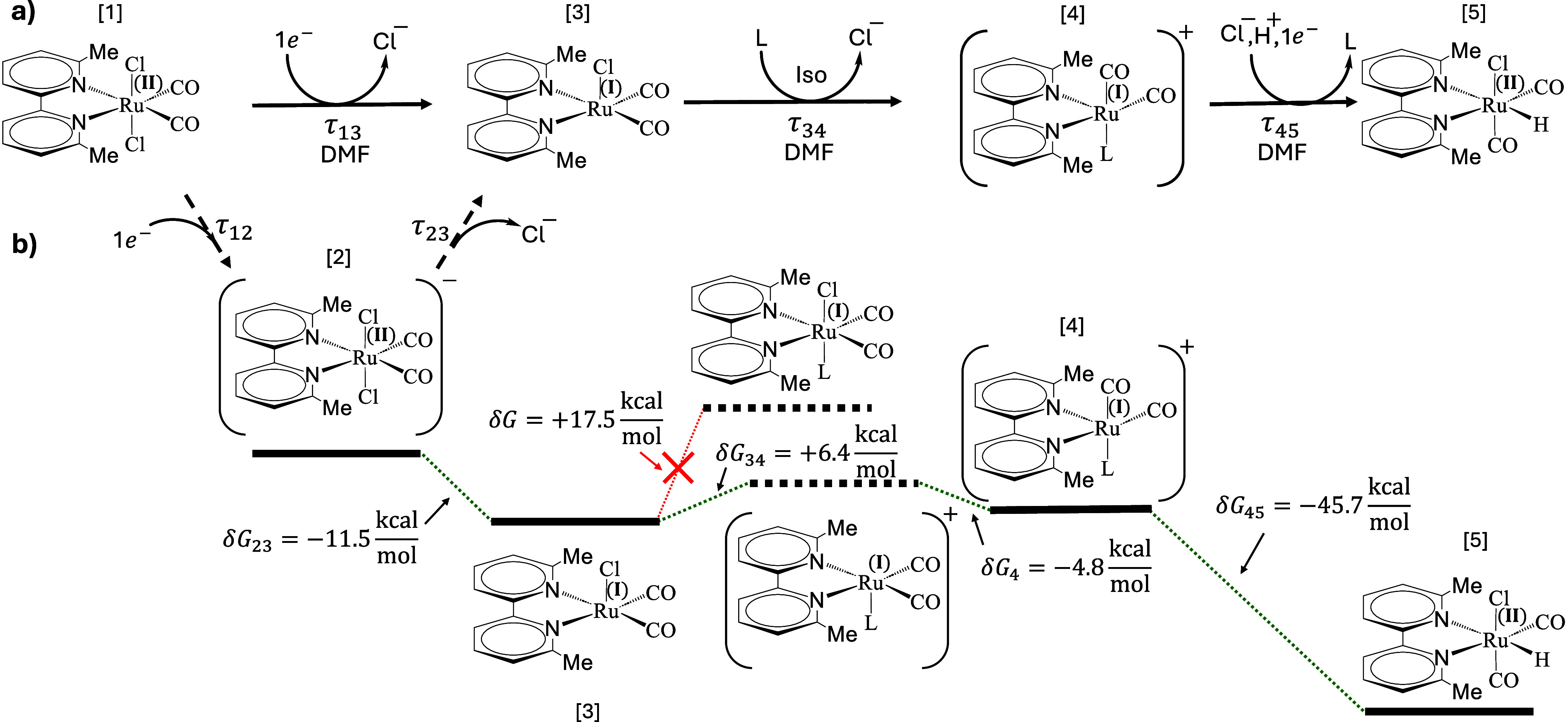
Reaction pathway (a) and energetics (b) of the complete reaction, as deduced from transient IR spectroscopy, NMR spectroscopy, as well as DFT calculations.

To conclude this chapter, Figure 7 summarizes the reaction pathway and the energetics of the various intermediates, starting from the resting state of the catalyst, Ru^II^(6,6′-dmbpy)(CO)_2_Cl_2_ until the final product, Ru^II^(6,6′-dmbpy)(CO)_2_ClH.

### Complications

**Figure 8:**
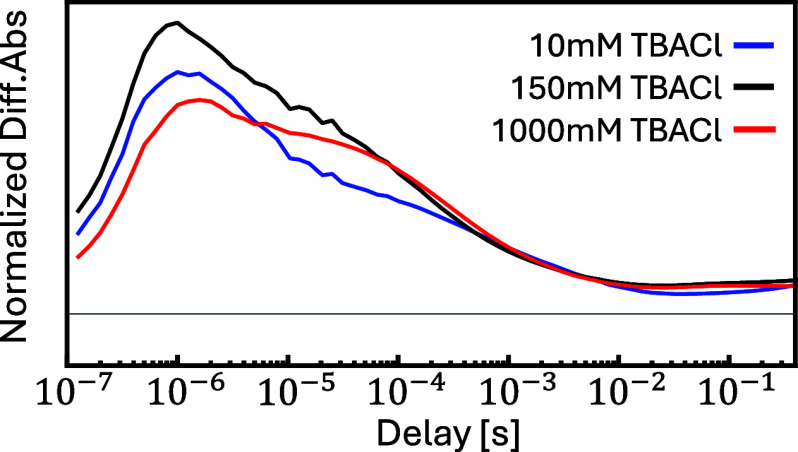
Chloride concentration dependence. Shown are temporal cuts at probe frequency 1917 cm^–1^ extracted from the data in Figure S9 at 10 mM (blue), 150 mM (black) and 1 M (red) TBACl concentration. The data are normalized according to the bleach of Ru^II^(6,6′-dmbpy)(CO)_2_Cl_2_]. Experimental conditions: 20 mM Ru6dmb, 10 mM Ru(bpy)_3_(PF_6_)_2_, 100 mM BNAH in DMF, TBACl in increasing amounts, excitation wavelength 447 nm.

Figure 7 shows the essence of the reaction pathway, but the reaction is more complicated in detail. For example, all reaction steps are chemical equilibria with a forward and possibly also a backward rate. We observe that explicitly for the first ligand exchange reaction τ_34_, the equilibrium constant of which should depend on the Cl^–^ concentration according to eq 4. To that end, Figure S9 in the Supporting Information shows a series of experiment with the Cl^–^ concentration varied from 0 mM to 1 M (added in the form of tetrabutylammonium chloride, TBACl). Figure 8 highlights the appearance and disappearance of the vibrational band characteristic for Ru^I^(6,6′-dmbpy)(CO)_2_Cl]^•^ via temporal cuts at probe frequency 1917 cm^–1^, which has been extracted from Figure S9. The decay of that band is actually biphasic, with a second hump at about 100 μs that is most pronounced at 1 M Cl^–^ concentration (Figure 8, red line). At low 10 mM concentration (Figure 8, blue line), the bigger fraction (not all) of the signal decays with τ_34_ = 6 μs. In accordance with eq 4, ligand exchange is thus not complete and less likely at higher Cl^–^ concentration. In addition, the maximum intensity of the Ru^I^(6,6′-dmbpy)(CO)_2_Cl]^•^ band also depends on Cl^–^ concentration, peaking at 150 mM (Figure 8, black line). We assume that this reflects the fact that also the loss of the first chloride (τ_12_), which is not resolved in the case of Ru6dmb, is affected by the Cl^–^ concentration and might compete with back-electron transfer.

**Figure 9:**
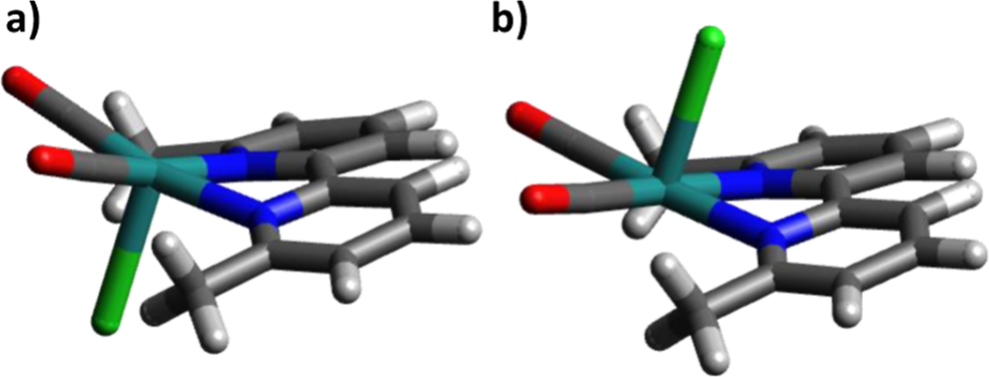
Calculated structures of the two conformers of : the chloride aligned with the methyl groups (awm, panel a) and aligned against them (aam, panel b).

Another complication concerns the nonexponential kinetics of τ_13_ and τ_34_, despite the fact that they should be first-order reactions. That is, the first electron transfer step is quasi-first order, since the concentration of the reduced PS (1 mM, as determined from the laser pulse energy) is much lower than that of the catalyst (20 mM). The same applies to ligand exchange, with the concentration of the solvent being much larger than that of the reduced catalyst. Finally, ligand rearrangement is unimolecular in nature. However, attempts to fit reaction steps τ_13_ and τ_34_ by global exponential fitting failed, which is why we applied here to the less biased dynamical content analysis. For example, τ_13_ and τ_34_ in Figure 5e(3) merge into a single, but very broad peak. We attribute these complications to two effects. First, τ_34_ likely includes two events, ligand exchange and ligand rearrangement, which are too close in time to separate them in most cases, but they still might cause heterogeneity in the kinetics. Second, Ru6dmb is not planar due to steric strain between the bpy-methyl groups and the Ru-carbonyls, resulting in different conformers, see Figure 9. These conformers differ in energy by a sizable amount (∼1.6 kcal/mol, see Table S4 in Supporting Information), which might also affect the kinetics. Spectroscopically, they are however very similar and we cannot resolve them. The kinetic step occurring with τ_45_ is intrinsically already a second-order process, and hence not exponential. Moreover, the effects described above, i.e. more than one event at essentially the same time, and the involvement of energetically different conformers, add up to even more complicated kinetics, and a detailed (model based) fit will not work either.

In addition, a kinetic process at around 5 ms can be identified. It is most evident in the experiment with reduced chloride concentration, see Figures S9c(1),d(1) in Supporting Information, where it is denoted as τ*, but hints of that process are visible also in all other data. The peak is associated with a slight blue-shift of both CO stretching vibrations and a change in intensities. That is, the higher frequency band is more intense before that process, shifting to the lower frequency band afterward. While an assignment of that feature will remain speculative, we suggest that it is related to the formation of an encounter complex between the reduced Ru6dmb catalyst and the oxidized BNAH^•+^ radical. The latter will draw some of the charge from the reduced Ru6dmb catalyst, hence the blue-shift of the CO stretching vibrations. That encounter complex might be a preparative step for the final reaction step τ_45_ discussed above. The feature is most distinct at low chloride concentration, where the further steps involving hydrogen atom transfer and rechlorination are well separated in time.

### Dimerization/Disproportionation in Ru5dmb

**Figure 10:**
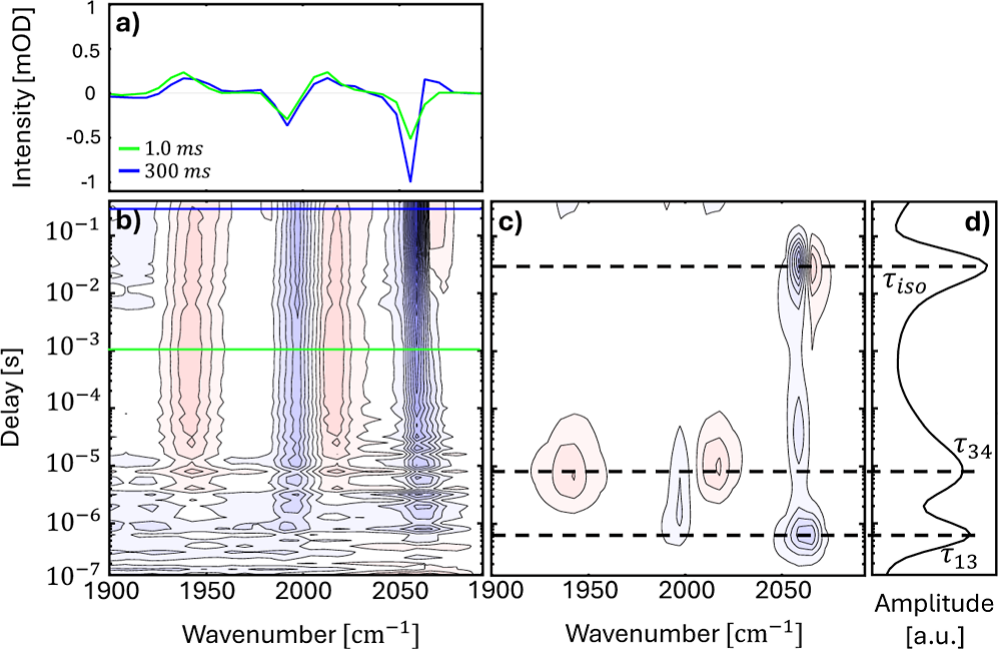
(b) Transient IR spectra of Ru5dmb. Panel (a) shows spectral cuts at selected delay times, panel (c) the lifetime density map and panel (d) the dynamical content. The time constants are τ_13_ = 0.6 μs, τ_34_ = 8 μs and τ_*iso*_ = 30 ms. Experimental conditions: 20 mM Ru5dmb, 10 mM Ru(bpy)_3_Cl_2_, 100 mM BNAH, 10 mM HCl in DMF, excitation wavelength 447 nm.

The reaction pathway discussed above for Ru6dmb is different from the one of Ru5dmb (and probably also different from Ru4dmb). Although the first two processes, i.e., initial reduction and subsequent Cl^–^ loss remain essentially unchanged (see Figure 2), there is no steric factors in Ru5dmb that would drive the carbonyl ligand’s rearrangement after the first Cl^–^ loss, hence the next process presumably is an exchange of the remaining Cl^–^ by a solvent molecule. Furthermore, as can be seen from Figure 10b, a new feature appears at ≈30 ms, labeled as τ_iso_. There is a further bleaching step accompanied by a new band shifted toward higher frequencies. From the high frequency of this band, it is clear that it must originate from a Ru^II^-species.

**Figure 11:**
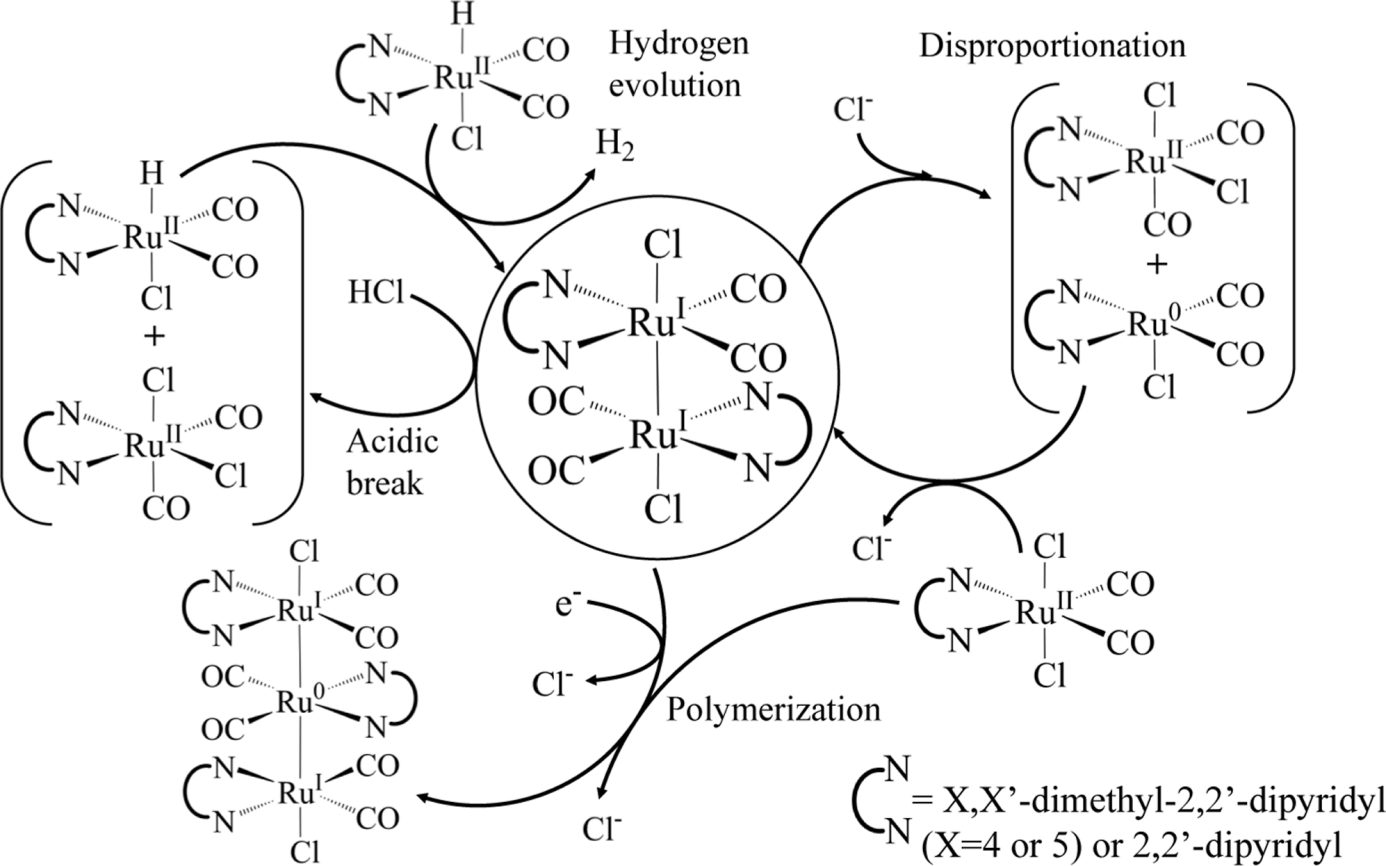
Network of possible mechanisms involving the Ru^I^–Ru^I^ dimer, as suggested in literature.^38,74,80−82^

It is known that Ru5dmb (and also Ru4dmb) can polymerize after reduction.^38^ It has furthermore been reported that the dimer can participate in several reaction pathways,^80,82^ summarized in Figure 11, including a disproportionation reaction in the presence of Cl^–^ into Ru(0) and Ru(II) species, the latter of which forms *cis*(Cl)-[Ru^II^(5,5′-dmbpy)(CO)_2_Cl_2_].^81^ The Ru(0) species can again reduce the initial Ru5dmb to form a dimer, which explains the secondary bleach at the later time delays. On the other hand, the symmetric CO stretching mode of the *cis*(Cl)-[Ru^II^(5,5′-dmbpy)(CO)_2_Cl_2_] isomer absorbs at a higher frequency than that of the *trans*(Cl)-[Ru^II^(5,5′-dmbpy)(CO)_2_Cl_2_] isomer, see DFT results in Tables 1, S4 as well as Figure S4 in Supporting Information, which compares the FTIR spectrum of the synthetically produced *cis*(Cl)-[Ru^II^(5,5′-dmbpy)(CO)_2_Cl_2_] isomer to that of the starting molecule.

**Figure 12:**
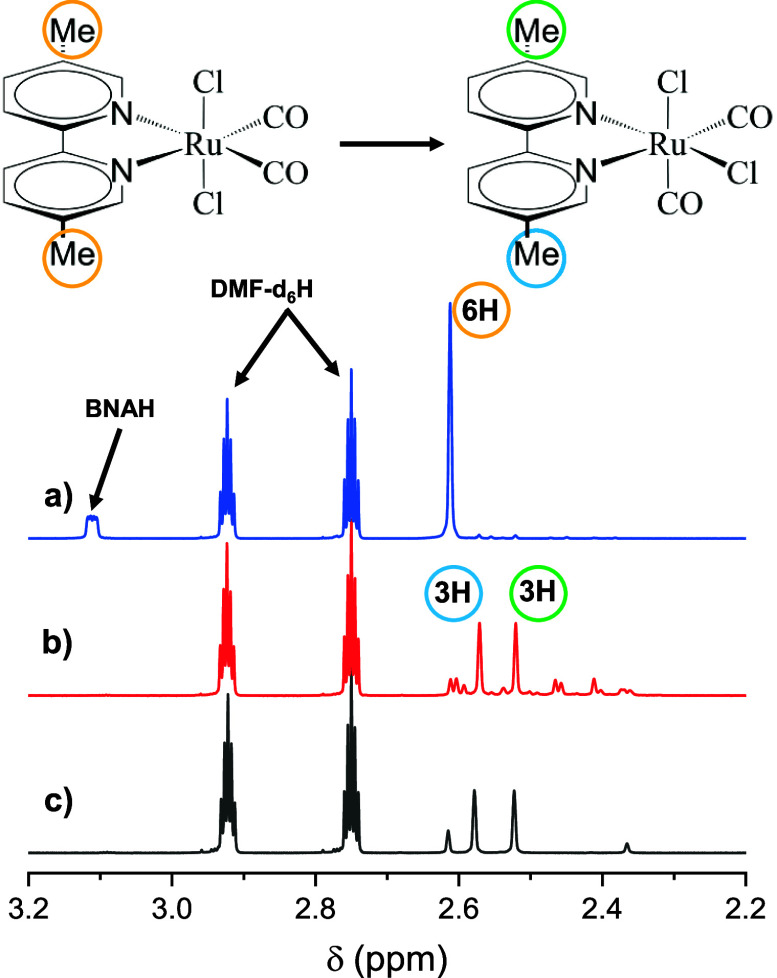
^1^H NMR spectrum of the irradiated chemical system in the 2.2–3.2 ppm region of Ru5dmb (a) before irradiation, (b) after 3 min of irradiation, and (c) for synthetically produced *cis*(Cl)-[Ru^II^(5,5′-dmbpy)(CO)_2_Cl_2_]. A full version of the spectra can be found in Figure S18 in Supporting Information Experimental conditions: 10 mM Ru5dmb, 10 mM Ru(bpy)_3_Cl_2_, 10 mM BNAH in DMF-*d*_7_, 400 MHz, excitation wavelength 447 nm.

NMR irradiation experiments provide compelling evidence to support this assignment. Figure 12 presents the ^1^H NMR spectrum of a solution with PS, Ru5dmb and BNAH in DMF-*d*_7_ (a) before and (b) after irradiation for 3 min with a 447 nm laser. Before illumination, the two methyl groups in Ru5dmb appear as a single band since the molecule possesses C_2*v*_ point group symmetry (Figure 12a). After irradiation however (Figure 12b), we see a large number of peaks related to the methyl protons. Two bands stand out, both labeled as 3H. They have a 1:1 ratio when integrated, suggesting they belong to the same molecule, but one which has lost its symmetry. The latter is a clear support of the formation of the *cis*(Cl)-[Ru^II^(5,5′-dmbpy)(CO)_2_Cl_2_] isomer as described above, and is confirmed by the comparison of the two peak positions with those observed for synthesized *cis*(Cl)-[Ru^II^(5,5′-dmbpy)(CO)_2_Cl_2_] (Figure 12c). The smaller peaks that form during the photoinduced reaction must be related to polymerization products of reduced Ru5dmb. The dimer, as well as higher order polymers, may exist in a number of conformations (rotamers), which precludes the possibility to assign these peaks individually. The same is even more true in the respective TRIR spectra, where we can not identify any additional bands besides that from *cis*(Cl)-[Ru^II^(5,5-dmbpy)(CO)_2_Cl_2_] isomer, presumably because they are too weak and smeared out.

Moreover, the acidic proton can in fact play a role in the formation of the dimer, albeit indirectly, see Figure 11, left side. As we have seen in the case of Ru6dmb, a metal hydride can be produced by proton and second electron transfer from oxidized BNAH to the singly reduced catalyst. The same process could also take place in the chemical system containing Ru5dmb. That said, the Ru^II^(5,5′-dmbpy)(CO)_2_ClH species with the hydride in the axial position, can form a Ru^I^–Ru^I^ dimer that results in H_2_ evolution.^80^ As a matter of fact, the ^1^H NMR spectrum of the irradiated system (see Supporting Information, Figure S17d) shows a very small fraction of the hydride species. The two peaks at −10.7 and −10.2 ppm can be assigned to Ru^II^(5,5′-dmbpy)(CO)_2_ClH^(ax.)^ and Ru^II^(5,5′-dmbpy)(CO)_2_ClH^(equat.)^, respectively. The reduced catalyst dimer complex, , or its singly solvated form - [(Ru^I^(5,5′-dmbpy)(CO)_2_)_2_(Cl)(DMF)]^+^ - has 2 additional electrons that can be a target for a H^+^ attack. Coupled with a chloride in solution, such a reaction can yield a Ru^II^–H species and an parent Ru5dmb or its *cis*(Cl) isomer. The hydride would reform another dimer by reacting with another hydride.

## Conclusions

We have tracked down the photochemical reduction of *trans*(Cl)-[Ru(6,6′-dimethyl-2,2′-bipyridine)(CO)_2_Cl_2_]. Figure 7 provides an overview of the reaction. The mechanism starts with the loss of a single Cl^–^ ligand upon the one-electron reduction of the catalyst by the reduced PS, PS^•–^. In case of Ru6dmb, the loss of the Cl^–^ is much faster than the kinetics of the electron transfer to the catalyst, hence the first transient intermediate that must occur - Ru^II^(6,6′-dmbpy^•–^)(CO)_2_Cl_2_ - is not observed. On the other hand, we do observe that intermediate for Ru4dmb and Ru5dmb due to the better stabilization of the electron in the planar π-system and a better accessibility for the PS. In either case, the remaining Cl^–^ is then replaced by a solvent molecule. The energetic cost of this process is paid for by the large excess concentration of the solvent with respect to free Cl^–^, as well as a rearrangement of the ligands to relieve the steric strain in Ru6dmb. These steps are diffusion controlled among species at relatively high concentrations (10 mM or larger), and occur on a tens of nanoseconds to microsecond time scale.

At least in the presence of the oxidized electron donor, (iso)- is not a stable intermediate. Rather, the catalyst gets protonated, accompanied by a second electron transfer from BNAH^•+^. Furthermore, the resulting negatively charged hydride causes a formal reoxidization of the metal center to Ru^II^. The ligand equilibrium is only on the solvent side when the oxidation state of the metal center is Ru^I^ and reverts back to Cl^–^ in the Ru^II^ species. Hence, hydrogen atom transfer leads to rebinding of Cl^–^, revealing the final product, Ru^II^(6,6′-dmbpy)(CO)_2_ClH, which in fact is stable on a minute-to-hour time scale. The latter agrees with the mechanism proposed by Kubiak and co-workers^39^ for a similar catalytic complex. Three processes must happen during the last reaction step in a (semi)-concerted manner, proton transfer, electron transfer and rechlorination, with all three components at low concentrations, which renders the process slow, tens to hundreds of milliseconds.

It has been argued by Kuramochi et al.^38^ that dimerization of Ru6dmb is not possible due to the strong deformation of its bipyridine ligand. Here, we refine this argument somewhat. That is, carbonyl rearrangement releases the strain that causes the deformation of the bipyridine ligand once the complex is only 5-coordinated. The sites that would be required for a Ru–Ru bond in a dimer are then blocked.

The molecular system we studied here is not reactive, i.e., does not reduce CO_2_, to start with since we did not add any CO_2_ to the sample. It is reasonably established that these catalysts require a two-step reduction before they become active,^74^ and the purpose of this study has been to explore that process. We deliberately chose to focus on Ru6dmb, despite the fact it is not the best CO_2_ reduction catalyst, since it was expected to be the simplest in terms of its reaction cycle, given that dimerization is prevented by the two methyl groups. In contrast to this expectation, the results reveal that the reaction cycle is still remarkably complex. We feel that it is important to set the stage and to fully understand the catalyst alone, before adding CO_2_, which will interfere with the reaction cycle established here at some stage. In fact, the final product, Ru^II^(6,6′-dmbpy)(CO)_2_ClH, stores two electrons due to the capability of the BNAH^•+^ to deliver a second electron, albeit on a rather slow time scale. Given that the final product is stable for minutes to hours, it is probably not a very active catalyst. It has been suggested that CO_2_ addition and hydride formation are competing pathways for this type of catalysts, the latter leading to formate formation and/or H_2_ evolution.^35,36^ These reaction pathways however also require dimerization of the catalyst,^74^ which we have shown is not possible for Ru6dmb. It has also been suggested that CO_2_ can in fact attack the hydride after it is formed.^39^ It is currently not clear whether the hydride is a dead-end for CO_2_ reduction, or, on-pathway. In the latter case, one would assume that formate should be a reaction product, which however has not been observed according to Kuramochi et al.,^38^ which would favor the hypothesis of the hydride being a dead-end. On the other hand, Machan et al.^39^ have proposed a reaction mechanism, in which a hydride intermediate does not necessarily lead to formate as a product. The addition of CO_2_ in future experiments will clarify these questions. We expect that CO_2_ will kinetically compete with hydride formation from the oxidized electron donor BNAH^•+^, depending on the CO_2_ concentration, and in that way control the splitting ratio between different pathways. The present study provides solid cornerstones for the kinetic control of such catalytic systems. We established the time window, during which the probably active species (iso)- is present (100 ms to seconds, see Figure 5), setting an upper limit for CO_2_ to react with it.

Besides elucidating the first two reduction steps of a prominent CO_2_ reduction catalyst in unprecedented detail, this study also illustrates how TRIR spectroscopy, which so far is not widely used in electrochemical studies, can complement spectroelectrochemistry. By being able to resolve the sequence of appearance of various intermediate species during a reaction cycle, it is much easier to assign them, as only one or few properties of the catalytic molecule change during each reaction step. The discrepancy between the equivalent reduction potentials of Ru4dmb, Ru5dmb and Ru6dmb^38^ versus the kinetics of the first reaction step τ_12_, which is slower by a factor 10 for Ru6dmb as compared to Ru4dmb and Ru5dmb, is a prime example for the advantage of time-resolved experiments over CV or spectroelectrochemistry. In that regard, it is important to cover all relevant time scales, from picoseconds (see e.g. Figure S6) to potentially seconds (Figure 5), in order not to miss any intermediate, which could easily lead to misinterpretations.

It would be advantageous if only one property changed per reaction step, but we have seen here that this is not necessarily the case. When a particular intermediate is populated with a time constant slower than it is depopulated only one reaction step is experimentally observable and the particular intermediate cannot be seen. Even though the catalyst Ru6dmb, which is the focus of the present study, was selected to be as simple as possible, five kinetic steps could be identified (τ_12_ through τ_45_, as well as τ*), that comprise in total eight elementary reaction steps (i.e., reduction, chloride loss, ligand exchange, ligand rearrangement, formation of an encounter complex, proton as well as electron transfer and another ligand exchange). In a typical spectroelectrochemical experiment, some of these intermediates would be seen all at once, others not at all, because the technique lacks time resolution. The kinetic steps cover a wide range of time scales ranging from nanoseconds (and potentially even faster) to hundreds of milliseconds. Hence, the TRIR spectrometer must be able to capture such a wide range of time scales, which became possible with recent technological developments.^64^ It is expected that TRIR spectroscopy will become increasingly more important in catalytic and electrochemical investigations.

## Data Availability

Raw data have been deposited in Zenodo (https://doi.org/10.5281/zenodo.14779819).

## Supporting Information Available

Experimental Methods (Section S1 and S2), results on the competing photodegradation of the catalyst (Section S3), TRIR control experiments (Section S4), supporting FTIR and NMR spectra (Section S5 and S6), as well as methods and results for the DFT calculations (Section S7) (PDF)
