# Supplementary material for: Unveiling the Activation Pathway of the CO2 Reduction Catalyst trans(Cl)-[Ru(X,X′-dimethyl-2,2′-bipyridine)(CO)2Cl2] by Direct Spectroscopic Observation
Source: ACS Catal. 2025 Feb 5;15(4):3023–37. doi: 10.1021/acscatal.4c06974 (PMC11851782; doi:10.1021/acscatal.4c06974)
Supplement: Supplementary file 1 — cs4c06974_si_001.pdf [file cs4c06974_si_001.pdf]

Supporting Information: Unveiling the Activation Pathway of the CO_2 reduction catalyst *trans*(Cl)-[Ru(X,X'-dimethyl-2,2'-bipyridine)$(CO)_2Cl_2$] by Direct Spectroscopic Observation

Sergio Aranda-Ruiz,[†,‡] Luka Tatarashvili,[†,‡] Kerstin Oppelt,[†] and Peter Hamm[*,†]

†*Department of Chemistry, University of Zürich, Zürich, Switzerland*

‡*Both authors contributed equally to this work*

E-mail: peter.hamm@chem.uzh.ch

1 Synthesis

All chemicals and solvents used during the experiment, as well as the chemical precursors needed for the synthesis of the different catalysts, were commercial reagent quality (see Table S1).

Fig. S1 summarizes the syntheses performed in the course of this study. Starting from $RuCl_3{\cdot}xH_2O$ as common precursor for all investigated Ru-bpy compounds one can either proceed via a Ruthenium carbonyl polymer $[Ru(CO)_2Cl_2]_n$ (Fig. S1a), which would favour the formation of *trans*(Cl)-RuXdmb, or via the dimer $[Ru^{II}(CO)_3Cl_2]_2$ (Fig. S1c), which preferentially produces *cis*(Cl)-RuXdmb in our experience. Previous studies report that these starting materials are interchangeable,[1–4] but we found that this causes impurities

Table S1: Chemicals specifications

Chemical	Purity [%]	Brand	CAS-n°
6,6'-dimethyl-bipyridine	98.0	Sigma Aldrich	4411-80-7
5,5'-dimethyl-bipyridine	98.0	Sigma Aldrich	1762-34-1
4,4'-dimethyl-bipyridine	99.5	Sigma Aldrich	1134-35-6
$[Ru(bpy)_3]Cl_2$	97.0	Combi Blocks	14323-06-9
$[Ru(bpy)_3]Cl_2 \cdot 6H_2O$	-	Sigma Aldrich	50525-27-4
$[Ru(bpy)_3](PF_6)_2$	95	abcr	60804-74-2
$RuCl_3 \cdot xH_2O$	97.0	fluorochem	14898-67-0
BNAH	$\geq$ 95.0	TCI	952-92-1
BNA^+		Merck	5096-13-9
Paraformaldehyde	95.0	Sigma Aldrich	30525-89-4
DMF	98.0	Thermo Scientific	68-12-2
DMSO	99.9	Thermo Scientific	67-68-5
HCl	32.0	Merck	7647-01-0
Formic acid	98	SCHARLAU	64-18-6
CH2Cl2	100.0	Sigma Aldrich	75-09-2
$[Ru(CO)_3Cl_2]_2$	98.0	abcr	22594-69-0

coming from the corresponding isomer, which would require extra purification steps.

The left branch of the scheme describes how to obtain the three different forms of the catalyst *trans*(Cl)-Ru(X,X'-dmbpy)$(CO)_2(Cl)_2$ (abbreviated as RuXdmb, Fig. S1c), which are the central focus of this investigation. The right branch describes a set of compounds which were crucial to clarify the experimental results. Since *cis*(Cl)-Ru(X,X'-dmbpy)$(CO)_2(Cl)_2$ (abbreviated as cis-RuXdmb, Fig. S1d) might have been a transient intermediate in the photo-reaction, it was synthesized to compare its spectroscopic signature. Similarly, we attempted to synthesize the Ru(I)-Ru(I) dimer, Fig. S1f. In case of Ru4dmb and Ru5dmb, dimerization has been suggested to occur during the photo-reaction. This is not the case for Ru6dmb due to the deformation of its bipyridine ligand.[5] Our unsuccessful attempts to synthesize the Ru(I)6dmb-dimer (see Fig. S1), using the same protocol as for Ru4dmb and Ru5dmb[3] provide evidence that it is thermodynamically very unfavorable. Finally, the most relevant compound shown in Fig. S1e is the final product in the photochemical reaction, and we synthesized it for comparison of FTIR and NMR spectra.

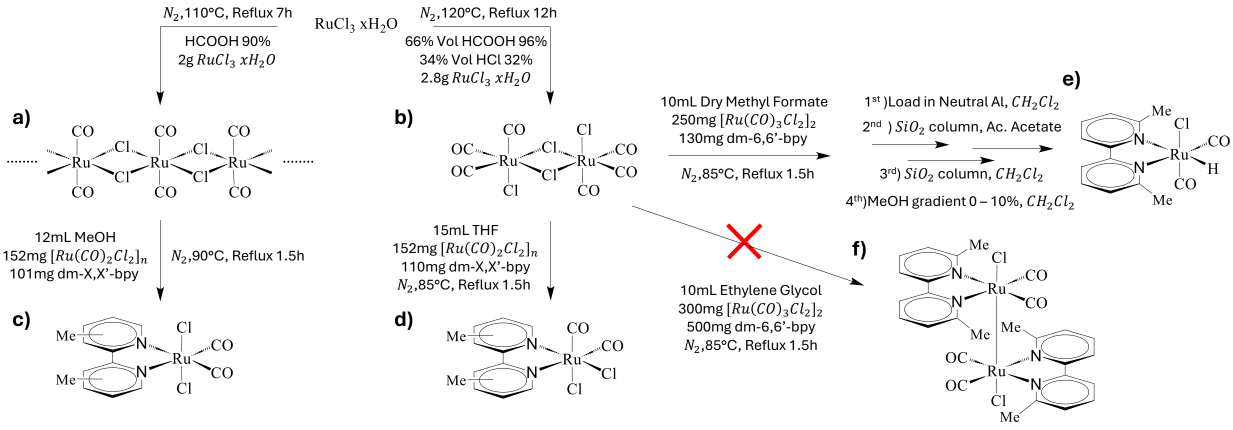

Figure S1: Summary of the different syntheses performed for this study.

Details of the syntheses of the various compounds are described in the following:

1.1 Synthesis of $[Ru^{II}(CO)_2Cl_2]_n$ (Fig. S1a)

We followed the protocol of Ref.[6] The reaction was carried out in 50 ml of formic acid 96 % in a 100 ml two-necked round-bottomed flask. The solvent was purged with N_2 for 5 min, then 2.0 g of $RuCl_3\cdot xH_2O$ and 1.0 g of paraformaldehyde were added. The solution was kept under reflux conditions at 110°C for 24 h, with the temperature controlled by a thermocouple submerged in the silicon oil bath. During the reaction time, the color changed from orange to dark blue within an hour, then in turned greenish after another 30 mins, and finally, after 5-7 h, the solution turned yellow.

When the reaction is complete, the mixture is cooled to room temperature, and transferred to a round-bottomed flask. The solvent is removed at 60 mbar and 53 °C using a rotavapor. The resulting solid is transferred to a vial, where it is dried under vacuum for 3 h at 3 mbar. The remaining solid (dark orange) is washed and triturated with n-hexane. Subsequently, it is isolated using a Büchner funnel with a filter paper of MN 615 variety. Once again, the compound is dried under vacuum for 1 h at 3 mbar.

The obtained product was 1.65 g of $[Ru(CO)_2Cl_2]_n$, but since the hydration degree is unclear for $RuCl_3$, the final yield could be imprecise. Assuming $RuCl_3\cdot 3H_2O$, the estimated

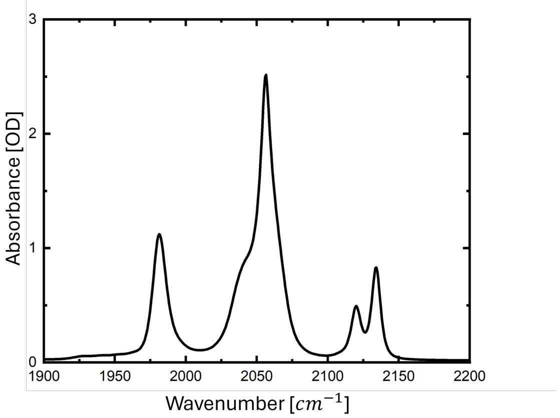

Figure S2: FTIR spectrum of the $[Ru(CO)_2Cl_2]_n$ polymer precursor in DMF

reaction yield is 75 %. The final IR signature is shown in Fig. S2. One possible impurity is $[Ru(CO)_3Cl_2]_2$,[7] which would have an effect in the synthesis of the final catalyst, leading to the formation of cis-Ru6dmb.

IR (DMF, cm^{-1}): 1980.6 (s,CO), 2056.4 (s,CO), 2120.1 (s,CO), 2134.3 (s,CO)

1.2 Synthesis of $[Ru^{II}(CO)_3Cl_2]_2$ (Fig. S1b)

We followed the protocol of Ref.[7] 25 ml of formic acid 96 % in a 2-necked round-bottom flask was bubbled with with N_2 through a glass needle for 10 min to decrease the presence of O_2. The next reactant added was HCl 32 % (18 ml). In order to not evaporate it, the solution was no longer bubbled, rather the blanket N_2 flow from the top of the condenser kept the solution oxygen free. Finally, 2.0 g of $RuCl_3 \cdot xH_2O$ were added to the flask. The solution was refluxed at 120 °C, during 12 to 18 h. Subsequently, the solution was cooled down to room temperature and then concentrated in *vacuo*. The next, and more critical step, is the recrystallization of the compound to remove impurities coming from $[Ru(CO)_2Cl_2]_n$. The obtained solid was dissolved in 20 ml of dichloromethane (complete dissolution took around 15 min). The liquid was transferred to a heart shaped flask, then pentane was added until the solution appeared cloudy, and a solid crashes out of the solution. After cooling the mixture in the fridge overnight, the final mass of $[Ru(CO)_3Cl_2]_2$ obtained after filtration was

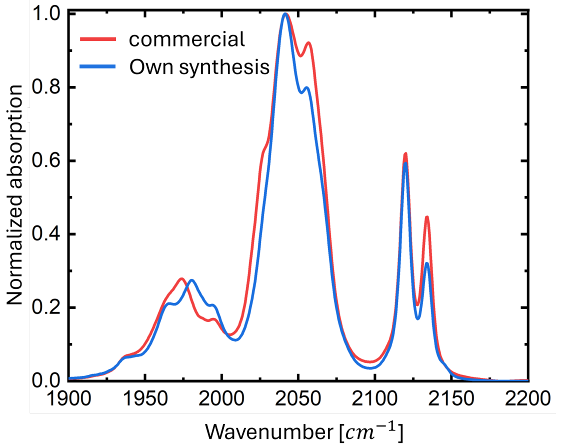

Figure S3: Comparison of FTIR spectra of a commercially obtained complex $[Ru^{II}(CO)_3Cl_2]_2$ in red with our synthesized one in blue. Solvent: DMF

1.7 g. To confirm successful synthesis, we compared the FTIR spectra of our product to a commercially obtained compound, see Fig. S3.

IR (DMF,cm^{-1}): 2041.0 (s, CO), 2057.1 (s,CO) ,2120.1 (s,CO), 2133.6 (s,CO)

1.3 Synthesis of *trans*(Cl)-Ru^{II}(X,X'-dmbpy)$(CO)_2(Cl)_2$ (Fig. S1c)

We followed the protocol of Ref.[5] The starting materials are 203.3 mg of $[Ru(CO)_2Cl_2]_n$ previously synthesized and 133.0 mg of dimethyl-X,X'-bipyridine, in a N_2 purged (5-10 min) solution of 20 ml MeOH in a 50 ml two-necked round-bottom flask equipped with a magnetic stirrer. The reaction is performed under reflux conditions at 85 °C for 2 h. After a few minutes, a yellow precipitate becomes observable. Once the reflux is complete, the solid is isolated using a Büchner funnel with a MN 615 filter paper. The solid is then dried under vacuum of 3 mbar for 3 h. The amount of *trans*(Cl)-Ru(6,6'-dmbpy)$(CO)_2(Cl)_2$] obtained was 151 mg, with a yield of 40 %.

The presence of $[Ru(CO)_3Cl_2]_2$ leads to the formation of *cis*(Cl)-Ru(X,X'-dmbpy)$(CO)_2(Cl)_2$. Adding one drop of HCl 32 %[8] and refluxing it under the same conditions as before for 1 h reduced the amount of impurity. The procedure was repeated until the impurity was insignificant, checking the NMR spectrum.

IR (DMF,cm^{-1}): 1989 (s, sym CO stretch), 2055 (s, as CO stretch)

^{1}H-NMR 6,6'-dmbpy in DMSO-d6, 400MHz, δ (ppm): 8.55 (dd, 2H, J_{23}=7.8Hz, J_{13}=1.2Hz), 8.21 (t, 2H, J_{12}=7.8Hz, J_{23}=7.8Hz), 7.74 (dd, 2H, J_{12}=7.8Hz, J_{13}=1.2Hz), 3.15 (s, 6H).

^{1}H-NMR 5,5'-dmbpy in DMSO-d6, 400MHz δ (ppm): 9.01 (t, 2H, J_{12}=1.9Hz, J_{13}=0.9Hz), 8.63 (dd, 2H, J_{23}=8.3Hz, J_{13}=0.9Hz), 7.74 (dd, 2H, J_{23}=8.3Hz, J_{12}=1.9Hz), 2.53 (s, 6H).

^{1}H-NMR 4,4'-dmbpy in DMSO-d6, 400MHz, δ (ppm): 9.03 (dd, 2H, J_{12}=5.6Hz, J_{13}=0.9Hz), 8.64 (t, 2H, J_{23}=1.8Hz, J_{13}=0.9Hz), 7.66 (dt, 2H, J_{12}=5.6Hz, J_{23}=1.8Hz), 2.59 (s, 6H).

1.4 Synthesis of *cis*(Cl)-Ru^{II}(X,X'-dmbpy)$(CO)_2(Cl)_2$ (Fig. S1d)

We followed the protocol of Ref.[5] The starting materials are 152.9 mg of $[Ru(CO)_3Cl_2]_2$ previously synthesized and 112.7 mg of dimethyl-X,X'-bipyridine, in a solution of 15 ml THF. The solvent was purged with N_2 for 5 to 10 min in a 50 ml two-necked round-bottom flask with a magnetic stirrer prior to adding both solids then the reaction mixture was kept under reflux conditions at 90 °C for 1 h 20 min. After a few minutes, an orange precipitate is observable. Once the reflux is complete, the solid was isolated using a Büchner funnel with filter paper MN 615. The solid was dried under reduced pressure of 3 mbar for 3 h. The amount of *cis*(Cl)-Ru(5,5'-dmbpy)$(CO)_2(Cl)_2$] obtained was 161.2 mg, with a yield of 70 %, and for *cis*(Cl)-Ru(4,4'-dmbpy)$(CO)_2(Cl)_2$] the amount was 89 mg, being the yield 43 %. For *cis*(Cl)-Ru(6,6'-dmbpy)$(CO)_2(Cl)_2$] there was no clear NMR signal, the fact that the purification of cis-Ru6dmb was unsuccessful can be explained by steric hindrance between a Cl in equatorial position and a methyl group, enhancing the distortion of the bpy ring.

We also explored another synthetic route from Ref.[2] In this case we used 250 mg of previously synthesized $[Ru(CO)_3Cl_2]_2$ and 189 mg of dimethyl-5,5'-bipyridine. The precursor dimer is added to 30 mL of THF in a two-necked round-bottom flask under N_2. This solution is kept under reflux conditions (85°C) for 3 hours. Then, the dimethyl-5,5'-bipyridine ligand is dissolved in 5 mL of THF in a beaker. While interrupting the reflux, the ligand solution was added drop wise to the stirred reaction mixture. Then the solution is refluxed for

another 45 minutes. After a few minutes, the colour of the solution changes from a pale yellow to reddish. It is important to seal the reaction flask well, since the precursor dimer is a CO releasing molecule, so is the product. This might lead to a lower reaction yield. Once the reflux is complete, the solid was isolated using a Büchner funnel with filter paper MN 615. The solid was dried under reduced pressure of 3 mbar for 3 h. The amount of *cis*(Cl)-Ru(5,5'-dmbpy)$(CO)_2(Cl)_2$] thus obtained was 232 mg, being the yield 58 %.

^{1}H-NMR *cis*(Cl)-4,4'-dmbpy in DMSO-d6, 400MHz δ (ppm): 9.22 (d, J=5.8Hz, 1H), 8.75 (d, J=5.8Hz, 1H), 8.63 (s, 1H), 8.59 (s, 1H), 7.76 (dt, J=[5.8, 1.9, 0.9]Hz, 1H), 7.51 (dt, J=[5.8, 1.9, 0.9]Hz, 1H), 2.60 (s, 3H), 2.54 (s, 3H).

^{1}H-NMR *cis*(Cl)-5,5'-dmbpy in DMSO-d6, 400MHz δ (ppm): 9.19 (s, 1H), 8.78 (s, 1H), 8.61 (d, J=8.3Hz, 1H), 8.56 (d, J=8.3Hz, 1H), 8.21 (d, J=8.3Hz, 1H), 8.08 (d, J=8.3Hz, 1H), 2.52 (s, 3H), 2.44 (s, 3H).

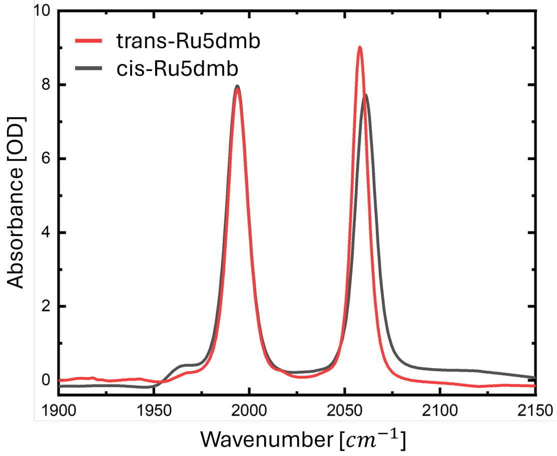

Figure S4: FTIR spectra of *trans*(Cl)-Ru5dmb and cis-Ru5dmb in DMF.

In Fig. S4, a small up-shift is observed for the higher frequency band of *cis*(Cl)-Ru(5,5'-dmbpy)$(CO)_2(Cl)_2$] relative to *trans*(Cl)-Ru(5,5'-dmbpy)$(CO)_2(Cl)_2$]. This spectral signature actually appears in the TRIR data of Ru5dmb, see Fig. 9, and we conclude that *cis*(Cl)-Ru(5,5'-dmbpy)$(CO)_2(Cl)_2$] is in fact an intermediate in the photo-reaction (see main text for details).

1.5 Synthesis of Ru^{II} (6,6'-dmbpy)$(CO)_2$ClH (Fig. S1e)

For the synthesis of Ru^{II} (6,6'-dmbpy)$(CO)_2$ClH, a procedure by Kubiak and coworkers for a slightly different complex was adapted.[9] In a 2 necked-round bottom flask with 10 ml of dry methyl formate, which was bubbled during 10 min with N_2 using a glass needle, 0.13 g of 6,6'-dimethyl-bipyridyl and 0.25 g of $[Ru(CO)_3Cl_2]_2$ were added and the reaction occurred under reflux conditions at 90 °C for 1.5 h.

After the reaction was complete, the solvent was removed by rotary evaporation until dryness, then it was mixed with 3 g of neutral alumina and stirred in presence of 5 ml of DCM. After 10 min of mixing, the solvent was removed again. The remaining solid was suspended in DCM and loaded onto a SiO_2 column. The column was first washed with ethyl acetate to remove unreacted ligand. The remaining sample was eluted with a gradient from 0 % to 10 % of MeOH in DCM. The isolation of the Ru-H from the obtained mixture was not possible. Nevertheless, a singlet peak appearing at: δ -10.2 in the ^{1}H-NMR (d6-DMSO) indicates the presence of a hydride, supporting the hypothesis explained in the main manuscript.

1.6 Unsuccessful Synthesis of $[Ru^{I}$ (6,6'-dmbpy)$(CO)_2(Cl)]_2$ (Fig. S1f)

The dimerization of Ru4dmb and Ru5dmb has been described previously.[2,10] We followed a protocol from Connor and coworkers in our attempt to isolate the Ru6dmb-dimer,[3] which however was unsuccessful. For this synthesis, 300 mg of $[Ru^{II}(CO)_3Cl_2]_2$ were dissolved in 15 ml of ethylene glycol. Then, 500 mg of 6,6'-dimethyl-bipyridine was added and heated at 120°C during 12 h under N_2 atmosphere. After 4 days at room temperature with N_2, no precipitate was observable. To push the reaction even further, 12 ml of octanol were added and the reaction mixture was heated to 75°C for 7 h. Again, after another 4 days at room temperature under N_2 atmosphere no precipitate was observable.

2 Transient IR Spectroscopy

For transient IR experiments two different laser setups covering different time windows were used: Short timescales (10 ps to ~40 μs) were measured using two 2.5 kHz Ti: sapphire laser systems that were electronically synchronized.[11] One laser was used to generate 420 nm excitation pulses with ~100 fs duration by frequency doubling of the 840 nm fundamental in a BBO crystal. The excitation pulses had ~1 μJ of energy and ~150 μm spot size in the interaction region. The other laser was used to produce mid-IR probe pulses centered at 2000 cm^{-1} via a home-built OPA.[12] The probe was detected with a 2x64 element, mercury cadmium telluride (MCT) mid-IR array detector with a spectral resolution of approximately 4 cm^{-1} per pixel, and a home-built electronics for single-shot digitization and readout.[13]

A second time window (~100 ns to ~300 ms) was scanned using a single-VIS-pump-multiple-IR-probe arrangement. A single visible pump pulse centered at 447 nm (4.5 μJ, 400 μm spot size, 500 ns duration) was generated with a GaN multimode laser diode (PLPT9 450D_E, Osram Opto Semiconductors) operated by a pulsed laser diode driver (LDP-V 10-10, PicoLAS) at 2 Hz rep rate, followed by multiple IR probe pulses from a Yb-doped fiber laser system at 100 kHz (Tangerine, Amplitude, France) pumping an optical parametric amplifier (OPA) (Twin STARZZ, Fastlite, France). In this case the detector was a 2x32 MCT array with ~6 cm^{-1} spectral resolution per pixel.

In both TRIR experimental setups the sample was measured in a home-built flow-cell consisting of two CaF_2 windows (thickness of 2 mm) with a 100 μm thick PTFE spacer between them and encapsulated in a metal frame. In the short-timescale setup, the sample was exchanged continuously using a peristaltic pump (Ismatec 935C, Wertheim, Germany, with ACIDFLEX tubing in the pump chamber). In the long-timescale setup, the flow was pulsed, see Ref.[14] for details. The sample reservoir was purged with Ar gas for the entire duration of the experiment. The Ar gas was pre-saturated with the solvent by bubbling it through a separate reservoir to avoid the loss of solvent in the main sample reservoir.

3 Catalyst Photochemistry

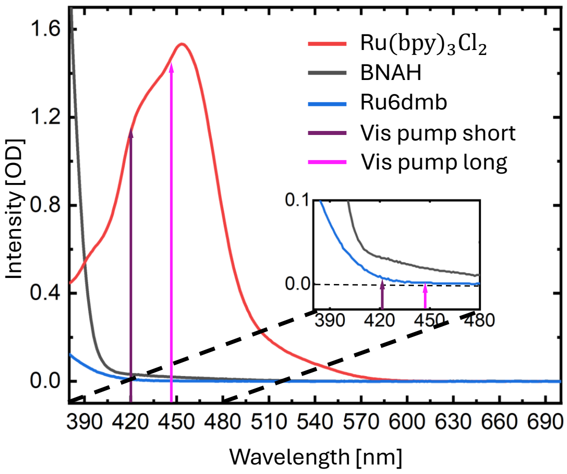

Figure S5: UV-Vis spectra (measured in a UV-2450 UV–Vis spectrophotometer, Shimadzu) of a typical sample with 20 mM catalyst, 100 mM BNAH, and 10 mM PS in DMF. The measurement cell consisted of two 2 mm thick CaF_2 windows and a 100 μm thick PTFE spacer in between. The figure also shows the wavelength of the actinic pump pulse for the two TRIR laser setups used in this study. The inset zooms into the absorption tale of the catalyst.

The RuXdmb family of catalysts are carbon monoxide releasing molecules when the metal center is directly excited.[15–17] Due to the rapid release of CO, this can be only observed in the laser set up for short time scales. As explained in Sec. 2, the excitation pulses are centered around 420 nm in that setup, with a tunability of plus/minus a few nanometers via the phase-matching angle of the doubling crystal.

The properties of Ru4dmb, Ru5dmb and Ru6dmb are equivalent with regard to photodegradation, and we exemplify it here for Ru4dmb. The obtained TRIR data are shown in Fig. S6b) with two bleaches at 1990 and 2056 cm^{-1} belonging to the ground state Ru4dmb. At the earliest delay of the data (10 ps), a single positive band at 1972 cm^{-1} is observed, pointing to the release of an equatorial CO, see compound **[a]** Fig. S7. The band at 1972 cm^{-1} then evolves rapidly to 1940 cm^{-1} with time constant τ_{ab}=60 ps, which indicates the introduction of a solvent molecule at the empty binding site (see compound **[b]** in Fig. S7). In either case, the structural assignment have been made based on the fact that only one pos-

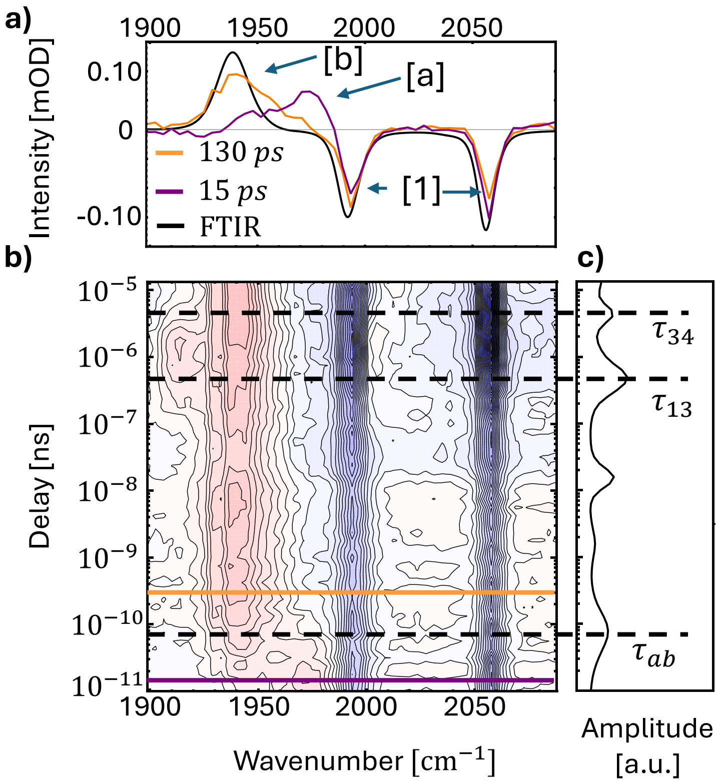

Figure S6: (b) TRIR spectrum of Ru4dmb from 10 ps to 10 μs with the respective dynamical content in panel (c) as well as spectral cuts at 15 ps and 130 ps compared to a steady state FTIR difference spectrum in panel (a). Experimental conditions: 20 mM Ru4dmb, 1 mM $Ru(bpy)_3Cl_2$, 100 mM BNAH in DMF, excitation wavelength 420 nm. Time constant τ_{ab} is related the introduction of a solvent molecule at the empty binding site after CO loss, time constant τ_{13} to the first reduction step and subsequent chloride loss of the still intact catalyst, and time constant τ_{34} to the subsequent ligand exchange.

itive band is observed (i.e., only one carbonyl ligand). The transient difference spectrum measured at 130 ps (Fig. S6a, orange line) agrees with a steady state spectrum measured after continuous illumination (black line), indicating that compound **[b]** is a stable product, which most likely is *cis*(Cl)-Ru(4,4-bmbpy)(CO)(DMF)Cl_2 according to literature.[15]

In the experiments described in the main paper, we suppressed accumulation of the photo-degraded catalyst to the extent it is no longer visible in the TRIR data by (a) shifting the pump laser as much as possible to the red, and at the same by (b) adding PS at relatively high concentrations, hence the majority of the pump light is absorbed by the PS instead of the catalyst.

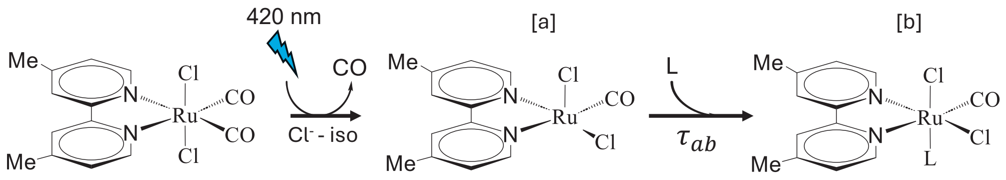

Figure S7: Reaction pathway of photo-degradation upon direct excitation of the catalyst, as deduced from transient IR spectroscopy.

4 TRIR Control Experiments

Figs. S8 and S9 show TRIR experiments with certain components of the overall photochemical system changed and/or their concentration varied. That is, Fig. S8 test the effect of water in the solution, and Fig. S9 replaces the PS $Ru(bpy)_3Cl_2$ by $Ru(bpy)_3(PF_6)_2$ and then varies the concentration of additional chloride. The results of these experiments are discussed in the main text.

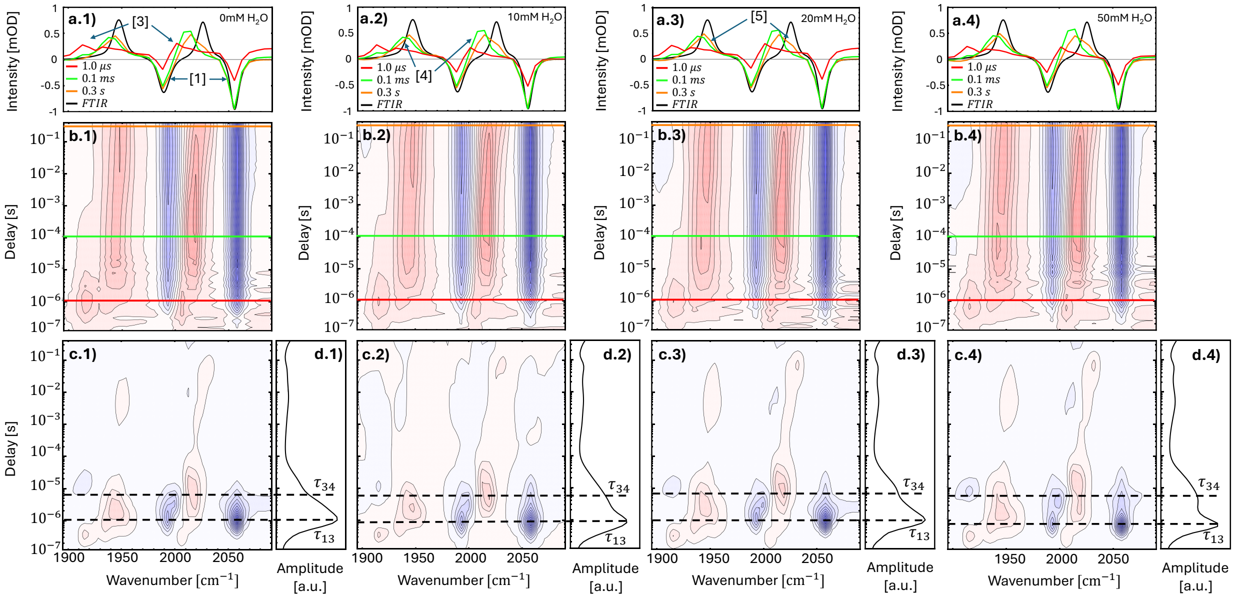

Figure S8: Water concentration dependence: (b1) Transient IR spectra of Ru6dmb with increasing amounts of water, i.e., 0 mM in panel (b1), 10 mM in panel (b2), 20 mM in panel (b3), and 50 mM in panel (b4). Panels (a1)-(a4) show temporal cuts at the selected delay times indicated by the equally colored horizontal lines in panels (b1)-(b4), together with a steady-state FTIR difference spectrum upon continuous illumination (in black). Panels (c1)-(c4) show the lifetime density maps for the transient IR spectra (b1)-(b4), whereas panels (d1)-(d4) show the corresponding dynamical content. The time constants in panel (d1) are τ_{13}=1 μs and τ_{34}=6 μs, in panel (d2) τ_{13}=0.8 μs and τ_{34}=6 μs, in panel (d3) τ_{13}=1 μs and τ_{34}=7 μs, and in panel (d4) τ_{13}=0.8 μs and τ_{34}=6 μs. Experimental conditions: 20 mM Ru6dmb, 10 mM Ru(bpy)$_3$Cl$_2$, 100 mM BNAH in DMF, TBACl in increasing amounts, excitation wavelength 447 nm.

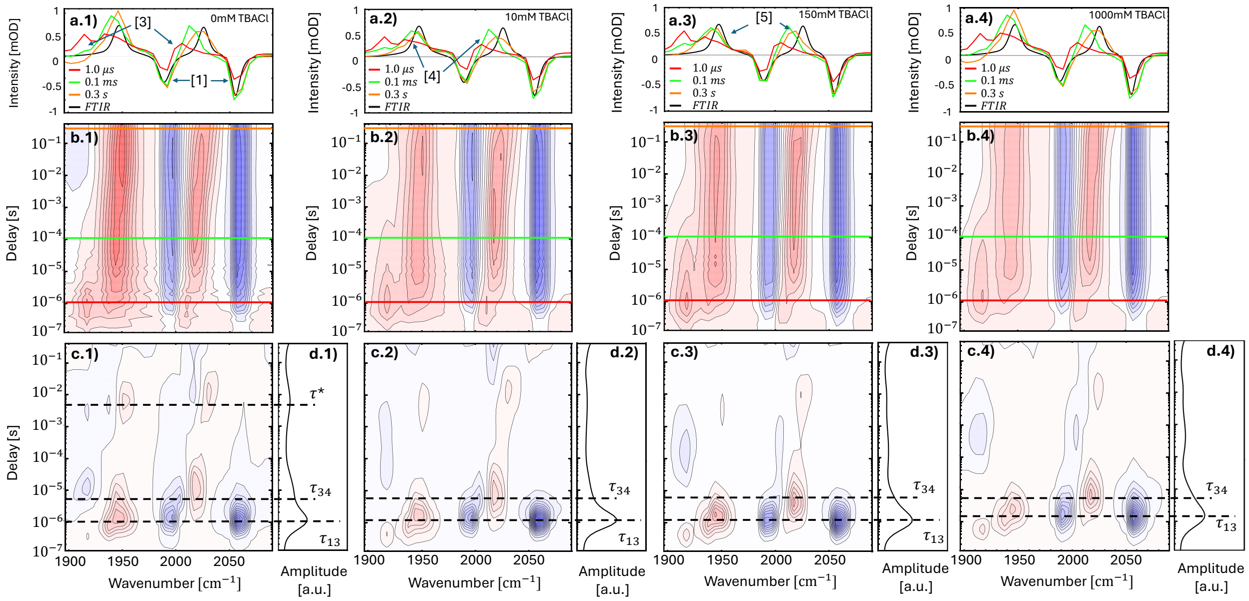

Figure S9: Chloride concentration dependence. (b1) Transient IR spectra of Ru6dmb with increasing amount of TBACl, i.e., 0 mM in panel (b1), 10 mM in panel (b2), 150 mM in panel (b3), and 1 M in panel (b4). Panels (a1)-(a4) show temporal cuts at the selected delay times indicated by the equally colored horizontal lines in panels (b1)-(b4), together with a steady-state FTIR difference spectrum upon continuous illumination (in black). Panels (c1)-(c4) show the lifetime density maps for the transient IR spectra (b1)-(b4), whereas panels (d1)-(d4) show the corresponding dynamical content. The time constants in panel (d1) are τ_{13}=1 μs, τ_{34}=5 μs and τ^*=5 ms , in panel (d2) τ_{13}=1.1 μs and τ_{34}=6 μs, in panel (d3) τ_{13}=1.2 μs and τ_{34}=6 μs, and in panel (d4) τ_{13}=1.5 μs and τ_{34}=5 μs. Experimental conditions: 20 mM Ru6dmb, 10 mM $Ru(bpy)_3(PF_6)_2$, 100 mM BNAH in DMF, TBACl in increasing amounts, excitation wavelength 447 nm.

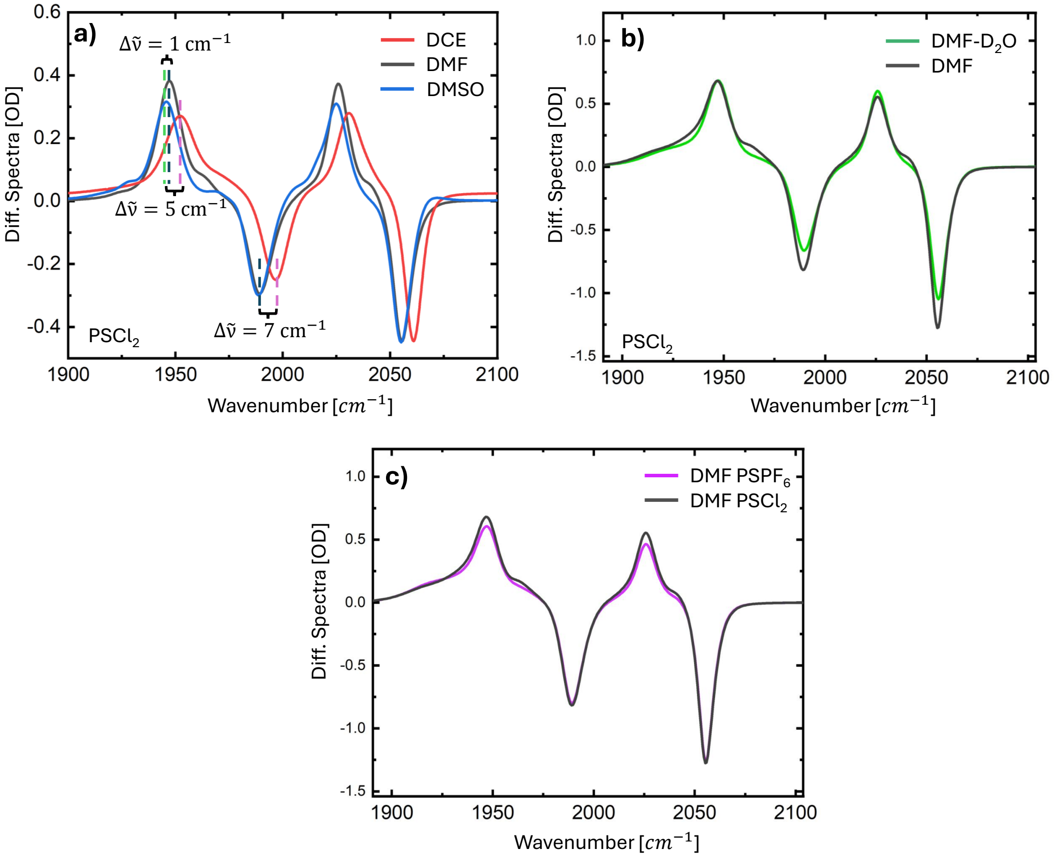

Figure S10: Stationary FTIR difference spectra of Ru6dmb/Ru(bpy)$_3$Cl$_2$ upon continuous illumination. (a) Comparing the system in different solvent, DMF, DMSO and DCE, (b) comparing the original system in DMF with one where an excess (1.8 M) of D_2O has been added, and (c) comparing the original system in DMF with one where Ru(bpy)$_3$(PF$_6$)$_2$ has been used as PS instead of Ru(bpy)$_3$Cl$_2$. Experimental conditions otherwise: 20 mM Ru6dmb, 10 mM PS, 100 mM BNAH, excitation wavelength 447 nm.

5 FTIR Control Experiments

Additional evidence for the fact that the final product no longer ligates a solvent molecule is provided in Fig. S10a, which compares stationary FTIR difference spectra of Ru6dmb upon continuous illumination at 447 nm in DMF with the same measurement in 1,2-dichloroethane (DCE). Both difference spectra are in essence the same, apart from a small solvatochromic shift. DCE is a non-ligating solvent, but ligation of DMF in the final product would generate a much larger change due to the removal of the chloride charge. It would have been illustrative

to perform also time-resolved experiments in DCE, but due to the low solubility of the compounds, in particular of the PS, these turned out to be impossible.

Fig. S10a also shows the stationary FTIR difference spectrum in DMSO, with vibrational frequencies that are practically identical to the ones in DMF. It is thus the same photoproduct, which justifies that we measured the ^{1}H-NMR spectra of Fig. 6 in the main paper in DMSO, drawing conclusions on the photo-reaction in DMF (DMF bands obscure the interpretation of the ^{1}H-NMR-spectra).

Fig. S10b compares the FTIR difference spectrum of the original system with one where an excess of D_2O has been added, in which case the final photo product is a deuterated hydride. The spectrum is essentially the same, indicating that the oscillator strength of the Ru-H vibration of the original system, which is expected to absorb in the same spectral range as the CO vibrations, is negligible.

Finally, Fig. S10c shows the FTIR difference spectrum when reducing the Cl^- concentration as much as possible by using $Ru(bpy)_3(PF_6)_2$ instead of $Ru(bpy)_3Cl_2$ as PS. The difference spectrum again is the same, indicating that re-chlorination is still occurring.

6 NMR Experiments

This section contains ^{1}H-NMR spectra of each of the important components in our chemical system. All spectra have been measured in Bruker Avance Neo 400 MHz spectrometer. The solvent residual peak was used as a reference in all of the spectra. Mnova NMR software (Mestrelab) was used for multiplet analysis to identify weaker coupling constants (J_4 (meta) and J_5 (para)) in addition to clearly resolved J_3 ones. The spectra of the individual components aid identification of the chemical species formed after the photo-induced reaction.

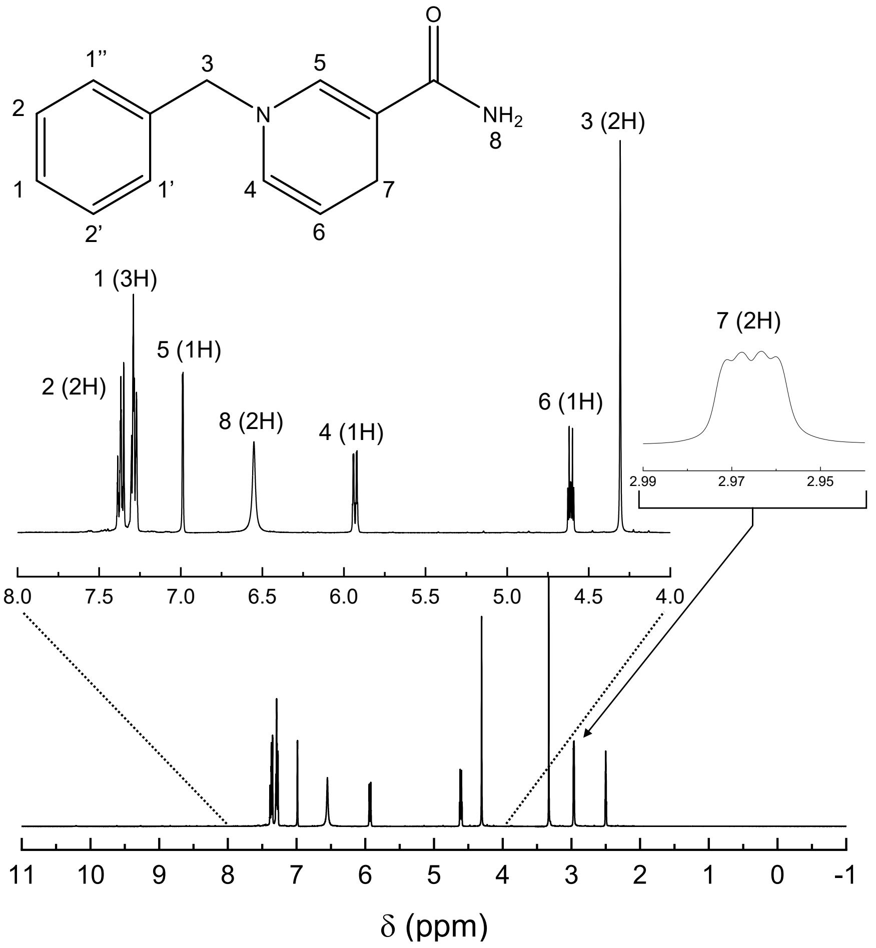

Figure S11: ^{1}H-NMR spectrum of BNAH in DMSO-d6, 400MHz, δ (ppm): 7.40-7.34 (m, 2H), 7.32-7.25 (m, 3H), 6.99 (d, 1H, J_{45}=1.6Hz), 6.55 (br s, 2H), 5.93 (dq, 1H, J_{46}=8.0Hz, J_{45}=1.6Hz, J_{47}=1.6Hz), 4.61 (dt, 1H, J_{46}=8.0Hz, J_{67}=3.5Hz), 4.31 (s, 2H), 2.97 (dd, 2H, J_{67}=3.5Hz, J_{47}=1.6Hz)

We start the analysis with the ^{1}H-NMR spectra of the sacrificial electron donor, BNAH,

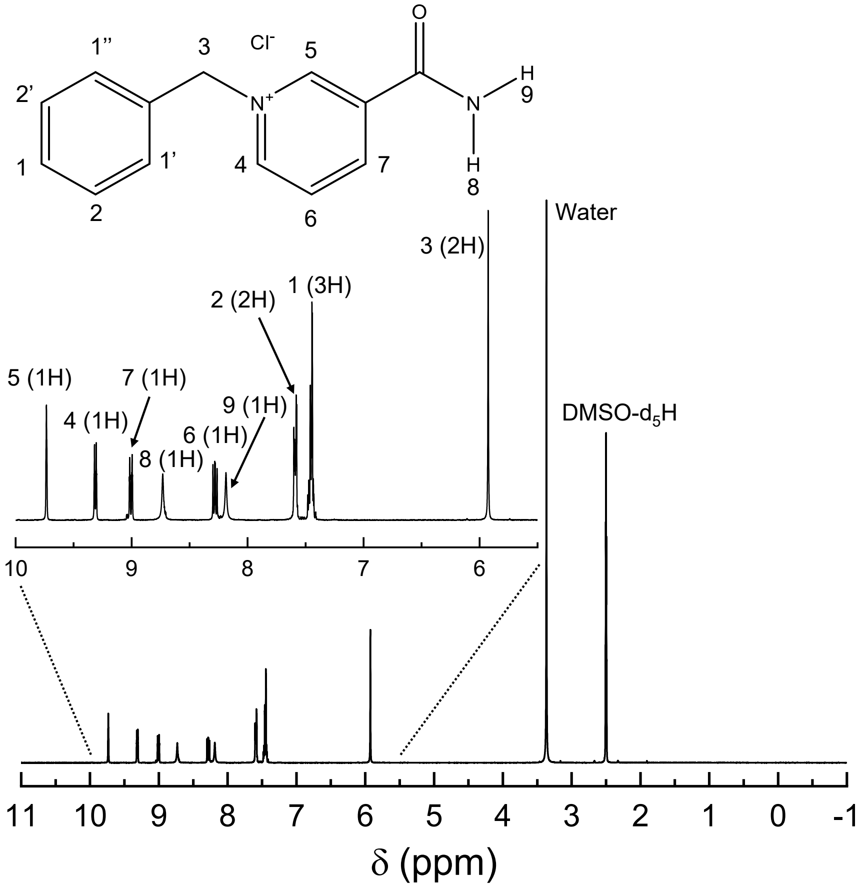

Figure S12: ^{1}H-NMR spectrum of BNA$^+$ chloride in DMSO-d6, 400MHz, δ (ppm): 9.73 (t, 1H, J_{45}=1.3Hz, J_{57}=1.3Hz), 9.31 (dt, 1H, J_{46}=6.2Hz, J_{45}=1.3Hz, J_{47}=1.3Hz), 9.01 (dt, 1H, J_{67}=8.2Hz, J_{57}=1.6Hz, J_{47}=1.3Hz), 8.73 (br s, 1H), 8.28 (dd, 1H, J_{67}=8.2Hz, J_{46}=6.2Hz), 8.19 (br s, 1H), 7.62-7.56 (m, 2H), 7.49-7.41 (m, 3H), 5.93 (s, 2H).

and the product of its double-oxidation, BNA$^+$) (Figs. S11 and S12). Both are commercially available (see Table S1). The figures also show our assignment of the observed peaks. Comparing the two spectra, one can notice a few important changes, especially above 8 ppm. BNA$^+$ loses a proton from the position 7, as labelled in Fig. S11. As a result, a second aromatic ring is formed in BNA$^+$, which is responsible for the signals in the aforementioned downfield region and also the splitting of the -NH_2 singlet in the BNAH into the two singlets in BNA$^+$. The ^{1}H-NMR spectrum of the photosensitizer, $[Ru(bpy)_3]^{2+}$, shows only 4 non-equivalent protons (see Fig. S13). This is to be expected since the 3 bpy rings are equivalent due to the symmetry, as the molecule belongs to the D_3 point group. In addition, the two

pyridine parts of each bpy ligand are related by a mirror plane symmetry. Similarly to the PS, the catalyst - Ru6dmb - spectrum has 4 non-equivalent protons (see Fig. S14). The other two versions of the catalyst, Ru4dmb and Ru5dmb (see Fig. S15 and S16), display ^{1}H-NMR spectra closely resembling that of the Ru6dmb. The most significant difference between them is the chemical shift of the methyl groups. While in the ^{1}H-NMR spectrum of Ru6dmb the methyls' singlet is at 3.15 ppm, in the two other version of the catalyst it is much closer to the solvent residual band. The reason for the latter is that the methyl groups in the Ru4dmb and Ru5dmb are located further away from the electron-withdrawing nitrogen on the bpy, so they are more shielded.

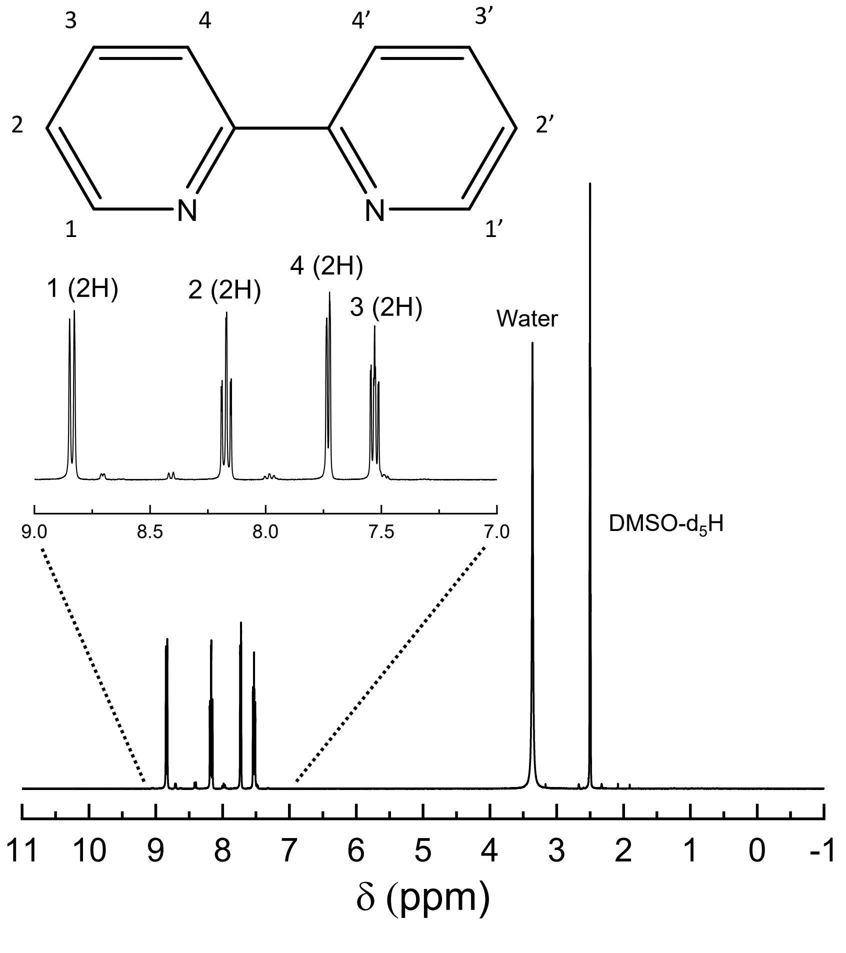

Figure S13: ^{1}H-NMR spectrum (400MHz) of the PS, $Ru(bpy)_3(PF_6)_2$ in DMSO-d6, δ (ppm): 8.88 (dt, 2H, J_{12}=8.2Hz, J_{13}=1.4Hz, J_{14}=0.7Hz), 8.17 (td, 2H, J_{12}=8.2Hz, J_{23}=7.6Hz, J_{24}=1.4Hz), 7.73 (dt, 2H, J_{34}=5.7Hz, J_{24}=1.4Hz, J_{14}=0.7Hz), 7.54 (ddd, 2H, J_{23}=7.6Hz, J_{34}=5.7Hz, J_{13}=1.4Hz).

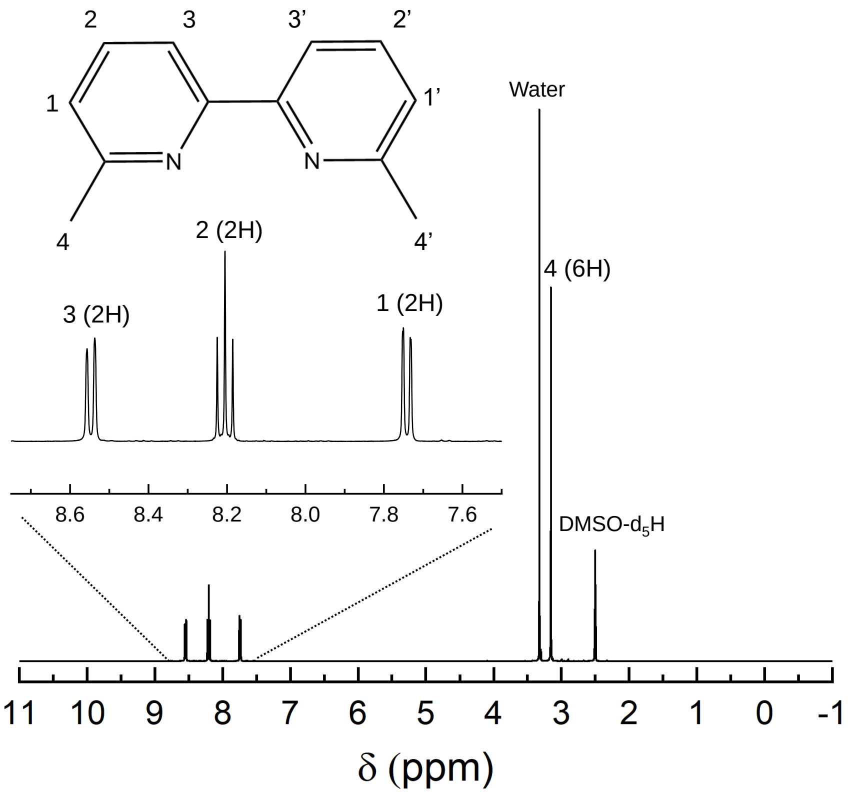

Figure S14: ^{1}H-NMR spectrum (400MHz) of the catalyst, Ru6dmb in DMSO-d6, δ (ppm): 8.55 (dd, 2H, J_{23}=7.8Hz, J_{13}=1.2Hz), 8.21 (t, 2H, J_{12}=7.8Hz, J_{23}=7.8Hz), 7.74 (dd, 2H, J_{12}=7.8Hz, J_{13}=1.2Hz), 3.15 (s, 6H).

Fig. S17 shows an extended version of Fig. 6 in the main paper, comparing the chemical system before and after 10 minutes of irradiation with 447 nm laser, as well as the synthetically produced mixture containing an unknown concentration of Ru^{II}(6,6'-dmbpy)$(CO)_2$ClH (see section 1.5 for more details). Most importantly, this experiment enables us to decide whether BNAH acts as a two-electron donor. After the one-electron oxidation of BNAH and a proton loss from position 7 (using the labelling scheme from Fig. S11), the $BNA^\bullet$ can dimerize or donate another electron and form BNA^+. BNA_2 exists in two isomeric forms (4,4'- and 4,6-), whose ^{1}H-NMR spectra in DMSO-d6 have been reported by Ohnishi and Kitami.[18] There is no evidence of any of these BNA_2 species in our ^{1}H-NMR spectrum of the irradiated chemical system (Fig. S17b). The latter spectrum shows an almost full depletion of the peaks related to BNAH, as evidenced by the absence of the broad singlet at 6.55 ppm,

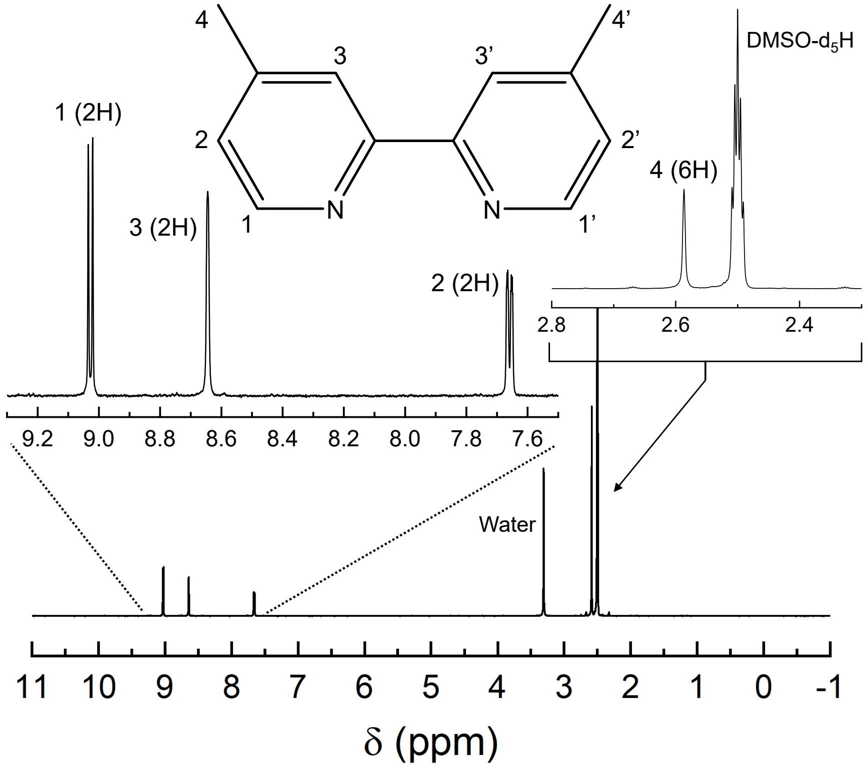

Figure S15: ^{1}H-NMR spectrum (400MHz) of Ru4dmb in DMSO-d6, δ (ppm): 9.03 (dd, 2H, J_{12}=5.6Hz, J_{13}=0.9Hz), 8.64 (t, 2H, J_{23}=1.8Hz, J_{13}=0.9Hz), 7.66 (dt, 2H, J_{12}=5.6Hz, J_{23}=1.8Hz), 2.59 (s, 6H).

as well as the multiplet at 2.97 ppm. Instead, we get peaks in the downfield region that are characteristic to BNA^+. On the first look one may confuse it with the peak at 5.93 ppm, which is also present in 4,4'-BNA_2 spectrum, but it's a doublet in the latter whereas in our results it is a singlet (Fig. S17b). The 5.93 ppm peak in Fig. S17b perfectly overlaps with the one of BNA^+ chloride (Fig. S12). There is no band in Fig. S17b that would agree with BNA_2,[18] hence we conclude that BNAH acts as a 2-electron donor.

Finally, Fig. S18 shows an extended version of Fig. 12 in the main paper, exploring dimerization of Ru5dmb. The spectrum after irradiation (Fig. S18) shows a depletion of the original singlet related to the methyl protons at 2.61 ppm, accompanied by a number of new methyl peaks in the 2.35-2.62 ppm range. Based on these results, we concluded that the irradiation product consists of a symmetry-broken *cis*(Cl)-[Ru^{II}(5,5'-dmbpy)$(CO)_2Cl_2$] isomer, i.e., the two more intense bands at 2.52 and 2.57 ppm, as well as many smaller bands

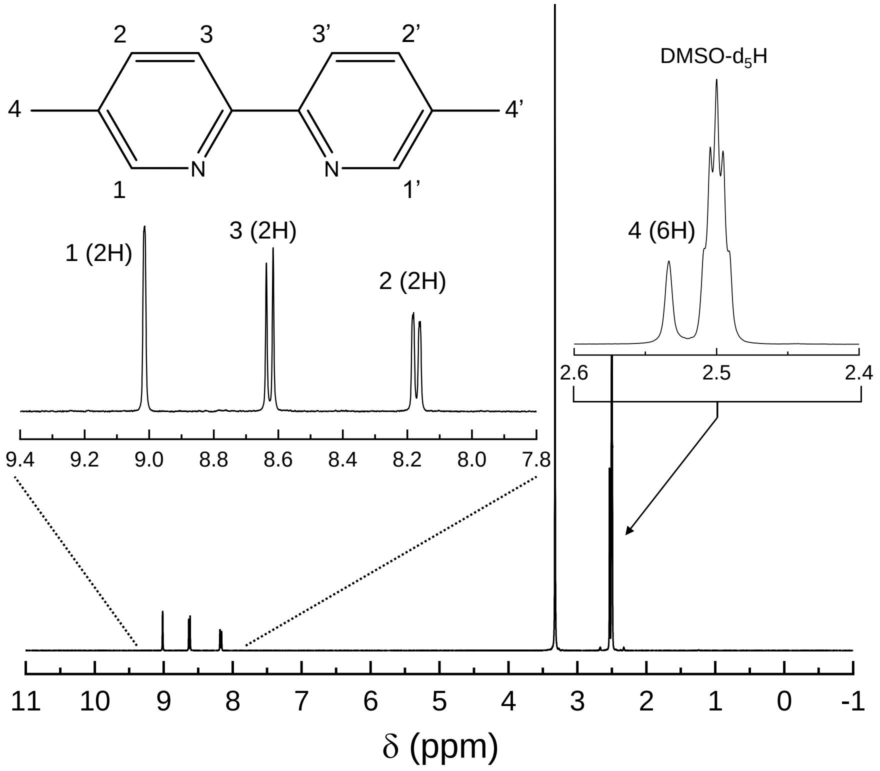

Figure S16: ^{1}H-NMR spectrum (400MHz) of Ru5dmb in DMSO-d6, δ (ppm): 9.01 (t, 2H, J_{12}=1.9Hz, J_{13}=0.9Hz), 8.63 (dd, 2H, J_{23}=8.3Hz, J_{13}=0.9Hz), 7.74 (dd, 2H, J_{23}=8.3Hz, J_{12}=1.9Hz), 2.53 (s, 6H).

related to different polymer species.

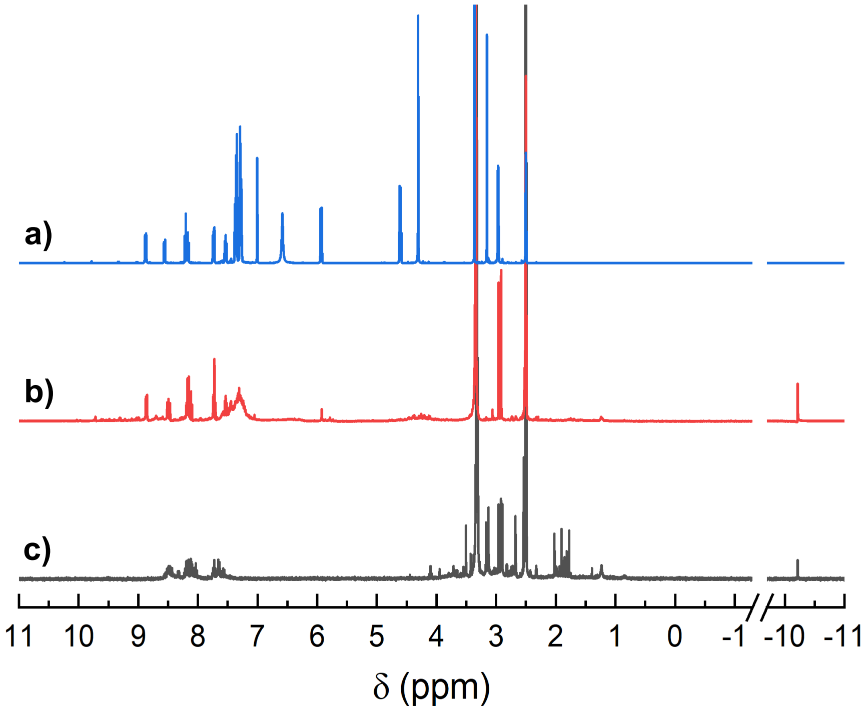

Figure S17: Exploring the hydride formation in Ru6dmb, using ^{1}H-NMR spectroscopy, (a) before irradiation, (b) after 10 min of irradiation, and (c) compared to synthetically produced mixture containing Ru^{II}(6,6'-dmbpy)$(CO)_2$ClH (unknown concentration, see Sec. 1.5 above). Experimental conditions: 7 mM Ru6dmb, 3 mM $Ru(bpy)_3Cl_2$, 25 mM BNAH in DMSO-d_6, 400 MHz, excitation wavelength 447 nm.

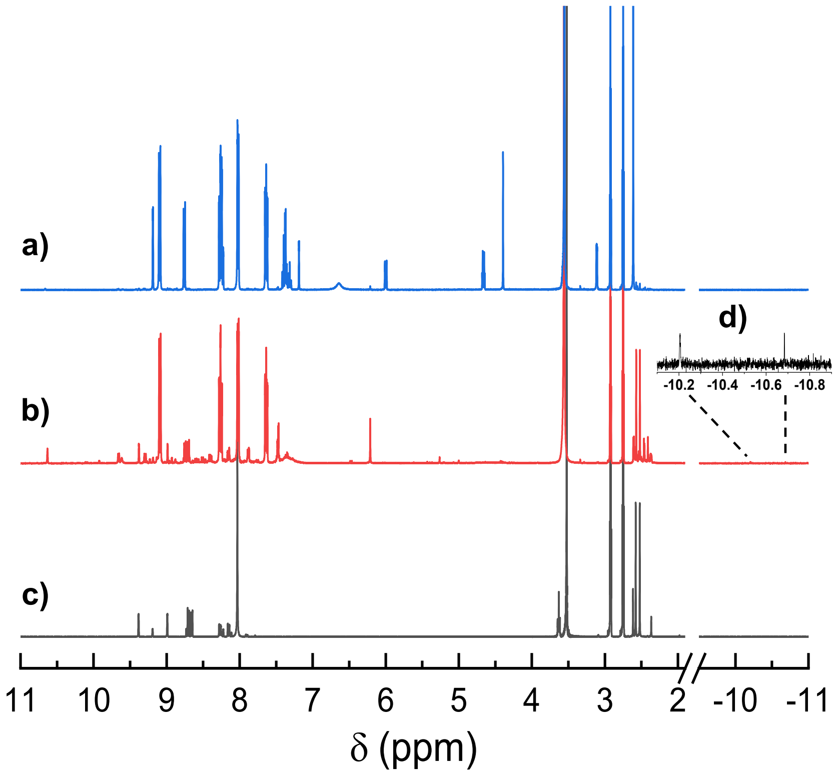

Figure S18: Exploring dimerization of Ru5dmb using ^{1}H-NMR spectroscopy, (a) before irradiation, (b) after 3 min of irradiation, and (c) compared to synthetically produced *cis*(Cl)-[Ru^{II}(5,5'-dmbpy)$(CO)_2Cl_2$] (contains impurities). Inset (d) shows a very small fraction of a Ru-H species present at the end of the photo-induced reaction. Experimental conditions: 10 mM Ru5dmb, 10 mM $Ru(bpy)_3Cl_2$, 10 mM BNAH in DMF-d_7, 400 MHz, excitation wavelength 447 nm.

7 DFT calculations

DFT calculations have been performed with the Gaussian 16 package[19] using the ωB97X-D functional.[20] Structure optimization and frequency calculation was done with the def2-TZVP basis set.[21] The basis set includes an effective core potential (ECP) for the Ru atom, reducing the cost of computation. Solvation was included using polarizable continuum model (PCM)[22] with the default integral equation formalism variant. The minimum of the structure optimization was cross-validated by the absence of imaginary frequencies in the normal mode analysis. The vibrational frequencies, as well as zero point vibrational energies have been scaled by an empirical factor of 0.9496, determined to best fit the experimental vibrational frequencies of the resting state of the catalyst. Additionally, single point energy calculations were performed to obtain more accurate electronic energies using the def2-QZVPPD basis set.[21]

Reaction energies were calculated by subtracting the sum of Gibbs free energies (obtained from the standard thermochemical output of Gaussian[23]) of reactants from the equivalent sum for the products. An example is shown below which calculates the reaction energy of the final complex process of proton transfer, re-chlorination, and an electron transfer:

$$\begin{aligned}&\text{(iso)-[Ru}^{\text{I}}\text{(6,6'-dmbpy)(CO)}_2\text{DMF]}^{\bullet+} + \text{[BNAH]}^{\bullet+} + \text{Cl}^- \rightleftharpoons \\ &\quad \text{Ru}^{\text{II}}\text{(6,6'-dmbpy)(CO)}_2\text{(Cl)H}^{\text{(Equat.)}} + \text{BNA}^+ + \text{DMF}\end{aligned} \tag{1}$$

$$\begin{aligned}&\Delta G^{298\text{K}}_{\text{rxn}} = \Delta G^{298\text{K}}_{\text{products}} - \Delta G^{298\text{K}}_{\text{reactants}} = \\ &(-851233.4 - 431528.5 - 155928.8) - (-717854.2 - 431873.3 - 288917.5) = -45.7\ \text{kcal/mol}\end{aligned} \tag{2}$$

Transition state calculation were done with the QST3 method[24,25] with the same func-

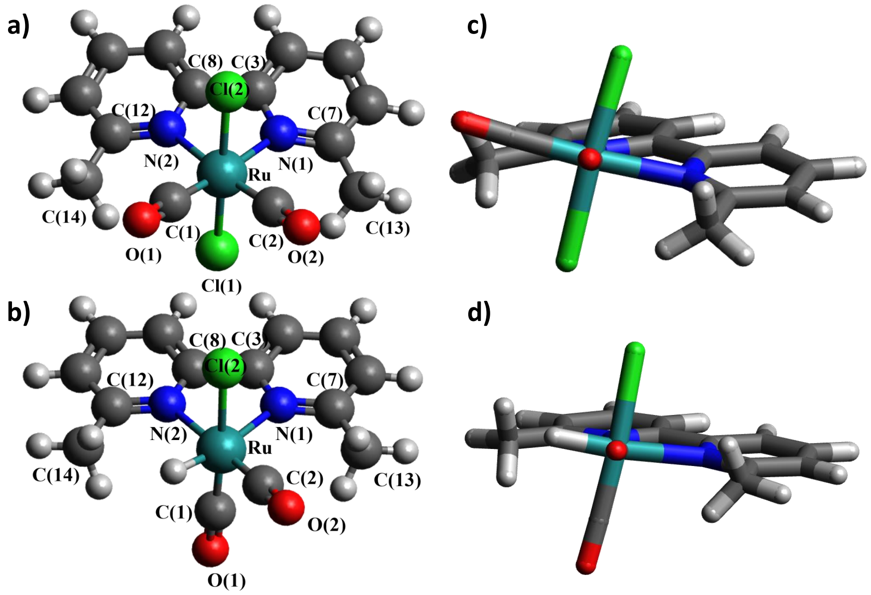

Figure S19: (a) Calculated structures of the initial catalyst and (b) the final product, and in panels (c) and (d) as stick representations viewed from the C(2)O(2) carbonyl. The atom labels in (a) follow the labeling in the Ref.[5] to facilitate comparison.

tional/basis set combination and solvation model as described above. In order to evaluate the accuracy of the calculated structures, we present a comparison between our computational results for the catalyst, Ru6dmb, and existing literature reports (see Tables S2 and S3). The structure of the catalyst has been studied before both experimentally, by X-ray diffraction, and theoretically. The latter figure also illustrates the increase in planarity of the bpy ring system after the formation of the final active catalyst (see Fig. S19b and d).

Table S4 summarizes the two CO vibrational frequencies of all relevant species, as well as their electronic energies. In the cases when two conformers (awm and aam) are present, the average reaction energy is reported. For example, the following reaction of ligand exchange: has two possible reactants and products, so a total of 4 combinations may be considered, but their average energy is 6.4 kcal/mol.

$$[\mathrm{Ru^I(dmbpy)(CO)_2Cl}]^{\bullet} + \mathrm{DMF} \rightleftharpoons [\mathrm{Ru^I(dmbpy)(CO)_2DMF}]^{\bullet +} + \mathrm{Cl}^{-} \qquad (3)$$

The table serves as a guidance to identify certain transient intermediates, based on the

Table S2: Comparison between our computational results and the literature reports of certain bond lengths in the Ru6dmb (Å). Atom labels are defined in Fig. S19a.

Selected Bonds	DFT Calculation	Ref.[5] Exp.	Ref.[5] DFT	Ref.[26] Exp.
Ru-Cl(1)	2.414	2.396	2.475	2.397
Ru-Cl(2)	2.418	2.387	2.483	2.390
Ru-N(1)	2.183	2.147	2.200	2.158
Ru-N(2)	2.182	2.150	2.199	2.155
Ru-C(1)	1.866	1.879	1.901	1.873
Ru-C(2)	1.866	1.875	1.902	1.872
C(1)-O(1)	1.136	1.129	1.149	1.131
C(2)-O(2)	1.136	1.139	1.149	1.138
C(7)-C(13)	1.495	1.498	1.504	1.498
C(12)-C(14)	1.494	1.498	1.503	1.484

Table S3: Comparison between our computational results and the literature reports of selected bond angles of the Ru6dmb (°). Atom labels are defined in Fig. S19a.

Selected Bond Angles	DFT Calculation	Ref.[26] Exp.
C(2)-Ru-C(1)	83.1	82.3
Cl(2)-Ru-Cl(1)	175.01	174.39
N(1)-Ru-N(2)	76.23	77.27
N(1)-C(7)-C(13)	120.2	120.4
N(2)-C(12)-C(14)	120.2	119.5
N(1)-Ru-Cl(1)	89.93	91.07
N(1)-Ru-Cl(2)	86.16	85.12
N(2)-Ru-Cl(1)	89.62	89.35
N(2)-Ru-Cl(2)	86.43	85.79
C(1)-Ru-Cl(1)	93.7	93.0
C(1)-Ru-Cl(2)	90.1	90.7
C(2)-Ru-Cl(1)	93.5	93.4
C(2)-Ru-Cl(2)	90.2	91.3

match of the CO vibrational frequencies as well as the criterion whether they are energetically feasible.

Table S4: The CO vibrational frequencies in cm^{-1} (IR intensities in brackets in km/mol) and Gibbs free energies (kcal/mol) of the relevant species calculated by DFT. For the hydride species, the Ru-H vibration frequency is shown following the COs. Section 1 lists the considered Ru^{II}-species and sec. 2 the Ru^{I}-species, followed by the extras needed for reaction energies (sec. 3). Sec. 4, separated by a double horizontal line, shows the three catalyst^{-} variants (solvated in DMSO via PCM). The last Section 5, separated by a triple line, lists the two Ru5dmb isomers. All species are solvated in DMF (PCM), unless otherwise stated. (awm) and (aam) identify the ligand alignment with the directions of dmbpy methyl substituents or against them, respectively. (Equat.) superscript is used to identify the ligand which is in the equatorial position with respect to the dmbpy pseudo-plane. (iso) stands for a carbonyl-isomerized species where one carbonyl is axial and the other is equatorial, whereas (iso)‡ is the TS of the rearrangement.

**CO stretch (as/s) / Ru-H stretch**	**Gibbs Free Energy**	
Ru^{II}(6,6'-dmbpy)$(CO)_2Cl_2$	1990(1305)/2056(1515)	-1139682.9
Ru^{II}(6,6'-dmbpy$^{\bullet -}$)$(CO)_2Cl_2$	1959(1353)/2030(1804)	-1139750.5
(awm)-[Ru^{II}(6,6'-dmbpy)$(CO)_2Cl]^{+}$	2022(1233)/2082(1425)	-850739.4
(aam)-[Ru^{II}(6,6'-dmbpy)$(CO)_2Cl]^{+}$	2022(1235)/2082(1405)	-850739.6
(awm)-[Ru^{II}(6,6'-dmbpy)$(CO)_2$DMF$]^{2+}$	2038(1145)/2093(1233)	-717734.6
(aam)-[Ru^{II}(6,6'-dmbpy)$(CO)_2$DMF$]^{2+}$	2035(1131)/2091(1236)	-717734.8
Ru^{II}(6,6'-dmbpy)$(CO)_2$Cl $H^{(Equat.)}$	1930(1908)/2010(1181) 2027(720)	-851233.4
[Ru^{II}(6,6'-dmbpy)$(CO)_2$DMF $H^{(Equat.)}]^{+}$	1948(1848)/2024(1040) 2041(792)	-718239.8
(awm)-[Ru^{I}(6,6'-dmbpy)$(CO)_2Cl]^{\bullet}$	1906(1930)/1990(1891)	-850845.3
(aam)-[Ru^{I}(6,6'-dmbpy)$(CO)_2Cl]^{\bullet}$	1910(1861)/1992(1930)	-850843.7
(awm)-[Ru^{I}(6,6'-dmbpy)$(CO)_2$DMF$]^{\bullet +}$	1924(1880)/2009(1694)	-717849.5
(aam)-[Ru^{I}(6,6'-dmbpy)$(CO)_2$DMF$]^{\bullet +}$	1927(1858)/2010(1720)	-717849.4
(iso)-[Ru^{I}(6,6'-dmbpy)$(CO)_2Cl]^{\bullet}$	1899(2168)/1990(1738)	-850850.3
(iso)‡-[Ru^{I}(6,6'-dmbpy)$(CO)_2Cl]^{\bullet}$	1886(1986)/1994(1707)	-850840.6
(iso)-[Ru^{I}(6,6'-dmbpy)$(CO)_2$DMF$]^{\bullet +}$	1915(2118)/2005(1598)	-717854.2
(iso)‡-[Ru^{I}(6,6'-dmbpy)$(CO)_2$DMF$]^{\bullet +}$	1906(1883)/2010(1544)	-717843.7
[Ru^{I}(6,6'-dmbpy)$(CO)_2Cl^{(awm)}$DMF$]^{\bullet}$	1971(1308)/2041(1753)	-1006756.3
[Ru^{I}(6,6'-dmbpy)$(CO)_2$Cl $DMF^{(awm)}]^{\bullet}$	1970(1311)/2040(1753)	-1006755.3
DMF		-155928.8
Cl^{-}		-288917.5
$BNAH^{\bullet +}$		-431873.3
BNA^{+}		-431528.5
[Ru^{II}(4,4'-dmbpy$^{\bullet -}$)$(CO)_2Cl_2]^{-}$	1957(1730)/2030(1678)	-1139757.9
[Ru^{II}(5,5'-dmbpy$^{\bullet -}$)$(CO)_2Cl_2]^{-}$	1955(1700)/2028(1687)	-1139755.0
[Ru^{II}(6,6'-dmbpy$^{\bullet -}$)$(CO)_2Cl_2]^{-}$	1958(1356)/2030(1821)	-1139750.3
trans(Cl)-[Ru^{II}((5,5'-dmbpy)$(CO)_2Cl_2$]	1988(1574)/2055(1413)	-1139687.7
cis(Cl)-[Ru^{II}((5,5'-dmbpy)$(CO)_2Cl_2$]	1990(1584)/2059(1320)	-1139689.6

7.1 Cartesian Coordinates

Coordinates of the reported intermediates are listed in Tables S5 to S28.

Table S5: Coordinates for $Ru^{II}(dmbpy)(CO)_2Cl_2$

C	0.72961300	1.94237500	0.05524100
C	1.45153300	3.10709500	0.23929800
N	1.33813700	0.76912600	-0.21322700
C	-0.75176200	1.93886300	0.04592900
C	2.82545400	3.07771500	0.09361200
C	2.66325600	0.74456900	-0.42083000
C	-1.47617800	3.11122600	0.16169700
N	-1.35573900	0.75361200	-0.17560800
C	3.42826500	1.89580900	-0.27100000
C	-2.84960000	3.07203400	0.01793600
C	-2.68262800	0.71516900	-0.37304600
C	-3.45075200	1.86968600	-0.27581800
H	0.95998100	4.03472800	0.48293800
H	3.40963300	3.97642800	0.23780700
C	3.33719800	-0.52674200	-0.82576700
H	-0.98561200	4.05350800	0.34261500
H	-3.43497000	3.97689800	0.10888100
C	-3.35522400	-0.57675500	-0.70766100
Ru	0.00422400	-0.93354100	0.08121300
Cl	-0.03746900	-1.30961200	-2.30315900
Cl	0.04285700	-0.35026700	2.42799700
C	-1.21681400	-2.29875200	0.43837000
O	-1.89486300	-3.17304100	0.69589700
C	1.25748200	-2.28210300	0.38775800
O	1.95863500	-3.14657900	0.61443500
H	4.49422000	1.84355700	-0.44061300
H	-4.51748600	1.80553600	-0.43571100
H	2.69728300	-1.12044600	-1.47557600
H	3.60381300	-1.11625700	0.05251900
H	4.26023500	-0.29930900	-1.35545800
H	-2.74386500	-1.17286600	-1.38264600
H	-4.31355400	-0.38051400	-1.18399200
H	-3.55312800	-1.15081300	0.19850000

Table S6: Coordinates for $Ru^{II}(6dmbpy^{\bullet -})(CO)_2Cl_2$

C	0.70670700	1.95021400	0.05903400
C	1.47253100	3.13373400	0.21264200
N	1.34818900	0.74289400	-0.15455200
C	-0.70796900	1.94969500	0.05932600
C	2.82218900	3.10245800	0.04939800
C	2.68207400	0.73905600	-0.36920300
C	-1.47469600	3.13246600	0.21427200
N	-1.34859300	0.74202000	-0.15486100
C	3.44159600	1.88550600	-0.28882600
C	-2.82433800	3.10028100	0.05109100
C	-2.68243400	0.73739700	-0.36983100
C	-3.44278200	1.88323300	-0.28856500
H	0.98240400	4.06755600	0.44243000
H	3.40906500	4.00577000	0.15872100
C	3.36824800	-0.55118100	-0.70144700
H	-0.98533100	4.06638100	0.44532500
H	-3.41194600	4.00297200	0.16160400
C	-3.36762900	-0.55295600	-0.70360600
Ru	0.00030600	-0.92426400	0.07680100
Cl	0.00082700	-1.24709600	-2.33374900
Cl	-0.00017100	-0.44026200	2.46410400
C	-1.23251000	-2.29679500	0.35826600
O	-1.90370300	-3.19269200	0.57277900
C	1.23383300	-2.29609600	0.35867200
O	1.90540900	-3.19163800	0.57347900
H	4.50489500	1.83315100	-0.47400100
H	-4.50600700	1.83028700	-0.47400400
H	2.78781100	-1.13329700	-1.41650100
H	3.52115800	-1.15188700	0.19604900
H	4.34888300	-0.34975600	-1.12912500
H	-2.78600000	-1.13445400	-1.41819300
H	-4.34778300	-0.35172200	-1.13247100
H	-3.52152600	-1.15414300	0.19338200

Table S7: Coordinates for (awm)-$[Ru^{II}(dmbpy)(CO)_2Cl]^+$

C	-0.74435000	1.91759000	-0.19995100
C	-1.46987400	3.08712800	-0.31194900
N	-1.34819900	0.72973800	0.02808300
C	0.73781200	1.91648900	-0.21423800
C	-2.84241300	3.04461900	-0.14999100
C	-2.67358100	0.68759700	0.24401600
C	1.46788300	3.07352100	-0.39936400
N	1.34042600	0.73656000	0.05600500
C	-3.43988800	1.84379500	0.15663200
C	2.84104900	3.03359900	-0.23842700
C	2.66489000	0.70086200	0.27630000
C	3.43409600	1.85026500	0.13724900
H	-0.98223300	4.02959900	-0.50004200
H	0.98461500	4.00343100	-0.65108500
Ru	0.00450100	-0.91697300	-0.33616800
C	1.27170000	-2.26202300	-0.71989000
O	1.97244800	-3.11903800	-0.95410500
C	-1.23879800	-2.27359800	-0.76063100
O	-1.92199900	-3.13853200	-1.01643300
Cl	-0.02761500	-1.55963200	1.89521100
C	-3.34243900	-0.60397500	0.58612500
H	-4.50395200	1.78131100	0.33318800
C	3.32849700	-0.57603900	0.67890600
H	4.49741100	1.79455600	0.32097500
H	-3.61693000	-1.14404700	-0.32133600
H	-4.26161100	-0.40485700	1.13307600
H	-2.70253100	-1.23319800	1.20064600
H	3.65003000	-1.13371300	-0.20214200
H	2.66739900	-1.19766800	1.27835000
H	4.22002900	-0.35413000	1.26205400
H	3.43226600	3.92816800	-0.37862300
H	-3.42975600	3.94859700	-0.23529500

Table S8: Coordinates for (aam)-$[Ru^{II}(dmbpy)(CO)_2Cl]^+$

C	0.71854200	1.83052700	0.06383900
C	1.44026800	2.96341300	0.38298600
N	1.32928900	0.69901600	-0.34991200
C	-0.76185400	1.81964300	0.05249700
C	2.81439200	2.95195700	0.22493700
C	2.65261800	0.70043400	-0.57191200
C	-1.49963500	2.95619100	0.31811300
N	-1.35372400	0.66729600	-0.33075800
C	3.41693000	1.82553600	-0.28627600
C	-2.87284000	2.92007300	0.15940800
C	-2.67794800	0.64125800	-0.54826300
C	-3.45919800	1.76361800	-0.30073200
H	0.94983100	3.85537500	0.73735900
H	3.39875100	3.82788200	0.47138800
C	3.31991300	-0.50791600	-1.14551800
H	-1.02061000	3.87130100	0.62562500
H	-3.46914000	3.79821800	0.36614500
C	-3.32931800	-0.59692900	-1.07367200
Ru	0.00825700	-1.01638700	-0.24743600
Cl	0.02258800	-0.76185100	2.06552200
C	-1.22995400	-2.42204900	-0.00352300
O	-1.90828600	-3.29986700	0.21892500
C	1.28834600	-2.38684700	-0.02247700
O	1.99568800	-3.24353600	0.19179300
H	4.48114400	1.79823900	-0.47151600
H	-4.52355300	1.71201900	-0.47955000
H	2.66427800	-1.04256000	-1.82975600
H	3.64091700	-1.18402800	-0.35193300
H	4.21139900	-0.20776000	-1.69253400
H	-2.68203700	-1.12554400	-1.77037100
H	-4.24840800	-0.33548600	-1.59383300
H	-3.59960600	-1.26555000	-0.25522500

Table S9: Coordinates for (awm)-$[Ru^{II}(dmbpy)(CO)_2DMF]^{2+}$

C	-2.28326100	0.82303500	0.28397100
C	-3.36490800	1.61157500	0.61682600
N	-1.06981500	1.36237900	0.02606400
C	-2.34647200	-0.65581900	0.31530600
C	-3.18593700	2.97789200	0.74161100
C	-0.86832300	2.67637300	0.21656200
C	-3.49493000	-1.33810900	0.65998600
N	-1.17910600	-1.30378300	0.09623600
C	-1.92533300	3.50397600	0.57524000
C	-3.43050700	-2.70831800	0.84116900
C	-1.09042400	-2.62183100	0.33716000
C	-2.21635200	-3.34379200	0.71376300
H	-4.33322100	1.17681100	0.80317000
H	-4.42865800	-0.82004400	0.80880700
Ru	0.19615300	-0.04622300	-0.95470500
C	1.16019800	-1.39167200	-1.90797000
O	1.73802200	-2.13822600	-2.52681800
C	1.23333700	1.18379800	-1.98400400
O	1.84246200	1.86395600	-2.64748300
H	3.07868600	1.05264700	3.55392300
H	5.20757600	0.80129700	2.21608900
C	2.66387300	-0.09281500	0.68000900
O	1.40161200	-0.02497500	0.65280100
C	2.69062800	0.15062200	3.08232600
N	3.33838000	-0.02074700	1.78976600
C	4.79145300	-0.11190200	1.79250700
H	5.15964400	-0.24113900	0.77821800
H	1.61759400	0.23582800	2.95016600
H	2.91901700	-0.70943500	3.71103400
H	5.09664900	-0.96282600	2.40016800
H	3.24301800	-0.21611900	-0.23408500
C	0.49842100	3.26016100	0.06095700
H	-1.73876900	4.55827500	0.72005500
C	0.22413400	-3.32151400	0.21434400
H	-2.12128500	-4.40359200	0.90113400
H	1.27233200	2.54520900	0.32804900
H	0.59800100	4.12858600	0.70923700
H	0.65777900	3.60397500	-0.96203000
H	1.05136900	-2.67109200	0.48739600
H	0.37201600	-3.68968300	-0.80175700
H	0.23926800	-4.18689300	0.87360000
H	-4.31801500	-3.26377100	1.11161900
H	-4.02030100	3.61483800	1.00119200

Table S10: Coordinates for (aam)-$[Ru^{II}(dmbpy)(CO)_2DMF]^{2+}$

C	0.30428700	2.00588700	0.19156300
C	0.04661800	3.20689900	0.81851800
N	-0.55892100	1.48009400	-0.70720300
C	1.58159000	1.28018200	0.36721100
C	-1.09574300	3.91032700	0.47731000
C	-1.62738800	2.18878300	-1.10069700
C	2.60435100	1.76731500	1.15410900
N	1.73654400	0.16257600	-0.37710800
C	-1.91439100	3.41517000	-0.51129100
C	3.82741900	1.11943800	1.14117100
C	2.93748900	-0.43015900	-0.45285400
C	4.00181700	0.03781500	0.30884700
H	0.72762700	3.61077300	1.55008400
H	2.47103300	2.65081900	1.75734500
Ru	-0.15594100	-0.60503100	-1.03100100
C	0.28490200	-2.45882700	-1.13428500
O	0.46787900	-3.57226400	-1.16772200
C	-1.91430200	-1.18263500	-1.48811500
O	-2.94037000	-1.59239300	-1.72147800
C	-2.51191000	1.67145800	-2.18883400
H	-2.78373300	3.96551700	-0.84093300
C	3.14416800	-1.59378000	-1.36781400
H	4.95836500	-0.45910500	0.23438300
H	-1.95505400	1.08298700	-2.91582800
H	-2.97314100	2.50665400	-2.71211500
H	-3.32075700	1.06745900	-1.77513200
H	2.52510200	-1.52282600	-2.26011200
H	2.93113200	-2.53115300	-0.85226200
H	4.18537300	-1.63068900	-1.68150300
H	4.64216800	1.48163700	1.75294900
H	-1.31909500	4.85211100	0.95966800
O	-0.46972600	-0.70599100	0.95506400
C	-1.26586700	-1.43557600	1.60900300
H	-1.86812400	-2.20104400	1.12274600
N	-1.40617700	-1.31341100	2.89694000
C	-2.29098400	-2.19741800	3.64337800
C	-0.65662300	-0.32212400	3.65793600
H	-2.73674300	-2.93070300	2.97486100
H	-3.07740700	-1.60856700	4.11567800
H	-1.71563300	-2.71427400	4.41114200
H	-0.29551100	0.45891800	2.99475800
H	0.18826800	-0.80184400	4.15410200
H	-1.31746400	0.10852900	4.40797700

Table S11: Coordinates for $Ru^{II}(dmbpy)(CO)_2Cl\ H^{(Equat.)}$

C	-1.57144000	1.19535900	-0.06385300
C	-2.77012400	1.88810000	-0.01147800
N	-1.54015700	-0.15124800	-0.10486500
C	-0.27164000	1.91443200	-0.10513700
C	-3.95836200	1.18599700	-0.01306300
C	-2.69225000	-0.84317100	-0.12489600
C	-0.21259700	3.29958500	-0.08288500
N	0.84043800	1.16613000	-0.19035000
C	-3.91699200	-0.18878800	-0.07885700
C	1.01690800	3.92241700	-0.16546000
C	2.03706400	1.76221900	-0.28681200
C	2.15031000	3.14700400	-0.27764500
H	-2.79027300	2.96466000	0.02600400
H	-1.10532400	3.89809300	-0.00590100
Ru	0.45532600	-1.04305500	0.03002000
C	2.08158800	-1.88744200	0.28273300
O	3.04572400	-2.47306000	0.46048200
C	0.49448300	-1.47682000	-1.75859200
O	0.50768700	-1.77628400	-2.86269800
Cl	0.30845200	-0.59200400	2.45799000
C	3.26567700	0.91827200	-0.39965700
H	3.12821300	3.60007700	-0.35695500
C	-2.66816400	-2.33704300	-0.19425800
H	-4.82705800	-0.77094400	-0.09420600
H	-3.68006700	-2.72058200	-0.30684700
H	-2.23195800	-2.75406900	0.71318000
H	-2.06410300	-2.67946000	-1.03297000
H	4.13363200	1.53813800	-0.61425200
H	3.15700600	0.17981400	-1.19324800
H	3.44858300	0.38808100	0.53572900
H	-4.90343900	1.71043500	0.02903300
H	1.08402400	5.00190100	-0.14928700
H	-0.06327300	-2.50782400	0.30639900

Table S12: Coordinates for $[Ru^{II}(dmbpy)(CO)_2DMF\ H^{(Equat.)}]^+$

C	-0.74129000	1.89365400	-0.36920200
C	-1.08508400	3.04280800	-1.06217200
N	-1.48323300	0.77584700	-0.45215500
C	0.43436300	1.85771900	0.53854200
C	-2.22553600	3.04120500	-1.84186800
C	-2.60273600	0.77077400	-1.18888000
C	1.26651600	2.95413200	0.68265200
N	0.63574500	0.72692500	1.24574600
C	-2.99602200	1.90037100	-1.89612300
C	2.30497900	2.89864800	1.59143800
C	1.62373500	0.67359300	2.15473900
C	2.47186900	1.75763700	2.34409100
H	-0.48810200	3.93747300	-0.99530800
H	1.11472300	3.84896400	0.10175500
Ru	-0.51129800	-0.97609700	0.53326100
C	-1.57498600	-1.11015800	2.02778000
O	-2.20972000	-1.20813400	2.97200300
C	-1.30480400	-2.50990400	-0.15146900
O	-1.73601300	-3.49347700	-0.53416300
H	1.77860700	0.69515600	-2.70582200
H	4.09664100	-2.17278200	-3.35756400
C	1.90390100	-1.37263000	-1.28184200
O	0.82746200	-0.76167400	-1.13344800
C	2.63109800	0.11314700	-3.04291100
N	2.81231200	-1.03172300	-2.16907900
C	4.03118700	-1.80269600	-2.33415800
H	4.03151700	-2.64852500	-1.64994000
H	3.53101400	0.72713300	-3.01543800
H	2.46218700	-0.22318400	-4.06643800
H	4.89836700	-1.17362300	-2.12730000
H	2.14909900	-2.23953700	-0.66421700
H	-2.51251800	3.92857000	-2.38976800
H	2.96667100	3.74504400	1.71625600
C	-3.43216900	-0.47133100	-1.24881200
H	-2.93013500	-1.23486600	-1.84453400
H	-3.60201300	-0.87502400	-0.25158900
H	-4.39428900	-0.26234100	-1.71138200
C	1.82261500	-0.56319500	2.97184700
H	0.88261000	-0.90809700	3.39841300
H	2.21752600	-1.36980200	2.35392600
H	2.52584500	-0.36652600	3.77827400
H	-3.90404400	1.87040200	-2.48114400
H	3.25950000	1.68896900	3.08041200
H	0.44048200	-2.05285000	1.19927300

Table S13: Coordinates for (awm)-$[Ru^{I}(dmbpy)(CO)_2Cl]^{\bullet}$

C	-0.72806200	1.91829300	-0.23717600
C	-1.45016700	3.07962600	-0.44403500
N	-1.33925500	0.74985300	0.05017500
C	0.75651000	1.91077900	-0.21983300
C	-2.82468800	3.05208000	-0.29341400
C	-2.66346800	0.72930900	0.26204300
C	1.48756700	3.08011600	-0.32832000
N	1.35473000	0.72243600	0.00571400
C	-3.42923100	1.87784000	0.09570000
C	2.85983500	3.03332900	-0.16664400
C	2.67871600	0.67743200	0.21946500
C	3.45378700	1.82839900	0.13464200
H	-0.95937700	4.00269000	-0.70730700
H	1.00355200	4.02527000	-0.51376000
Ru	-0.01266600	-0.97209400	-0.28983900
C	1.18967200	-2.22976900	-0.91688800
O	1.88707900	-3.03800400	-1.33510200
C	-1.26661800	-2.22579000	-0.82097900
O	-1.99740200	-3.03418900	-1.17613700
Cl	0.04616400	-1.55165900	2.25093100
C	-3.31922600	-0.54380400	0.69089300
H	-4.49489900	1.83113700	0.27015000
C	3.32631200	-0.62572300	0.56068600
H	4.51811900	1.76090400	0.30985800
H	-3.62495400	-1.12823900	-0.17841400
H	-4.21564000	-0.32156100	1.26744500
H	-2.64134600	-1.14309600	1.29648600
H	3.57332200	-1.17804700	-0.34737200
H	2.66562300	-1.23658600	1.17379100
H	4.25534600	-0.44800100	1.09936700
H	3.44996000	3.93608700	-0.24856200
H	-3.40855500	3.94857400	-0.45274400

Table S14: Coordinates for (aam)-[Ru^{I}(dmbpy)$(CO)_2$Cl]$^{\bullet}$

C	0.79440000	1.80921800	-0.04014300
C	1.54115500	2.95162700	0.18626100
N	1.37526800	0.63771100	-0.37051300
C	-0.68832400	1.83976300	-0.02594300
C	2.91457700	2.89822700	0.04403200
C	2.70222600	0.59365800	-0.56639800
C	-1.38839900	2.98919400	0.29283800
N	-1.31880400	0.71312600	-0.41617200
C	3.49465700	1.71632700	-0.35842700
C	-2.76424200	2.99789700	0.15934700
C	-2.64600100	0.73474900	-0.60935800
C	-3.39207900	1.87286700	-0.32591000
H	1.06854700	3.88285500	0.45280100
H	3.51697300	3.77911000	0.22039900
C	3.34262600	-0.67590200	-1.02703900
H	-0.87989400	3.87589000	0.63487300
H	-3.33144200	3.88470100	0.40797900
C	-3.33658100	-0.47660400	-1.14833400
Ru	-0.02080900	-1.06293400	-0.18313500
Cl	-0.07050100	-0.51492200	2.38057800
C	-1.27879900	-2.41747700	-0.10010300
O	-2.00258800	-3.30100000	-0.00680000
C	1.15533500	-2.48178300	-0.02287200
O	1.82319000	-3.40192100	0.12044000
H	4.56082300	1.64681600	-0.52075800
H	-4.46048300	1.85754200	-0.48837000
H	2.70975600	-1.19530800	-1.74445900
H	3.52867200	-1.34098100	-0.18290400
H	4.30122800	-0.46015100	-1.49489200
H	-2.69858200	-1.01213200	-1.84875700
H	-4.25263400	-0.18424100	-1.65844200
H	-3.61220100	-1.15440700	-0.33940100

Table S15: Coordinates for (awm)-$[Ru^{I}(dmbpy)(CO)_2DMF]^{\bullet+}$

C	-2.29844200	-0.73885600	-0.47191700
C	-3.36730700	-1.46834800	-0.95782500
N	-1.15765500	-1.33974600	-0.07298500
C	-2.29904200	0.74563500	-0.47177600
C	-3.24600100	-2.84155100	-1.07223500
C	-1.00952200	-2.66229000	-0.23791000
C	-3.40252900	1.48045000	-0.86215200
N	-1.12797700	1.34284000	-0.16208800
C	-2.05056700	-3.43682000	-0.73701600
C	-3.28113100	2.85220400	-0.99725000
C	-0.98284400	2.66092100	-0.35796600
C	-2.05483000	3.43931500	-0.77959700
H	-4.27822100	-0.98336000	-1.26963900
H	-4.34541400	1.00188300	-1.07319900
Ru	0.14195900	0.02278100	1.03829600
C	1.00192400	1.28820000	2.09139400
O	1.53828700	2.02403400	2.78381100
C	0.89206800	-1.18087100	2.23572800
O	1.34916300	-1.87691500	3.02039600
H	3.48581700	-1.14428900	-3.60363000
H	5.53422900	-0.83490200	-2.09086500
C	2.82040000	-0.01688200	-0.75989400
O	1.58708500	-0.11408100	-0.79948400
C	3.07672000	-0.23596000	-3.15873400
N	3.60945100	-0.06317700	-1.82129800
C	5.04865800	0.05985900	-1.69857900
H	5.32325700	0.18255300	-0.65311000
H	1.99493700	-0.31072000	-3.10876100
H	3.35604900	0.61653700	-3.77992800
H	5.39783200	0.92623800	-2.26244400
H	3.34873100	0.11861900	0.18992700
C	0.29418800	-3.30627500	0.11053100
H	-1.90580000	-4.50103700	-0.85813000
C	0.35210800	3.29503100	-0.13064400
H	-1.91123400	4.50047600	-0.92602600
H	1.12621100	-2.63407000	-0.08862300
H	0.42628200	-4.21692900	-0.47092100
H	0.31299800	-3.58452300	1.16554900
H	1.15755600	2.58934200	-0.32130300
H	0.43630500	3.65667900	0.89530300
H	0.47111900	4.15337600	-0.78972200
H	-4.13261200	3.44717300	-1.29863700
H	-4.07034500	-3.43169300	-1.44900600

Table S16: Coordinates for (aam)-$[Ru^{I}(dmbpy)(CO)_2DMF]^{\bullet+}$

C	-1.85639800	-0.66202500	0.67662600
C	-2.62320500	-1.35596000	1.59404300
N	-1.21846100	-1.29299100	-0.33147000
C	-1.78315900	0.81942900	0.68071600
C	-2.77427300	-2.72268900	1.44646900
C	-1.41121000	-2.60716800	-0.51750600
C	-2.45065800	1.58059300	1.62178200
N	-1.11118700	1.39017000	-0.34150000
C	-2.18866100	-3.34553500	0.36720300
C	-2.47255500	2.95623200	1.48045600
C	-1.18302200	2.71707900	-0.52179500
C	-1.86281300	3.52360000	0.38404400
H	-3.11979800	-0.84761900	2.40466700
H	-2.96479400	1.11962400	2.44958200
Ru	0.32515900	-0.01497100	-1.22280600
C	1.58757300	1.16847600	-1.90097200
O	2.41688500	1.84501900	-2.30419300
C	1.49509100	-1.29932000	-1.88104100
O	2.27153500	-2.04154500	-2.27387000
C	-0.79612600	-3.28591800	-1.69927700
H	-2.32625100	-4.40311000	0.19264600
C	-0.53700100	3.33733300	-1.71917100
H	-1.90341300	4.59007500	0.21402000
H	-0.75076600	-2.61750200	-2.55704000
H	-1.38190600	-4.16309300	-1.96806400
H	0.21446800	-3.62455700	-1.46626900
H	-0.57667900	2.66984500	-2.57818600
H	0.50649800	3.57971600	-1.51227400
H	-1.04489100	4.26615700	-1.97308800
H	-2.98827700	3.57076300	2.20600900
H	-3.37002900	-3.28490900	2.15277100
O	1.09077800	-0.04252700	1.03738600
C	2.27501500	-0.09474300	1.38831700
H	3.08520900	-0.12785600	0.65162600
N	2.68792700	-0.11743800	2.64681700
C	4.09850300	-0.18199500	2.97692900
C	1.75394700	-0.08108000	3.75545400
H	4.69272200	-0.20443700	2.06572800
H	4.30540700	-1.08210400	3.55765700
H	4.38499200	0.69039900	3.56611300
H	0.73954300	-0.03400200	3.37045100
H	1.95093600	0.79493700	4.37550900
H	1.87221600	-0.97721000	4.36642300

Table S17: Coordinates for (iso)-[Ru^{I}(dmbpy)$(CO)_2$Cl]$^{\bullet}$

C	1.58461700	1.20748600	0.05661900
C	2.81031400	1.85384300	0.00992800
N	1.51145300	-0.13287200	0.09543500
C	0.30155500	1.95965800	0.05966100
C	3.96976300	1.10329200	0.00897900
C	2.62994500	-0.87328500	0.09165100
C	0.27769400	3.34626300	0.08299200
N	-0.83082300	1.23992400	0.03994500
C	3.87972000	-0.27244400	0.05131200
C	-0.94087000	3.99672900	0.07785200
C	-2.01643000	1.86076400	0.03447200
C	-2.09833300	3.24810400	0.05099400
H	2.87229400	2.92913700	-0.03088400
H	1.18853800	3.92242600	0.10810700
Ru	-0.46962300	-1.04407700	0.04214400
C	-2.09288500	-1.93635200	-0.07322400
O	-3.08661600	-2.50124500	-0.14758700
C	-0.50481400	-1.29241400	1.86865800
O	-0.52666100	-1.44994100	3.00559200
Cl	-0.22092100	-1.05923400	-2.41284800
C	-3.26021700	1.03199900	0.00933400
H	-3.06849800	3.72457200	0.04428500
C	2.49512300	-2.36222800	0.12335200
H	4.76685600	-0.88945200	0.04922600
H	3.47344900	-2.83780800	0.10309900
H	1.91855400	-2.70704200	-0.73721400
H	1.96933500	-2.68143700	1.02464700
H	-4.14418300	1.66620900	0.01096100
H	-3.29777500	0.37658800	0.87988600
H	-3.28090800	0.40552300	-0.88272900
H	4.93421600	1.59176700	-0.02768300
H	-0.98210600	5.07755300	0.09519200

Table S18: Coordinates for (iso)‡-[Ru^{I}(dmbpy)(CO)$_2$Cl]$^{\bullet}$

C	-0.87637700	1.82746800	-0.07324000
C	-1.70393900	2.93245600	0.03147300
N	-1.36874600	0.57280000	-0.05998400
C	0.59725600	1.98632900	-0.15304300
C	-3.06854000	2.74629200	0.13566200
C	-2.69538200	0.38158200	0.04791500
C	1.17931000	3.21395700	-0.42428000
N	1.34052400	0.89040500	0.07859200
C	-3.56461100	1.46160600	0.14254300
C	2.55735600	3.31933900	-0.40593100
C	2.67647200	0.99146900	0.12965000
C	3.31107500	2.20490800	-0.10357200
H	-1.29396700	3.92932300	0.05503200
H	0.57716300	4.07858300	-0.65453500
Ru	0.10617100	-1.04074600	-0.13874100
C	1.34269000	-2.42215700	-0.20671800
O	2.05623500	-3.31469700	-0.24612700
C	-0.65592200	-1.85082600	-1.58474400
O	-1.12157300	-2.35372100	-2.51199200
Cl	-0.42355600	-1.53639400	2.29091400
C	-3.24506600	-1.00906200	0.05556600
C	3.47946200	-0.22980500	0.44342400
H	4.38936200	2.25979200	-0.05379400
H	-4.62577800	1.27691700	0.22910100
H	-3.19159700	-1.44444600	-0.94341600
H	-4.28937500	-0.99619700	0.36058300
H	-2.67870500	-1.64472100	0.73392800
H	3.55218200	-0.87171400	-0.43612800
H	3.01571700	-0.80061500	1.24700800
H	4.48898000	0.04431800	0.74287500
H	-3.73171700	3.59651800	0.22075200
H	3.03420300	4.26734200	-0.61589300

Table S19: Coordinates for (iso)-$[Ru^{I}(dmbpy)(CO)_2DMF]^{+}$

C	1.81602900	0.72745300	1.04464300
C	2.78245400	0.99743900	2.00127000
N	1.51093200	-0.53142400	0.69285100
C	1.07944300	1.82241300	0.35842700
C	3.43332500	-0.05683300	2.61290900
C	2.13435400	-1.55963500	1.28030300
C	1.22566200	3.14789200	0.73494800
N	0.25762700	1.47602100	-0.64697100
C	3.10394800	-1.34614700	2.25305200
C	0.52564400	4.12414800	0.05278800
C	-0.42612200	2.41477600	-1.31907100
C	-0.30260100	3.75597900	-0.98664800
H	3.03926000	2.00996600	2.26818200
H	1.86862200	3.42695900	1.55379100
Ru	-0.07389500	-0.64711900	-0.97363600
C	1.05028200	-0.74338000	-2.42611600
O	1.75731100	-0.80155100	-3.32584500
C	-0.52151100	-2.42652900	-1.28820400
O	-0.80217900	-3.51590100	-1.49627500
H	-1.94768900	0.57438300	2.74609300
H	-5.17316800	-1.26485100	2.44254800
C	-2.69249900	-0.61073900	0.66341300
O	-1.47086000	-0.37291000	0.65961700
C	-3.00955900	0.37496100	2.84966600
N	-3.49506000	-0.29711900	1.65843600
C	-4.91129700	-0.60996800	1.61090200
H	-5.15027600	-1.10993400	0.67508400
H	-3.54853200	1.31379700	2.98126800
H	-3.18096100	-0.25685600	3.72197400
H	-5.49509200	0.30809500	1.68520100
C	-1.32953500	1.98366700	-2.43117300
C	1.76772800	-2.95022400	0.87247900
H	-3.17644200	-1.10423200	-0.18323400
H	4.19121300	0.13100500	3.36152900
H	0.62770500	5.16361000	0.33397700
H	-0.86128300	4.49529500	-1.54233700
H	3.59189200	-2.19340900	2.71368400
H	2.35668600	-3.67973500	1.42405100
H	1.94368300	-3.09312300	-0.19392700
H	0.71134000	-3.13719100	1.06723800
H	-1.77247200	2.84837700	-2.92058400
H	-2.13767500	1.35437300	-2.05217400
H	-0.77856500	1.40561100	-3.17406300

Table S20: Coordinates for (iso)‡-[Ru^{I}(dmbpy)$(CO)_2$DMF]$^{\bullet +}$

C	-2.27395900	-0.07683200	-0.84443800
C	-3.51826100	-0.34215200	-1.38794400
N	-1.39202200	-1.06003500	-0.58319100
C	-1.82657700	1.30867700	-0.56103800
C	-3.83756700	-1.64728500	-1.71678500
C	-1.68156000	-2.32178800	-0.92904100
C	-2.46329400	2.39879500	-1.12701300
N	-0.73807600	1.45664300	0.21842100
C	-2.90334600	-2.63984900	-1.50979500
C	-1.97097500	3.66655100	-0.88098900
C	-0.23682800	2.68226800	0.44515400
C	-0.84789700	3.80664400	-0.09489500
H	-4.23501800	0.44784900	-1.54902800
H	-3.31689000	2.26627200	-1.77289100
Ru	0.13762100	-0.37048900	0.98072400
C	-0.05750100	0.10182000	2.73154300
O	-0.22151600	0.39024200	3.83305300
C	0.89182400	-1.93781400	1.65123700
O	1.37614500	-2.86696800	2.10487300
H	3.45039200	0.93235900	-3.51233100
H	4.99981900	-1.05086000	-2.93549400
C	2.85233000	-0.39322700	-0.76325800
O	1.78022200	0.18722200	-0.52767500
C	3.51142300	1.20342800	-2.45735800
N	3.73267900	0.01308000	-1.65794200
C	4.96205000	-0.71906600	-1.89714200
H	5.01113100	-1.58773900	-1.24438400
H	2.58555800	1.67877700	-2.14880700
H	4.34317700	1.89478500	-2.31797000
H	5.82063100	-0.07606800	-1.70012800
H	3.13686600	-1.30299800	-0.22565100
C	-0.66474200	-3.38885300	-0.67833700
C	0.99215100	2.82766600	1.28518600
H	-4.80445800	-1.88075100	-2.14186900
H	-2.45242000	4.53263400	-1.31454300
H	0.34193500	-3.01985100	-0.86928000
H	-0.85142700	-4.24958100	-1.31743000
H	-0.71843800	-3.72629700	0.35827000
H	-0.42706900	4.78197500	0.10319900
H	-3.11090900	-3.66440100	-1.78438600
H	1.77133900	2.14454200	0.94991300
H	0.77329000	2.60790400	2.33097100
H	1.36668200	3.84727500	1.22642300

Table S21: Coordinates for $[Ru^{I}(dmbpy)(CO)_2Cl^{(awm)}DMF]^{\bullet}$

C	1.34036800	0.83478500	1.47152600
C	1.73427700	1.63989800	2.57724000
N	0.85477900	1.44145100	0.31335100
C	1.48441200	-0.58843100	1.45677900
C	1.74206300	3.01040300	2.46957200
C	0.93713700	2.79731300	0.18897600
C	2.03307800	-1.32065500	2.54720600
N	1.12813700	-1.25647200	0.28557600
C	1.37798700	3.60046300	1.23484400
C	2.31080500	-2.66037800	2.41371200
C	1.47546700	-2.56686600	0.13624600
C	2.06692100	-3.28747800	1.16793100
H	2.05166200	1.16475900	3.50463400
H	2.25567900	-0.81090500	3.48382400
Ru	-0.08097000	-0.00281700	-0.98282000
C	-0.91112000	-1.35146800	-2.00822600
O	-1.47457600	-2.10558200	-2.66765600
C	-1.16125200	1.17268700	-1.98639800
O	-1.85990000	1.81206800	-2.63834000
C	0.54575300	3.45333100	-1.10843600
H	1.43007900	4.68061000	1.09233600
C	1.21962400	-3.26207200	-1.17451300
H	2.32970700	-4.33354000	1.00550200
H	0.91612400	2.87921900	-1.96915400
H	0.96191400	4.46901300	-1.15562900
H	-0.54724700	3.54888300	-1.19918700
H	1.46251100	-2.60707700	-2.02268900
H	0.16833700	-3.57593200	-1.26702500
H	1.83305900	-4.17100600	-1.24290000
H	2.73854200	-3.22486000	3.24561900
H	2.05185800	3.63276800	3.31234100
O	-1.47903400	-0.17492400	0.64653600
C	-2.72801700	-0.22430300	0.60651000
H	-3.27601100	-0.17724000	-0.35069000
N	-3.49757400	-0.33633500	1.68381300
C	-4.94983700	-0.38929800	1.56980700
C	-2.93224300	-0.41194200	3.02618000
H	-5.24770600	-0.32085300	0.51570800
H	-5.40454900	0.44632800	2.12446800
H	-5.32908000	-1.33508900	1.98754100
H	-1.83979500	-0.36795200	2.96571900
H	-3.23691500	-1.35474300	3.50680500
H	-3.30150600	0.42816300	3.63511000
Cl	1.71436200	0.19320500	-2.59797100

Table S22: Coordinates for $[Ru^{I}(dmbpy)(CO)_2Cl\ DMF^{(awm)}]^{\bullet}$

C	-2.08792300	-0.70674600	-0.83394500
C	-3.13576200	-1.47945300	-1.39122600
N	-0.97167800	-1.33839900	-0.31511400
C	-2.08760400	0.70762600	-0.83392800
C	-2.99606000	-2.82614600	-1.52117500
C	-0.82050800	-2.66847800	-0.50969100
C	-3.13501600	1.48079500	-1.39136400
N	-0.97114600	1.33877500	-0.31494300
C	-1.79284000	-3.43192500	-1.11325800
C	-2.99474400	2.82743800	-1.52120300
C	-0.81942800	2.66881800	-0.50935800
C	-1.79136600	3.43270600	-1.11300000
H	-4.03894800	-0.99679500	-1.73286800
H	-4.03830200	0.99851200	-1.73326500
Ru	0.20846600	-0.00013100	0.86962100
C	1.11225300	1.24736400	1.93919200
O	1.65480600	1.92759600	2.67281500
C	1.11169800	-1.24818100	1.93900000
O	1.65398300	-1.92876300	2.67249800
H	3.32409500	-0.88823300	-3.68006900
H	5.40954600	-0.88769000	-2.19863200
C	2.80285900	-0.00013000	-0.73119800
O	1.55747100	-0.00031600	-0.76861800
C	2.98857300	-0.00101500	-3.14218100
N	3.55882400	-0.00044400	-1.80696000
C	5.00707400	0.00006100	-1.70993400
H	5.31022800	0.00057900	-0.66561800
H	1.90553200	-0.00239800	-3.07459400
H	3.32182000	0.88713300	-3.67996500
H	5.40896700	0.88768800	-2.19933400
H	3.34406600	0.00030200	0.21752900
C	0.44288000	-3.33688300	-0.05829700
H	-1.62518500	-4.48967500	-1.25685300
C	0.44412800	3.33669800	-0.05765900
H	-1.62327600	4.49040600	-1.25645800
H	1.31686100	-2.72354100	-0.27418400
H	0.55828900	-4.29211500	-0.56711300
H	0.41623700	-3.54075200	1.01265300
H	1.31792700	2.72308900	-0.27351500
H	0.41738800	3.54038500	1.01332400
H	0.55995200	4.29197900	-0.56628900
H	-3.78837300	3.42254100	-1.95467000
H	-3.79002200	-3.42090200	-1.95450300
Cl	-1.56533400	0.00013500	2.49188400

Table S23: Coordinates for DMF

H	0.97558600	1.76016500	0.88778100
C	0.43713000	1.42128300	0.00001300
H	-0.56196900	1.84781400	-0.00157000
O	-1.94776300	-0.08690900	-0.00002600
H	2.16298300	-0.52639000	0.88709500
H	0.97837800	1.76000300	-0.88609900
N	0.33428100	-0.02141800	-0.00014100
C	1.57311600	-0.76794500	0.00012800
H	2.16332000	-0.52644800	-0.88662500
C	-0.85605000	-0.63669900	-0.00003300
H	-0.76329400	-1.73386600	-0.00016600
H	1.36195500	-1.83591200	0.00013000

Table S24: Coordinates for BNAH$^{\bullet+}$

H	-5.14035500	-0.96272200	1.70357900
C	-4.30818600	-0.73409700	1.05031100
C	-4.36117700	0.37491000	0.22079800
H	-5.23348500	1.01541200	0.22304600
C	-3.29308800	0.66758700	-0.61592300
H	-3.33720300	1.53432200	-1.26461400
C	-2.17144800	-0.15080600	-0.62923100
C	-1.00840300	0.16647300	-1.53461200
C	-2.12353600	-1.26521000	0.20324100
H	-1.25353000	-1.91168900	0.19648600
C	-3.18720800	-1.55459300	1.04084100
H	-3.14460300	-2.42377100	1.68421800
N	0.17639500	0.57875300	-0.75816700
C	1.26622900	-0.21730900	-0.70388700
C	0.12445700	1.76111700	-0.06747700
C	2.36001300	0.10695900	0.03555500
C	1.16144200	2.16896000	0.68516200
C	2.39658800	1.36857900	0.81391200
C	3.49706000	-0.87338900	0.00597600
O	3.41264700	-1.89725700	-0.65362800
N	4.57660000	-0.55399900	0.73372300
H	4.65459500	0.28651200	1.27612900
H	5.35714300	-1.18827700	0.73708900
H	3.25628800	1.98599800	0.51149300
H	2.58136700	1.16435200	1.87947800
H	1.09249700	3.10907700	1.21263700
H	-0.78965100	2.32599600	-0.16900200
H	1.23662900	-1.13240500	-1.27656500
H	-1.25104600	0.98308800	-2.21307400
H	-0.71725700	-0.69619300	-2.12876100

Table S25: Coordinates for BNA^{+}

H	5.14505000	-1.01257100	-1.65004600
C	4.30671200	-0.76838100	-1.01044000
C	4.32312200	0.39594700	-0.25907300
H	5.17316400	1.06391000	-0.30772400
C	3.24732800	0.70775800	0.56048900
H	3.26521000	1.61633100	1.15043400
C	2.15317900	-0.14473200	0.63379800
C	0.98289700	0.19767300	1.51503900
C	2.14450800	-1.31651000	-0.11737400
H	1.30016200	-1.99353000	-0.05841400
C	3.21571900	-1.62535300	-0.93830500
H	3.20315900	-2.53859600	-1.51884800
N	-0.20528200	0.59938800	0.71384100
C	-1.29432000	-0.17215800	0.69663500
C	-0.15024000	1.73749000	0.00018600
C	-2.39171100	0.16143300	-0.07051700
C	-1.21732800	2.13234800	-0.76351200
C	-2.35160000	1.33502400	-0.80749000
C	-3.57222500	-0.77433300	0.00699400
O	-3.72122000	-1.47503600	0.99252100
N	-4.40927900	-0.76755900	-1.03850200
H	-4.20620700	-0.29135400	-1.89828800
H	-5.19684900	-1.39330400	-1.02429600
H	-3.19910500	1.65303500	-1.39971000
H	-1.15959800	3.05711500	-1.31683300
H	0.76589700	2.30484600	0.06897300
H	-1.28500900	-1.06239900	1.30741400
H	1.21331300	1.02738500	2.17976700
H	0.67625400	-0.65063200	2.12144000

Table S26: Coordinates for $Ru^{II}(4dmbpy^{\bullet -})(CO)_2Cl_2$ in DMSO (PCM)

C	-1.55509500	-0.70664900	0.00004100
C	-2.72817000	-1.50877600	0.00004800
N	-0.32250100	-1.32375700	0.00019100
C	-1.55507300	0.70668500	-0.00008900
C	-2.65974400	-2.87174800	0.00019700
C	-0.26975500	-2.66203300	0.00036000
C	-2.72812200	1.50884900	-0.00024300
N	-0.32245900	1.32375200	-0.00004200
C	-1.37385100	-3.47068000	0.00037000
C	-2.65965100	2.87181900	-0.00029100
C	-0.26966900	2.66202600	-0.00006400
C	-1.37373900	3.47070900	-0.00017600
H	-3.69729100	-1.02867200	-0.00005200
H	0.72038900	-3.09954200	0.00050300
H	-3.69725900	1.02877700	-0.00032500
H	0.72048800	3.09950400	0.00000600
Ru	1.32579700	-0.00002600	0.00004300
Cl	1.23295500	0.00023900	2.43361800
Cl	1.23290800	-0.00029300	-2.43354900
C	2.66437600	1.30884300	-0.00012700
O	3.47780500	2.10679800	-0.00026100
C	2.66425200	-1.30901300	0.00023800
O	3.47794200	-2.10670200	-0.00003700
H	-1.25573600	-4.54556200	0.00051600
H	-1.25558800	4.54558700	-0.00018200
C	-3.88748600	3.73134300	-0.00051700
C	-3.88760700	-3.73123200	0.00013500
H	-4.79671000	3.13087800	-0.00008300
H	-3.90293600	4.38052000	-0.87917800
H	-3.90265900	4.38141700	0.87748000
H	-4.79681100	-3.13073700	0.00050200
H	-3.90277800	-4.38117300	0.87823100
H	-3.90310100	-4.38054100	-0.87842800

Table S27: Coordinates for $Ru^{II}(5dmbpy^{\bullet-})(CO)_2Cl_2$ in DMSO (PCM)

C	0.70728500	-1.77970100	-0.00007700
C	1.51266100	-2.94678800	-0.00008200
N	1.32566100	-0.55013100	-0.00014300
C	-0.70727800	-1.77970200	0.00000900
C	2.86988800	-2.85203800	-0.00015600
C	2.66953300	-0.48898800	-0.00022500
C	-1.51265000	-2.94679200	0.00001800
N	-1.32565700	-0.55013400	0.00006900
C	3.49800100	-1.57949900	-0.00023500
C	-2.86987700	-2.85204600	0.00008400
C	-2.66952900	-0.48899400	0.00013000
C	-3.49799500	-1.57950900	0.00014300
H	1.04387400	-3.92070400	-0.00001700
H	3.47580700	-3.75073800	-0.00015200
H	3.09658400	0.50665100	-0.00028900
H	-1.04386100	-3.92070700	-0.00003900
H	-3.47579400	-3.75074800	0.00008500
H	-3.09658300	0.50664200	0.00016900
Ru	-0.00000100	1.09643200	0.00001800
Cl	0.00019800	1.00420100	2.43451900
Cl	-0.00020000	1.00436000	-2.43448300
C	-1.30850900	2.43517600	0.00012000
O	-2.10583100	3.24943800	0.00033300
C	1.30851600	2.43516700	-0.00002400
O	2.10579300	3.24947300	-0.00007900
C	4.99101500	-1.44414500	-0.00031600
C	-4.99100900	-1.44415800	0.00021400
H	5.43200200	-1.92103100	-0.87952800
H	5.43208300	-1.92088100	0.87893900
H	5.29254400	-0.39620200	-0.00041800
H	-5.43207700	-1.92097200	-0.87899700
H	-5.29254100	-0.39621600	0.00022700
H	-5.43199400	-1.92096900	0.87947000

Table S28: Coordinates for $Ru^{II}(6dmbpy^{\bullet-})(CO)_2Cl_2$ in DMSO (PCM)

C	0.70843000	1.95016400	0.06042700
C	1.47464600	3.13387800	0.21144400
N	1.34941300	0.74211500	-0.15031300
C	-0.70628900	1.95089900	0.06036900
C	2.82434900	3.10185600	0.04931100
C	2.68380000	0.73731900	-0.36286800
C	-1.47132500	3.13535100	0.21158400
N	-1.34849500	0.74354500	-0.15065000
C	3.44365500	1.88363000	-0.28451900
C	-2.82103700	3.10474200	0.04922400
C	-2.68283700	0.74015700	-0.36344700
C	-3.44153000	1.88723400	-0.28502900
H	0.98478600	4.06854400	0.43823600
H	3.41134600	4.00536200	0.15627700
C	3.37023200	-0.55382000	-0.69058800
H	-0.98055600	4.06943200	0.43881900
H	-3.40713700	4.00880700	0.15638300
C	-3.37052000	-0.55024900	-0.69145900
Ru	-0.00049100	-0.92461800	0.07523000
Cl	-0.00033900	-1.23613200	-2.33651900
Cl	-0.00050900	-0.44895700	2.46432300
C	-1.23350500	-2.29817400	0.35117500
O	-1.90363900	-3.19568500	0.56221100
C	1.23078100	-2.29968400	0.35142400
O	1.89961000	-3.19814500	0.56256500
H	4.50718100	1.83038700	-0.46802300
H	-4.50507400	1.83511800	-0.46874800
H	2.79486400	-1.13397300	-1.41141500
H	3.51365000	-1.15597000	0.20746200
H	4.35500800	-0.35409100	-1.10935700
H	-2.79549800	-1.13100900	-1.41207000
H	-4.35491700	-0.34945700	-1.11061500
H	-3.51494700	-1.15225700	0.20652600

References

(1) Chardon-Noblat, S.; Deronzier, A.; Ziessel, R.; Zsoldos, D. Selective Synthesis and Electrochemical Behavior of trans(Cl)- and cis(Cl)-[Ru(bpy)(CO)2Cl2] Complexes (bpy = 2,2‘-Bipyridine). Comparative Studies of Their Electrocatalytic Activity toward the Reduction of Carbon Dioxide. *Inorg. Chem.* **1997**, *36*, 5384–5389.

(2) Haukka, M.; Kiviaho, J.; Ahlgren, M.; Pakkanen, T. A. Studies on Catalytically Active Ruthenium Carbonyl Bipyridine Systems. Synthesis and Structural Characterization of [Ru(bpy)$(CO)_2Cl_2$], [Ru(bpy)$(CO)_2$Cl(C(O)OCH_3)], [Ru(bpy)$(CO)_2$Cl$]_2$, and [Ru(bpy)$(CO)_2$ClH](bpy=2,2’-Bipyridine). *Organometallics* **1995**, *14*, 825–833.

(3) Homanen, P.; Haukka, M.; Ahlgrén, M.; Pakkanen, T. A.; Baxter, P. N.; Benfield, R. E.; Connor, J. A. Ruthenium Bipyridine Complexes: Synthesis and Characterisation of Ru (tmbpy)(CO) 2Cl2, Ru (dmbpy)(CO) 2Cl2 and [Ru (dmbpy)(CO) 2Cl] 2. *J. Organomet. Chem.* **1998**, *552*, 205–211.

(4) Hadda, T. B.; Zidane, I.; Moya, S. A.; Le Bozec, H. Soluble Ruthenium Carbonyl Complexes Containing New Sterically Hindered Bipyridyne Ligands. *Polyhedron* **1996**, *15*, 1571–1573.

(5) Kuramochi, Y.; Itabashi, J.; Toyama, M.; Ishida, H. Photochemical CO_2 Reduction Catalyzed by Trans(Cl)-[Ru(2,2’-bipyridine)$(CO)_2Cl_2$] Bearing Two Methyl Groups at 4,4’-, 5,5’- or 6,6’- Positions in the Ligand. *ChemPhotoChem* **2018**, *2*, 314–322.

(6) Anderson, P. A.; Deacon, G. B.; Haarmann, K. H.; Keene, F. R.; Meyer, T. J.; Reitsma, D. A.; Skelton, B. W.; Strouse, G. F.; Thomas, N. C. Designed Synthesis of Mononuclear Tris(heteroleptic) Ruthenium Complexes Containing Bidentate Polypyridyl Ligands. *Inorg. Chem.* **1995**, *34*, 6145–6157.

(7) Wang, P.; Liu, H.; Zhao, Q.; Chen, Y.; Liu, B.; Zhang, B.; Zheng, Q. Syntheses and

Evaluation of Drug-Like Properties of CO-Releasing Molecules Containing Ruthenium and Group 6 Metal. *Eur. J. Med. Chem.* **2014**, *74*, 199–215.

(8) Homanen, P.; Haukka, M.; Luukkanen, S. . i. e. j.; Ahlgrén, M.; Pakkanen, T. A. Selective Formation of cis(X)- and trans(X)-Ru(dmbpy)$(CO)_2X_2$ Complexes (X= Cl, Br, I, SCN) from Monomeric and Dimeric Ru-mono(dmbpy) Carbonyl Complexes (Dmbpy= 4,4'-Dimethyl-2,2'-bipyridine). *Eur. J. Inorg. Chem.* **1999**, *1999*, 101–106.

(9) Machan, C. W.; Sampson, M. D.; Kubiak, C. P. A Molecular Ruthenium Electrocatalyst for The Reduction of Carbon Dioxide to CO and Formate. *J. Am. Chem. Soc.* **2015**, *137*, 8564–8571.

(10) Chardon-Noblat*, S.; Da Costa, P.; Deronzier*, A.; Haukka, M.; Pakkanen, T. A.; Ziessel, R. Electropolymerization of [Ru(bpy)(CO)2Cl2] (bpy=2,2'-bipyridine: a revisited Trans Versus Cis(Cl) Isomeric Influence Study. *J. Electroanal. Chem.* **2000**, *490*, 62–69.

(11) Helbing, J.; Hamm, P. Versatile Femtosecond Laser Synchronization for Multiple-Timescale Transient IR Spectroscopy. *J. Chem. Phys. A* **2023**, *127*, 6347–6356.

(12) Hamm, P.; Kaindl, R. A.; Stenger, J. Noise Suppression in Femtosecond Mid-Infrared Light Sources. *Opt. Lett.* **2000**, *25*, 1798–1800.

(13) Farrell, K. M.; Ostrander, J. S.; Jones, A. C.; Yakami, B. R.; Dicke, S. S.; Middleton, C. T.; Hamm, P.; Zanni, M. T. Shot-to-Shot 2D IR Spectroscopy at 100 kHz Using a Yb Laser and Custom-Designed Electronics. *Opt. Express* **2020**, *28*, 33584–33602.

(14) Buhrke, D.; Ruf, J.; Heckmeier, P.; Hamm, P. A Stop-Flow Sample Delivery System for Transient Spectroscopy. *Rev. Sci. Instrum.* **2021**, *92*, 123001.

(15) Gabrielsson, A.; Záliš, S.; Matousek, P.; Towrie, M.; Vlček, A. Ultrafast Photochemical Dissociation of an Equatorial CO Ligand from trans (X, X)-[Ru (X) 2 (CO) 2 (bpy)](X=

Cl, Br, I): A Picosecond Time-Resolved Infrared Spectroscopic and DFT Computational Study. *Inorg. Chem.* **2004**, *43*, 7380–7388.

(16) Kubeil, M.; Vernooij, R. R.; Kubeil, C.; Wood, B. R.; Graham, B.; Stephan, H.; Spiccia, L. Studies of Carbon Monoxide Release from Ruthenium (II) Bipyridine Carbonyl Complexes upon UV-Light Exposure. *Inorg. Chem.* **2017**, *56*, 5941–5952.

(17) Eskelinen, E.; Haukka, M.; Venäläinen, T.; Pakkanen, T. A.; Wasberg, M.; Chardon-Noblat, S.; Deronzier, A. Light-Induced Decarbonylation, Solvolysis, and Isomerization of Ru (L)(CO) 2Cl2 (L= 2,2'-Bipyridine and 4,4'-Dimethyl-2,2'-bipyridine) in Acetonitrile. *Organometallics* **2000**, *19*, 163–169.

(18) Ohnishi, Y.; Kitami, M. One-electron Reduction of 1-Benzyl-3-carbamoylpyridinium as a NAD+ Model. *Bull. Chem. Soc. Jpn.* **1979**, *52*, 2674–2677.

(19) Frisch, M. J. et al. Gaussian~16 Revision A.03. 2016; Gaussian Inc. Wallingford CT.

(20) Chai, J.-D.; Head-Gordon, M. Long-Range Corrected Hybrid Density Functionals with Damped Atom-Atom Dispersion Corrections. *PCCP* **2008**, *10*, 6615–6620.

(21) Weigend, F.; Ahlrichs, R. Balanced Basis Sets of Split Valence, Triple Zeta Valence and Quadruple Zeta Valence Quality for H to Rn: Design and Assessment of Accuracy. *PCCP* **2005**, *7*, 3297–3305.

(22) Mennucci, B. Polarizable Continuum Model. *Wiley Interdiscip. Rev.: Comput. Mol. Sci.* **2012**, *2*, 386–404.

(23) Ochterski, J. W. Thermochemistry in Gaussian. *Gaussian Inc* **2000**, *1*.

(24) Peng, C.; Bernhard Schlegel, H. Combining Synchronous Transit and Quasi-Newton Methods to Find Transition States. *Isr. J. Chem.* **1993**, *33*, 449–454.

(25) Peng, C.; Ayala, P. Y.; Schlegel, H. B.; Frisch, M. J. Using Redundant Internal Coordinates to Pptimize Equilibrium Geometries and Transition States. *J. Comput. Chem.* **1996**, *17*, 49–56.

(26) Homanen, P.; Haukka, M.; Pakkanen, T. A.; Pursiainen, J.; Laitinen, R. H. Ruthenium(II) Bipyridine Complexes: Synthesis and Characterization of Ru(bpy$(CO)_2(SCN)_2$, Ru(dmbpy)$(CO)_2Cl_2$, and Ru(dmbpy)$(CO)_2$(NCS)H (bpy=2,2'-Bipyridine; dmbpy=6,6'-Dimethyl-2,2'-bipyridine). *Organometallics* **1996**, *15*, 4081–4084.
